# The Alzheimer’s disease–linked protease BACE2 cleaves VEGFR3 and modulates its signaling

**DOI:** 10.1172/JCI170550

**Published:** 2024-06-18

**Authors:** Andree Schmidt, Brian Hrupka, Frauke van Bebber, Sanjay Sunil Kumar, Xiao Feng, Sarah K. Tschirner, Marlene Aßfalg, Stephan A. Müller, Laura Sophie Hilger, Laura I. Hofmann, Martina Pigoni, Georg Jocher, Iryna Voytyuk, Emily L. Self, Mana Ito, Kana Hyakkoku, Akimasa Yoshimura, Naotaka Horiguchi, Regina Feederle, Bart De Strooper, Stefan Schulte-Merker, Eckhard Lammert, Dieder Moechars, Bettina Schmid, Stefan F. Lichtenthaler

**Affiliations:** 1 German Center for Neurodegenerative Diseases (DZNE), Munich, Germany.; 2 Neuroproteomics, School of Medicine and Health, Klinikum Rechts der Isar, Technical University of Munich, Munich, Germany.; 3 Graduate School of Systemic Neurosciences (GSN), Ludwig Maximilian University (LMU) Munich, Munich, Germany.; 4 Evotec München, Neuried, Germany.; 5 Discovery Neuroscience, Janssen Pharmaceutica NV, a Johnson & Johnson Company, Beerse, Belgium.; 6 Institute of Cardiovascular Organogenesis and Regeneration, Faculty of Medicine, WU Münster, Münster, Germany.; 7 Faculty of Mathematics and Natural Sciences, Institute of Metabolic Physiology, and; 8 International Research Training Group (IRTG1902), Heinrich-Heine-University, Düsseldorf, Germany.; 9 Laboratory for the Research of Neurodegenerative Diseases, Department of Neurosciences, Leuven Brain Institute (LBI), KU Leuven (University of Leuven), Leuven, Belgium.; 10 Vlaams Instituut voor Biotechnologie (VIB) Center for Brain and Disease Research, VIB, Leuven, Belgium.; 11 MRC Toxicology Unit, University of Cambridge, Cambridge, United Kingdom.; 12 Shionogi & Co., Laboratory for Drug Discovery and Disease Research, Shionogi Pharmaceutical Research Center, Toyonaka-shi, Osaka, Japan.; 13 Core Facility Monoclonal Antibodies, Helmholtz Zentrum München, German Research Center for Environmental Health, Neuherberg, Germany.; 14 Munich Cluster for Systems Neurology (SyNergy), Munich, Germany.; 15 UK Dementia Research Institute (UKDRI) at University College London, London, United Kingdom.; 16 Institute for Vascular and Islet Cell Biology, German Diabetes Center, Leibniz Center for Diabetes Research at Heinrich Heine University, Düsseldorf, Germany.; 17 German Center for Diabetes Research (DZD e.V.), Neuherberg, Germany.

**Keywords:** Aging, Alzheimer disease, Drug therapy

## Abstract

The β-secretase β-site APP cleaving enzyme (BACE1) is a central drug target for Alzheimer’s disease. Clinically tested, BACE1-directed inhibitors also block the homologous protease BACE2. Yet little is known about physiological BACE2 substrates and functions in vivo. Here, we identify BACE2 as the protease shedding the lymphangiogenic vascular endothelial growth factor receptor 3 (VEGFR3). Inactivation of BACE2, but not BACE1, inhibited shedding of VEGFR3 from primary human lymphatic endothelial cells (LECs) and reduced release of the shed, soluble VEGFR3 (sVEGFR3) ectodomain into the blood of mice, nonhuman primates, and humans. Functionally, BACE2 inactivation increased full-length VEGFR3 and enhanced VEGFR3 signaling in LECs and also in vivo in zebrafish, where enhanced migration of LECs was observed. Thus, this study identifies BACE2 as a modulator of lymphangiogenic VEGFR3 signaling and demonstrates the utility of sVEGFR3 as a pharmacodynamic plasma marker for BACE2 activity in vivo, a prerequisite for developing BACE1-selective inhibitors for safer prevention of Alzheimer’s disease.

## Abstract 2

The Alzheimer’s disease–linked protease BACE2 modulates lymphangiogenic VEGFR3 signaling, and BACE2-cleaved soluble VEGFR3 has utility as a pharmacodynamic plasma marker for BACE2 activity in vivo.

## Graphical abstract

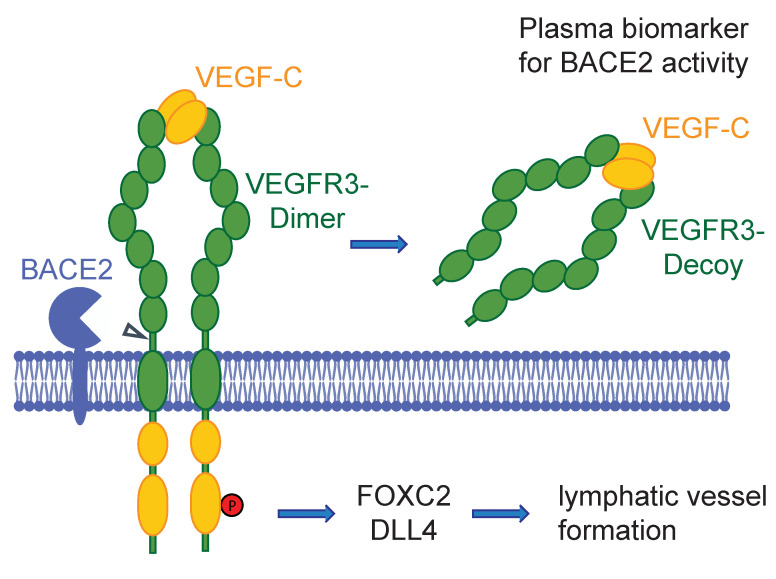

## Introduction

Alzheimer’s disease (AD) is the most common neurodegenerative disorder. Effective therapeutic or preventive approaches for this deadly disease are lacking. A central drug target for AD is the transmembrane protease BACE1 (β-site APP cleaving enzyme, β-secretase), which cleaves the amyloid precursor protein (APP) and thereby catalyzes the first step in the generation of the amyloid β (Aβ) peptide, which has a key pathogenic role early in AD pathogenesis (1).

BACE1-targeted small molecule inhibitors have advanced to phase 3 clinical trials for AD, where they efficiently lowered Aβ in brain and cerebrospinal fluid (CSF) of AD patients and individuals at high risk for AD (2). However, 5 out of 6 BACE1-targeted inhibitors induced mild cognitive worsening. This adverse event appears reversible, but led to discontinuation of these inhibitors in clinical AD trials. Additionally, the inhibitors led to a shrinkage of hippocampal volume, as detected by magnetic resonance imaging (3–5). While the underlying molecular mechanisms have not yet been identified, they are assumed to be mechanism based and to result from too strong inhibition of cleavage of one or several of the numerous BACE1 substrates and their functions (2). The side effects may also be caused by inhibition of the BACE1-homolog BACE2 because all clinically tested BACE1-targeted inhibitors also block the aspartyl transmembrane protease BACE2 with similar or even higher potency (2).

Much less is known about BACE2 compared with BACE1. While *BACE1* is highly expressed in neurons, *BACE2* is mostly expressed nonneuronally, with little expression in brain under noninflammatory conditions (6). Using *Bace2*-deficient mice, which are viable and fertile (7), 2 in vivo BACE2 functions were established in pancreas and skin. BACE2 cleaves TMEM27 and thereby controls insulin homeostasis and pancreatic β cell mass (8) and is discussed as a potential drug target for diabetes treatment. Additionally, BACE2 cleaves the pigment cell–specific melanocyte protein (PMEL) and controls pigmentation in zebrafish and mice and, partly, hair pigmentation in humans (9–11) as well as PMEL-dependent melanoma metastasis formation (12). Several additional membrane proteins have been identified as BACE2 substrates in vitro (13), including APP (14), which demonstrates that BACE2 contributes to the ectodomain shedding of membrane proteins, which controls abundance and function of membrane proteins (15). Yet the functional relevance of the cleavage of the additional substrates remains largely unclear.

The limited knowledge about in vivo–relevant BACE2 substrates and functions precludes an evaluation of whether BACE2 contributes to the side effects in the BACE inhibitor trials and whether BACE inhibitors may have additional undesired or even beneficial side effects. Another key challenge is the lack of a suitable marker rapidly reporting on target engagement of BACE2 in vivo, which could help with developing safer, BACE1-selective inhibitors for AD. In mice, BACE2 inhibition is currently monitored by hair depigmentation after chest hair removal, followed by chronic BACE2 inhibition over several weeks (9, 16). In humans, BACE2 inhibition can currently not be monitored.

Here, we identify the lymphangiogenic vascular endothelial growth factor receptor 3 (VEGFR3, also known as Fms-related receptor tyrosine kinase 4 [FLT4]) as an in vivo BACE2 substrate. We demonstrate that BACE2 cleavage modulates VEGFR3 function ex vivo in primary human cells and in vivo in zebrafish during lymphatic development. Moreover, we establish soluble, BACE2-cleaved VEGFR3 as an easily accessible pharmacodynamic marker for BACE2 activity in plasma across species.

## Results

### Plasma proteomics identifies VEGFR3 as a BACE2 substrate candidate.

**Figure 1:**
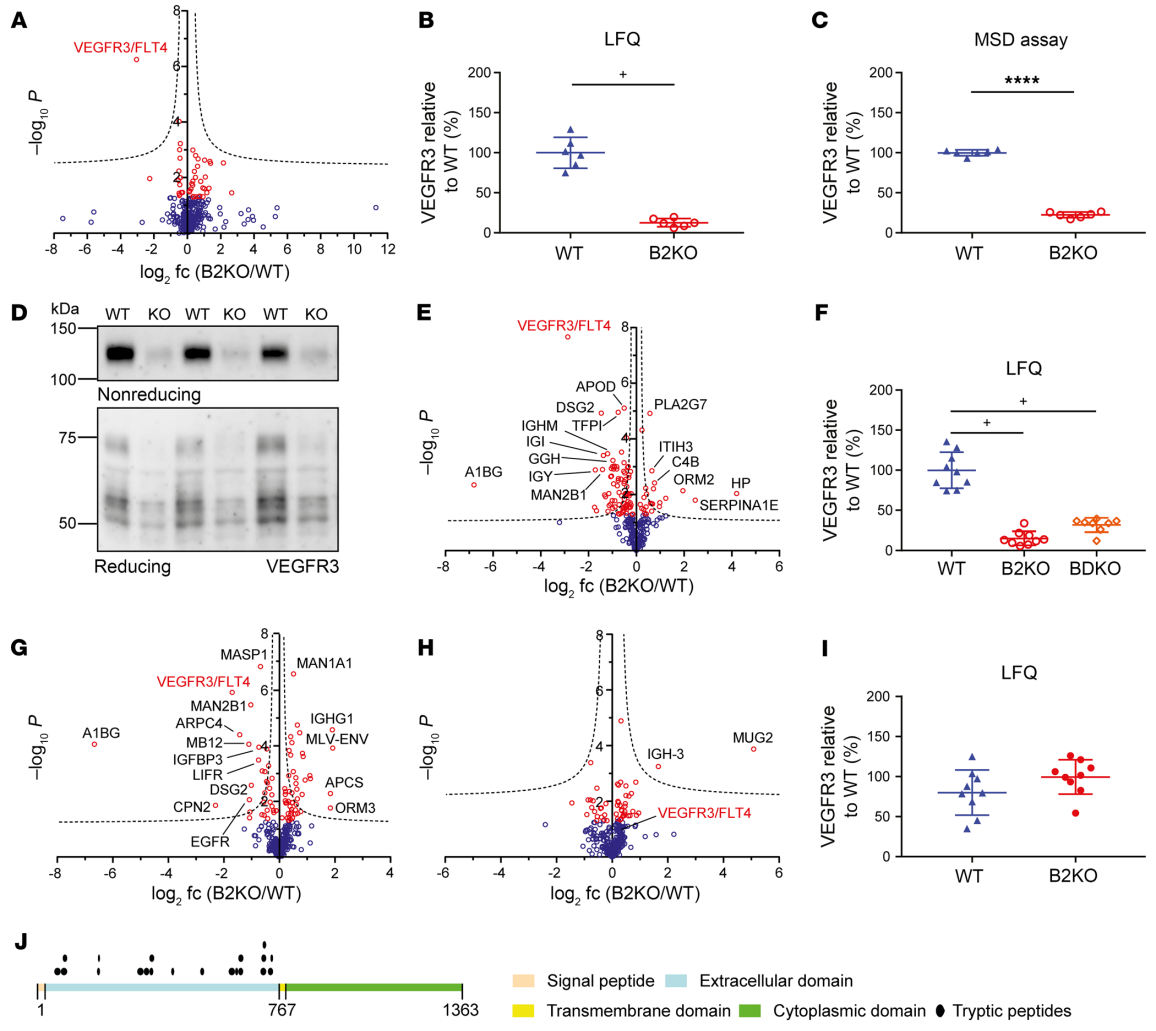
Identification of VEGFR3 as a BACE2 substrate candidate. (**A**) Volcano plot of proteomic analysis of murine plasma from WT and B2KO mice (*n* = 6). VEGFR3 (FLT4) is highlighted in red. (**B**) Normalized VEGFR3 LFQ intensities extracted from **A**. (**C**) MSD-assay quantifications of sVEGFR3 in the same plasma samples. (**D**) Immunoblot detection of sVEGFR3 ectodomain in mouse plasma from **A**, using nonreducing and reducing conditions. (**E**) Volcano plot of proteomic analysis of murine plasma from an independent B2KO line (*n* = 9) compared with WT (*n* =9) and (**F**) the extracted normalized LFQ values. Volcano plots of the proteomic analyses of *Bace1/Bace2* double-knockout (BDKO) mice (*n* =9) compared with the WT line (*n* = 9) (**G**) (corresponding extracted LFQ intensities of sVEGFR3 in **F**) and B1KO (*n* = 9) compared with an individual control WT line (*n* = 9) (**H**). (**I**) Normalized LFQ values extracted from **H**. (**J**) Localization of identified individual peptides (black dots) on the canonical VEGFR3 sequence. The signal peptide is shown in rose, the ectodomain is indicated in blue, the intracellular domain in green, and the transmembrane domain in yellow. Two sided Student’s *t* tests with a permutation-based FDR correction (FDR < 0.05; indicated by hyperbolic curves) were used for volcano plots (**A**, **E**, **G**, and **H**). Proteins with *P* < 0.05 are shown as red circles. Extracted LFQ quantifications (**B**, **F**, and **I**) of VEGFR3 with significance after FDR correction are labeled with plus signs. All dot plots were normalized on the WT mean and depict mean and SD. MSD-assay data (**C**) additionally depicts the *P* value calculated by unpaired *t* test. ****P < 0.0001.

To identify in vivo–relevant BACE2 substrates suitable as BACE2 activity markers, we performed plasma proteomics of WT and BACE2-deficient mice (*Bace2* KO) (6). In total, we quantified 433 protein groups in plasma (Figure 1A). After multiple-testing correction, a single protein, the receptor tyrosine kinase (RTK) VEGFR3, showed a significant, approximately 8-fold reduction in *Bace2*-KO compared with WT plasma (Figure 1, A and B, and Supplemental Table 1; supplemental material available online with this article; https://doi.org/10.1172/JCI170550DS1). This result was confirmed by a Meso Scale Diagnostics immunoassay (MSD-assay) selective for the shed VEGFR3 ectodomain and by immunoblot in the same samples (Figure 1, C and D). A similar proteomic result was obtained in plasma from an independently generated *Bace2*-KO line (7) (Figure 1, E and F). VEGFR3 was also reduced in plasma from mice deficient in both *Bace1* and *Bace2* (*Bace* DKO; Figure 1, F and G), but not in plasma from *Bace1*-KO mice (Figure 1, H and I). Thus, plasma levels of VEGFR3 are dependent on BACE2, but not on BACE1. The cleavage products of the known BACE2 substrates TMEM27 and PMEL17 were not or not consistently detected in plasma, presumably because of a low abundance in plasma. Murine VEGFR3 is a type I membrane protein with a large ectodomain (751 amino acids), a transmembrane domain, and a long cytoplasmic domain (567 amino acids) (Figure 1J). The identified tryptic peptides for VEGFR3 in the WT and the *Bace2-*KO mouse plasma mapped exclusively to the ectodomain, but not to the transmembrane or cytoplasmic domain (Figure 1J), as determined with the web server Quantitative Analysis of Regulated Intramembrane Proteolysis (QARIP) (17). We conclude that plasma VEGFR3 comprises the soluble VEGFR3 (sVEGFR3) ectodomain and is possibly the result of BACE2-mediated ectodomain shedding.

### BACE2 cleaves VEGFR3 within its juxtamembrane domain.

**Figure 2:**
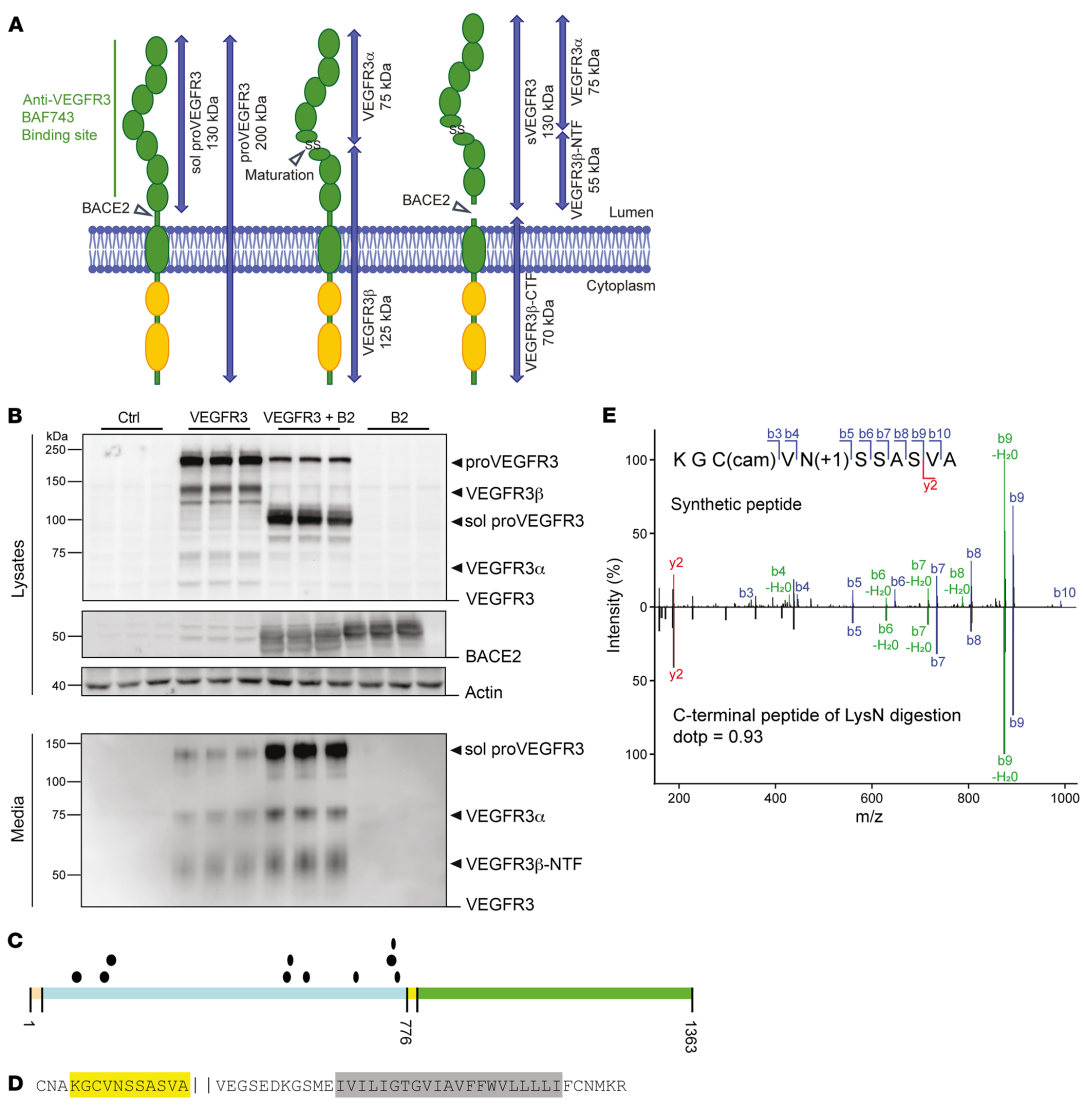
Cleavage of VEGFR3 by BACE2. (**A**) Schematic of VEGFR3 fragments. From left to right: The immature proVEGFR3 (200 kDa) can be cleaved by BACE2, releasing the immature, soluble ectodomain sol proVEGFR3 (130 kDa). The mature protein consists of 2 subunits linked through a disulfide bridge: VEGFR3α (75 kDa) and VEGFR3β (125 kDa). Upon BACE2 cleavage, the VEGFR3β-CTF (70 kDa) and sVEGFR3 (130 kDa) are generated, the latter of which consists of the VEGFR3α (75 kDa) and VEGFR3β-NTF (55 kDa) fragments. (**B**) Immunoblot detection of VEGFR3 in lysates and media of HEK293 cells transfected with empty control plasmids (Ctrl), *Vegfr3*, *Vegfr3* + *Bace2*, and *Bace2*. B2, BACE2. Data show 3 independent experiments. sVEGFR3 is not detectable under reducing conditions. sol proVEGFR3 in the lysates appears at around 100 kDa and derives from BACE2 cleavage of immaturely glycosylated proVEGFR3 early in the secretory pathway upon BACE2 overexpression. (**C**) Localization and length of identified individual peptides (black dots) on the canonical VEGFR3 sequence. The ectodomain is indicated in blue, the intracellular domain in green, the signal peptide in orange, and the transmembrane domain in yellow. (**D**) N-terminal juxtamembrane region of VEGFR3 sequence. The identified semispecific peptide after LysN digestion is marked in yellow, the proposed cleavage site after amino acid alanine with 2 vertical lines, and the transmembrane region in gray. (**E**) Comparison of the fragment ion spectra of the identified C-terminal peptide of the LysN digestion KGC(cam)VN(+1)SSASVA (lower spectrum) to a synthetic peptide with the same sequence (upper spectrum). Identified y-ions are indicated in red, b-ions in blue, and fragment ions with neutral losses in green. Both spectra match with a dot product (73) of 0.93 for the fragment ion intensities.

To determine whether BACE2 can indeed shed VEGFR3 in cells, we transfected human embryonic kidney 293 (HEK293) cells with a plasmid encoding full-length murine VEGFR3, either with or without a plasmid encoding murine BACE2. In cells, VEGFR3 is initially produced as a single polypeptide chain (proVEGFR3) with 7 Ig-like domains in the ectodomain and a split kinase domain in the cytoplasmic domain (Figure 2A). Upon maturation in the secretory pathway, VEGFR3 is proteolytically converted to a disulfide-linked covalent heterodimer comprising an N-terminal part (α-subunit, VEGFR3α) and the membrane-bound, C-terminal part (β-subunit, VEGFR3β) (18), similar to what is known for other cell-surface membrane proteins, such as the Notch receptor, LRP1, and the NRCAM cell-adhesion protein. Consequently, the full-length proVEGFR3 protein, but also the VEGFR3α and VEGFR3β subunits, were detected upon reducing immunoblot conditions compared with control-transfected cells not expressing VEGFR3 (Figure 2B and Supplemental Figure 1A). To detect all possible VEGFR3 forms and fragments after BACE2 cleavage, VEGFR3 was tagged with an N-terminal HA- and a C-terminal FLAG-epitope tag. Cotransfection of *Bace2* strongly increased cleavage of both proVEGFR3 and the mature VEGFR3α/β heterodimer, resulting in secretion of soluble proVEGFR3 and mature sVEGFR3 (Figure 2B and Supplemental Figure 1A). Additional bands of minor intensity were also observed and represent differentially glycosylated forms, as revealed by deglycosylation (Supplemental Figure 1B). The BACE2-induced increase in secretion of sVEGFR3 was blocked by verubecestat, which was clinically tested for the treatment of AD and inhibits BACE2 and BACE1 (16, 19) (Supplemental Figure 2).

sVEGFR3 was also detected by immunoblot and MSD-immunoassay in the conditioned medium of *Vegfr3*-transfected mouse insulinoma MIN6 cells (Supplemental Figure 3, A and B), which express *Bace2* endogenously (13, 20). Secretion of sVEGFR3 from MIN6 cells was blocked by verubecestat (Supplemental Figure 3A). The inhibition of VEGFR3 cleavage by verubecestat occurred in a dose-dependent manner with an IC_50_ value (2 nM) similar to the IC_50_ of BACE2 inhibition (1.8 nM) using an enzymatic in vitro assay. As expected, verubecestat also blocked BACE1 with a similar IC_50_ in a cellular assay, where the BACE1-dependent generation of the Aβ42 peptide in human neuroblastoma SK-N-BE cells was used as a readout for BACE1 activity (Supplemental Figure 3B). As a control, we also tested LY2811376, which was previously tested in a phase 1 clinical trial (21), but preferentially blocks BACE1 over BACE2. As expected, the enzymatic dose-response curve for BACE2 was shifted to higher concentrations compared with BACE1 inhibition. In the cellular MIN6 assay, even the highest concentration of LY2811376 did not fully inhibit BACE2 (Supplemental Figure 3B). Similar results were obtained in HEK293 cells transiently transfected with *Vegfr3* and *Bace2*. Verubecestat, but not LY2811376 and C3 (another BACE1-preferring inhibitor), efficiently reduced sVEGFR3 cleavage, as seen by the reduced sVEGFR3 in the conditioned medium (Supplemental Figure 4).

To determine the cleavage site of BACE2 in VEGFR3, the sVEGFR3 ectodomain was immunoprecipitated from the conditioned medium of VEGFR3-transfected MIN6 cells. Because the membrane-proximal domain of VEGFR3, where the BACE2 cleavage site is expected, contains a putative N-glycosylation motif at Asn758, sVEGFR3 was enzymatically deglycosylated using PNGaseF before LysN digestion. Resulting peptides were separated by liquid chromatography–tandem mass spectrometry (LC-MS/MS). Protease cleavage–specific and semispecific peptides generated by LysN matched exclusively to the VEGFR3 ectodomain, as expected for a BACE2-mediated ectodomain cleavage close to the transmembrane domain of VEGFR3 (Figure 2C and Supplemental Table 1). The most C-terminal ectodomain peptide had the sequence KGCVNSSASVA (Figure 2D). Its fragmentation pattern was validated by comparison with the fragmentation of a synthetic peptide with the same sequence (Figure 2E). The peptide’s N-terminus was derived from LysN cleavage (starting with lysine), whereas the C-terminus (alanine) did not correspond to a cleavage by LysN and, thus, is the likely BACE2 cleavage of VEGFR3 within its luminal juxtamembrane domain. We also used an in vitro cleavage assay, in which a synthetic peptide encompassing the probable cleavage site was cleaved by recombinant BACE2 (Supplemental Figure 5). From this finding, together with the results from the cellular MIN6 assay, we conclude that BACE2 can directly cleave VEGFR3 within its juxtamembrane domain. Interestingly, we identified 3 cleavage sites in the peptide assay that were located 1, 3, and 5 amino acids more N-terminally than in the cellular MIN6 assay (Supplemental Figure 5). The difference may result because the VEGFR3 protein is glycosylated and contains disulphide bridges, whereas both modifications are not found in the synthetic peptide.

### Endogenous BACE2 cleaves VEGFR3 in primary lymphatic endothelial cells.

**Figure 3:**
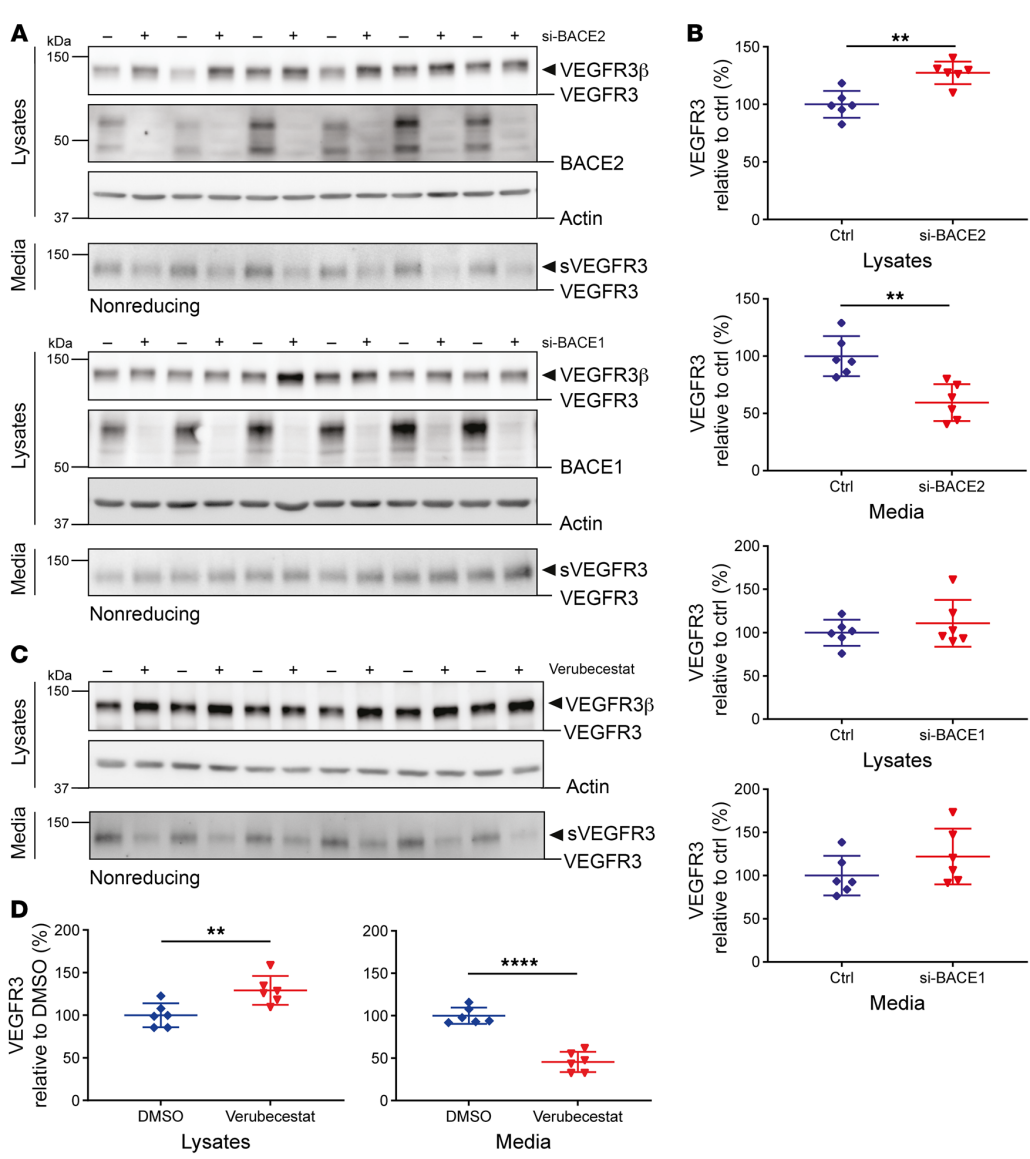
Endogenous cleavage of VEGFR3 in LECs. (**A**) Immunoblot detection after control treatment (–) or upon *BACE1* and *BACE2* knockdown (+). Lysates were blotted for VEGFR3, BACE1/2, and actin. Conditioned media were blotted for sVEGFR3. (**B**) Corresponding densitometric quantifications, deriving from VEGFR3β (lysate) and sVEGFR3 (medium). (**C**) Immunoblots of cells treated with DMSO (–) or 100 nM verubecestat (+). (**D**) Corresponding densitometric quantifications as in **B**. Dot plots were normalized on the control mean and depict mean and SD, alongside the calculated *P* values, calculated by unpaired *t* test. **P < 0.01; ****P < 0.0001. *P* values are only indicated where significance was observed. Data are derived from *n* = 6 biological replicates. Shown are representative data from 3 independent experiments.

In adult tissue, *VEGFR3* is mostly expressed in the lymphatic endothelium, but also in blood endothelial cells (22–25). Thus, to demonstrate VEGFR3 cleavage by BACE2 under physiologically relevant conditions, we used primary, human lymphatic endothelial cells (LECs), which we found to express both *VEGFR3* and *BACE2* endogenously (Figure 3A). We blocked BACE2 genetically and pharmacologically. RNAi-mediated knockdown of *BACE2*, but not *BACE1*, strongly reduced sVEGFR3 in the conditioned medium and mildly increased full-length VEGFR3 in the LEC lysate (Figure 3, A and B). Similar results were obtained with the BACE inhibitor verubecestat, which was clinically tested for treatment of AD (Figure 3, C and D), and with C3 (BACE inhibitor IV) (Supplemental Figure 6, A and B), which preferentially inhibits BACE1 but also partly inhibits BACE2 (16, 19). We conclude that BACE2 controls shedding of sVEGFR3 and abundance of full-length VEGFR3 in primary human LECs.

### Endogenous BACE2 controls signaling of VEGFR3 in primary LECs.

**Figure 4:**
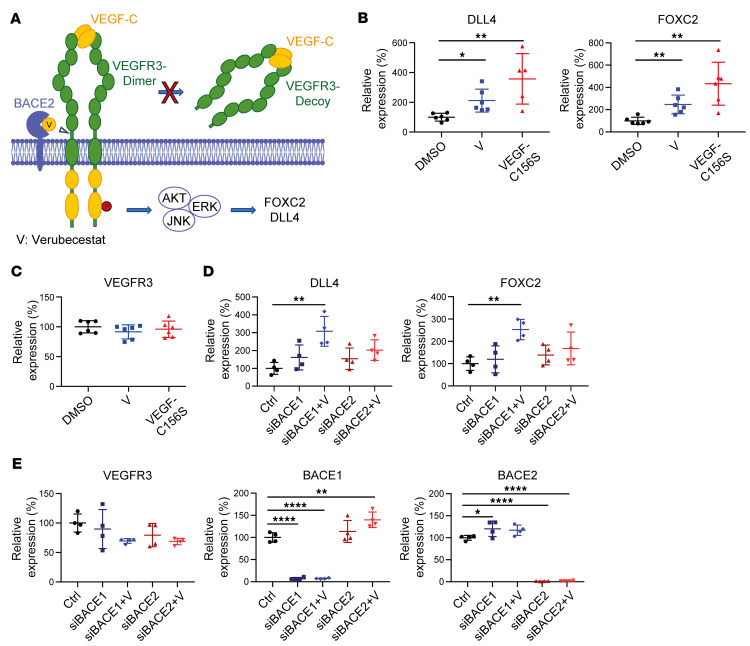
BACE2-dependent changes in VEGFR3 signaling in LECs. (**A**) Schematic for VEGFR3 signaling. Upon ligand binding, VEGFR3 dimerizes, resulting in intracellular autophosphorylation and activation of the downstream genes *FOXC2* and *DLL4*. V, verubecestat, inhibitor of BACE2. Gene expression levels of (**B**) *DLL4* and *FOXC2* and (**C**) *VEGFR3* after the application of DMSO, 100 nM verubecestat (V), and VEGF-C. (**D** and **E**) Gene expression levels of *DLL4*, *FOXC2*, *VEGFR3*, *BACE1*, and *BACE2* after *BACE* knockdown (siB1/siB2) without or with (+V) subsequent verubecestat application. All dot plots were normalized on the control mean and depict mean and SD alongside the *P* values calculated by unpaired *t* tests against the DMSO control (**B** and **C**) or by 1-way ANOVA (**D** and **E**), in both cases followed by Bonferroni’s multiple-comparison test. *P < 0.05; **P < 0.01; ****P < 0.0001. *P* values are only indicated where significance was observed. In **B**, 1 data point was excluded from the *DLL4* expression/VEGF-C156S data set, since it was identified as an outlier via the ROUT method. Data are derived from *n* = 6 (**B** and **C**) or *n* = 4 (**D** and **E**) biological replicates.

Increased abundance of RTKs may induce downstream signaling, even in the absence of ligand, and occurs in several tumors (26). VEGFR3 is an RTK and is activated by its natural ligand VEGF-C, which induces VEGFR3 dimerization and kinase signaling and results in increased expression of downstream target genes, including δ-like ligand 4 (*DLL4*) and *FOXC2* (27) (schematic in Figure 4A). With our finding that BACE2 inhibition increases abundance of full-length VEGFR3, we tested to determine whether BACE2 inhibition would induce VEGFR3 downstream signaling similar to that seen with the addition of its ligand VEGF-C (Figure 4B). In fact, short-term treatment of LEC cells for 100 minutes with the BACE inhibitor verubecestat, which blocks VEGFR3 cleavage (Figure 3C), increased RNA expression of *DLL4* and *FOXC2* by about 2-fold (Figure 4B). As a control, both treatments (verubecestat and VEGF-C) did not alter *VEGFR3* expression at the RNA level (Figure 4C). The increased expression of *DLL4* and *FOXC2* after short-term verubecestat treatment was attenuated upon RNAi-mediated knockdown of *BACE2*, but not of *BACE1* (Figure 4D), demonstrating the specific role of BACE2 in the inhibitor-mediated increase in both VEGFR3 signaling target genes. Knockdown of *BACE1* or *BACE2* efficiently reduced expression of the respective protease, but did not alter total *VEGFR3* expression (Figure 4E).

While short-term inhibition (100 minutes) of BACE2 with verubecestat increased *DLL4* and *FOXC2* expression (Figure 4B), the knockdown of *BACE2* over a period of 48 hours before treatment with verubecestat did not increase *DLL4* or *FOXC2* expression (Figure 4D), presumably because the increased expression is only seen upon acute inhibition, whereas compensatory changes may take place upon chronic BACE2 inactivation. In fact, after prolonged treatment of LECs with verubecestat for 2 days, the increased *DLL4* and *FOXC2* expression was no longer seen (Supplemental Figure 7). Together, these experiments demonstrate that BACE2-mediated VEGFR3 shedding attenuates in vitro gene expression downstream of VEGFR3 signaling.

### BACE2 controls VEGFR3 function in lymphangiogenesis in zebrafish.

To determine whether BACE2 also controls VEGFR3 function in vivo, we used zebrafish as a model. Zebrafish were previously used to reveal developmental functions of Bace1 in myelination and of Bace2 in pigmentation (11, 28, 29). Fertilized zebrafish eggs were treated for 3 days with verubecestat, which blocks both Bace1 and Bace2 (30). As expected, verubecestat reduced Bace1-dependent myelination of the posterior lateral line nerve in the peripheral nervous system, but did not alter myelination of the Mauthner axon in the central nervous system (Supplemental Figure 8, A and B), in agreement with the phenotype of *bace1*-deficient zebrafish (11, 29). Verubecestat treatment also led to mismigration of melanocytes in the tail fin (Supplemental Figure 8, C and D), similar to what occurred in a previous report using *bace2*-deficient zebrafish (11). Thus, verubecestat can functionally inhibit Bace1 and Bace2 in zebrafish.

**Figure 5:**
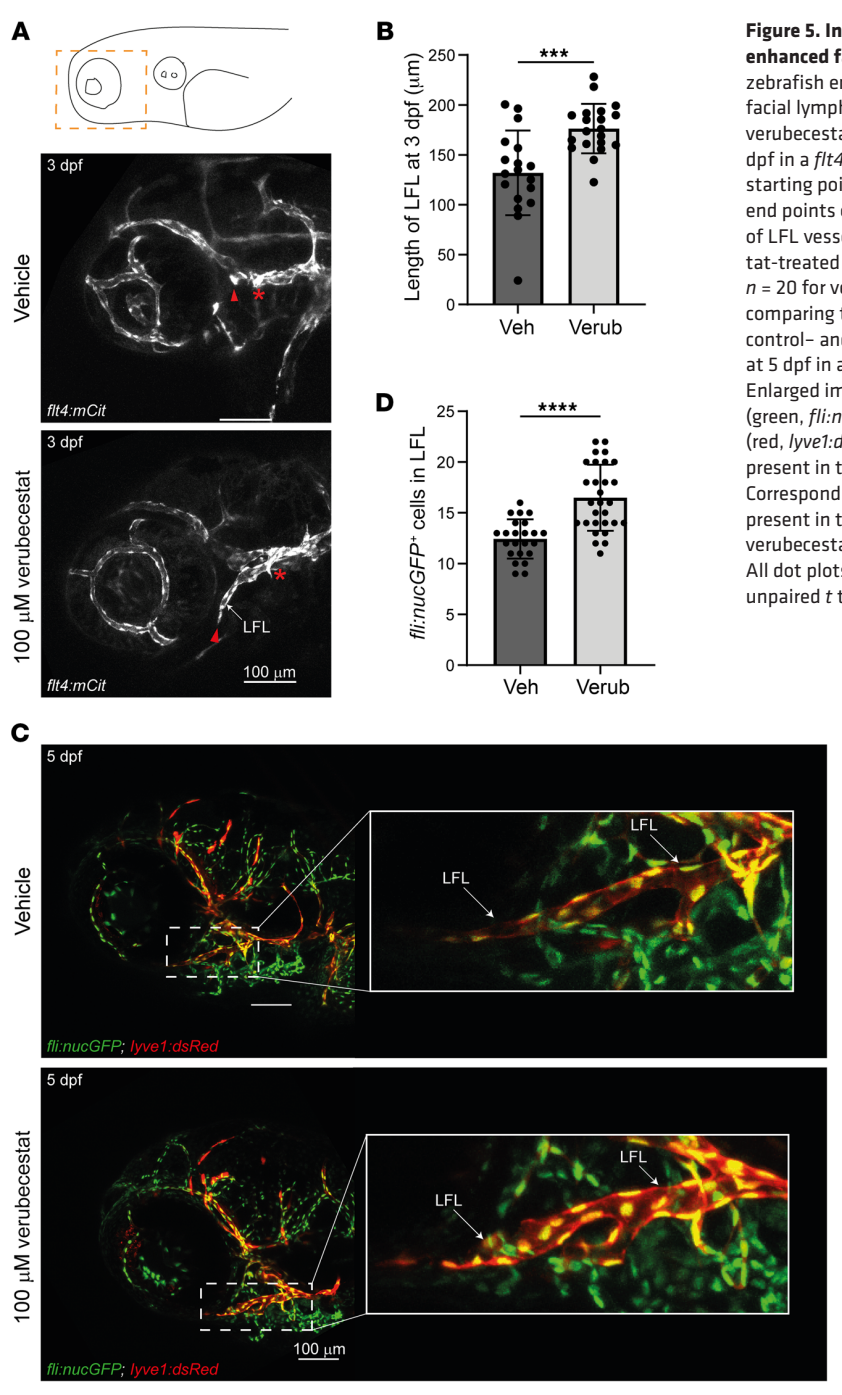
Inhibition of Bace2 in zebrafish embryos leads to enhanced facial lymphatic development. (**A**) Laterally imaged zebrafish embryos and representative images comparing the facial lymphatic development between vehicle control– (Veh) and verubecestat-treated (100 μM) (Verub) zebrafish embryos at 3 dpf in a *flt4:mCit* transgenic background. Asterisks represent the starting points of measurements, while arrowheads depict the end points of the respective measurements. (**B**) Quantification of LFL vessel length (in μm) in vehicle control– and verubecestat-treated (100 μM) zebrafish embryos at 3 dpf (*n* = 18 for vehicle, *n* = 20 for verubecestat-treated group). (**C**) Representative images comparing the facial lymphatic development between vehicle control– and verubecestat-treated (100 μM) zebrafish embryos at 5 dpf in a *fli:nucGFP* and *lyve1:dsRed* transgenic background. Enlarged image of the LFL depicts nuclei of all endothelial cells (green, *fli:nucGFP*), the cytoplasm of the facial lymphatic vessel (red, *lyve1:dsRed*) and other lymphatic vessels, and the nuclei present in the facial lymphatic vessel (yellow, colocalization). (**D**) Corresponding quantification of the number of *fli:nucGFP*^+^ nuclei present in the LFL of 5 dpf embryos (*n* = 23 for control; *n* = 29 for verubecestat-treated group). All images with anterior to the left. All dot plots depict mean and SD, alongside *P* values calculated by unpaired *t* test. ***P < 0.001; ****P < 0.0001.

Many venous and lymphatic vessels have been shown to be sensitive to loss of VEGFR3 signaling, and mutants in VEGFR3 pathway members show reduced vessel growth in mice (31, 32) and zebrafish (33, 34). We reasoned that blocking Bace2 function with verubecestat should lead to the opposite effect and focused on the lateral facial lymphatic (LFL) vessel, which can be easily monitored via life imaging (35) and which we have recently shown to specifically respond to Vegfr3/Flt4 signals (36). We used a transgenic zebrafish line expressing mCitrine under the control of the *flt4* promoter in lymphatic and venous vessels (37). Quantification and comparison of LFL length at 3 dpf revealed that verubecestat-treated zebrafish embryos displayed a significant length increase compared with the control group (Figure 5, A and B). This did not lead to a hypertrophic LFL, and at 5 dpf, control-treated embryos showed LFL structures of similar size (Figure 5C). However, at this stage, Bace2 inhibition led to a significantly higher population of endothelial cells constituting the LFL compared with the control group (Figure 5, C and D) using zebrafish expressing *GFP* in endothelial cells (*fli:nucGFP*) and DsRed in lymphatic vessels (*lyve1:dsRed*) (38). Together, these results demonstrate that verubecestat-mediated Bace2 inhibition has a mild effect on facial lymphatic development in zebrafish embryos and that this effect is opposite of Vegfr3 loss-of-function conditions in zebrafish. We conclude that loss of Bace2 activity in zebrafish increases Vegfr3 signaling, at least temporarily, and affects facial lymphatic development.

### sVEGFR3 is a pharmacodynamic activity marker for BACE2 in vivo.

BACE1-targeted inhibitors that were tested in advanced clinical trials for AD also block BACE2. However, it was not possible to quantify the degree of BACE2 inhibition in vivo. The only available readout in mice and rabbits relates to the role of BACE2 in fur pigmentation. After more than 2 weeks of continuous BACE2 inhibition, fur depigmentation (graying) becomes detectable and increases with continued dosing (9, 16). Given that our proteomic studies (Figure 1) had demonstrated that sVEGFR3 in mouse plasma was generated in a BACE2-dependent manner, we tested to determine whether plasma sVEGFR3 may be a more sensitive and faster-responding readout for changes of BACE2 activity than fur depigmentation and thus be useful as an easily measurable pharmacodynamic activity marker for BACE2 in mice.

**Figure 6:**
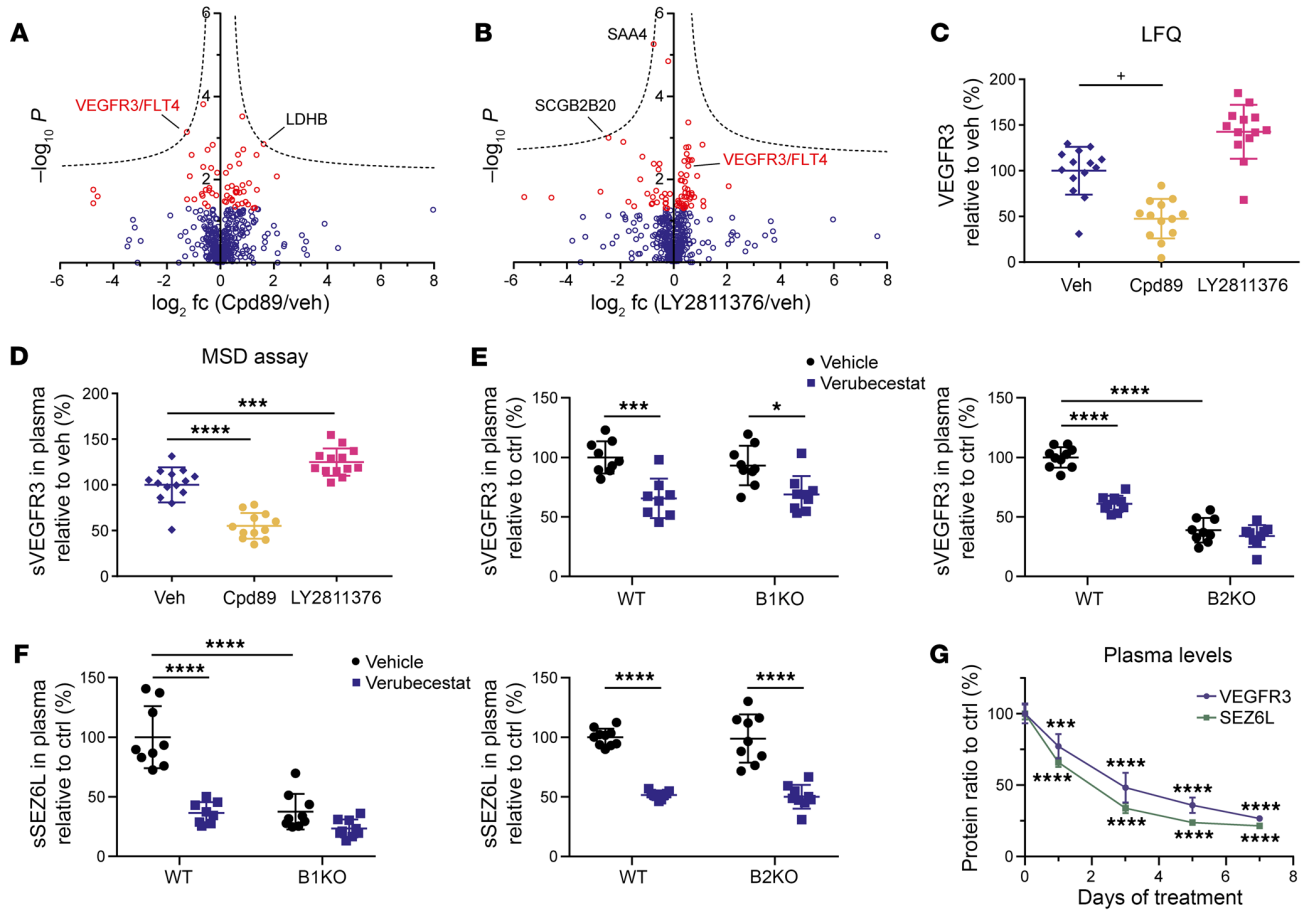
BACE2 inhibition reduces murine plasma sVEGFR3. Volcano plots of proteomic analysis of murine plasma from (**A**) compound 89–treated (Cpd89) versus vehicle-treated (veh) mice and (**B**) LY2811376-treated versus vehicle-treated mice (*n* = 13, treated; *n* = 14, veh). VEGFR3 is highlighted in red. (**C**) Corresponding extracted LFQ intensities of sVEGFR3 and (**D**) MSD-assay quantifications of sVEGFR3. Plasma sVEGFR3 (**E**) and plasma sSEZ6L (**F**) levels in 8–10 B1KO, B2KO, and respective WT mice with (blue) or without (black) 3 days of 50 mg/kg per os twice a day verubecestat dosing. (**G**) Plasma levels of VEGFR3 and SEZ6L during 7 days of 0.1% dietary verubecestat (average drug intake, 97 mg/kg/d; *n* = 6 per group, all male, age: 7–10 weeks), respective to untreated control levels. Two-sided Student’s *t* tests with a permutation-based FDR correction (FDR < 0.05; indicated by hyperbolic curves) were used for volcano plots (**A** and **B**). Proteins with *P* < 0.05 are shown as red circles. (**C**) Significance after FDR correction is indicated with plus signs. All dot plots were normalized on the control mean and depict the SD alongside the calculated *P* values, calculated by 1-way (**D** and **G**) or 2-way (**E** and **F**) ANOVA with Bonferroni’s multiple-comparison test. *P < 0.05; ***P < 0.001; ****P < 0.0001. *P* values are only indicated where significance could be observed. Number of biological replicates in **E** and **F** was 9, except for Bace1-WT + verubecestat (*n* = 8) and for Bace2WT without verubecestat (*n* = 10).

Using whole plasma proteomics, we found sVEGFR3 to be reduced by about 50% in mouse plasma after 14 days of subchronic treatment with 30 mg/kg compound 89 (Figure 6A and Supplemental Table 1), which blocks both BACE1 and BACE2 (39) (Supplemental Table 2). In contrast, 100 mg/kg LY2811376, which is a very weak BACE2 inhibitor (Supplemental Table 2 and ref. 21), did not alter sVEGFR3 in mouse plasma (Figure 6B and Supplemental Table 1). LY2811376 was previously tested in a phase 1 trial, but discontinued due to unfavorable preclinical toxicity data (21).The proteomic results (Figure 6C) were further confirmed by MSD-assay–based measurement of sVEGFR3 in the same plasma samples (Figure 6D). A reduction of sVEGFR3 in mouse plasma was also observed upon a 3-day dosing with 50 mg/kg of the clinically tested BACE1/2 inhibitor verubecestat, as measured by MSD-assay (Figure 6E). A similar verubecestat-mediated reduction was seen in BACE1-deficient mice (Figure 6E), in line with our proteomic results (Figure 1), where BACE1 deficiency had not altered sVEGFR3 abundance. BACE2-deficient mice had strongly lowered sVEGFR3 plasma abundance, and this was not further reduced with verubecestat (Figure 6E), validating that BACE2 but not BACE1 is required for sVEGFR3 release.

To demonstrate that verubecestat had also inhibited BACE1 in vivo, we developed an MSD-assay to measure the plasma concentration of murine sSEZ6L, which is a substrate of BACE1 (40), but can also be cleaved by BACE2, at least in pancreatic islets and a pancreatic cell line (13, 20). The MSD-assay is specific for mouse sSEZ6L, because it detected sSEZ6L in plasma of WT, but not of *Sez6l*-deficient, mice (Supplemental Figure 9). Plasma levels of sSEZ6L were strongly reduced upon deficiency of *Bace1*, but not *Bace2*. Similar reductions were achieved with a 3-day verubecestat treatment of WT and *Bace2*-, but not *Bace1*-deficient mice (Figure 6F), demonstrating that plasma sSEZ6L is generated by BACE1, but not by BACE2. Taken together, our results demonstrate that pharmacological inhibition of BACE1 and BACE2 in mice can be easily analyzed by measuring the changes in plasma concentration of sSEZ6L and sVEGFR3, respectively.

### Plasma sVEGFR3 shows a fast and sensitive response to changes in BACE2 activity.

The reduction of sVEGFR3 in WT mouse plasma following 3 days of verubecestat administration (61% sVEGFR3 remaining) (Figure 6E) was less pronounced compared with the sVEGFR3 reductions seen in plasma of *Bace2*-deficient mice (Figure 1, A and B, and Figure 6E), suggesting that clearance of sVEGFR3 from plasma may be relatively slow and a longer dosing period may be required to achieve maximum sVEGFR3 reduction. In fact, feeding mice with a 0.1% verubecestat-containing diet (w/w, corresponding to about 100 mg/kg/d drug intake) revealed a time-dependent sVEGFR3 reduction starting after 1 day of dosing and nearing the maximum effect size after 7 days (Figure 6G), when plasma sVEGFR3 was reduced to 27% remaining, consistent with the reduction seen in *Bace2*-deficient mice (Figure 1, A and B). A similar time-dependent reduction was also observed for plasma sSEZ6L (Figure 6G). The time course demonstrates that the plasma measurement of sVEGFR3 responds more quickly to changes in BACE2 activity compared with the fur-depigmentation assay, where changes in BACE2 activity can be read out only after 2 or more weeks (9, 16).

**Figure 7:**
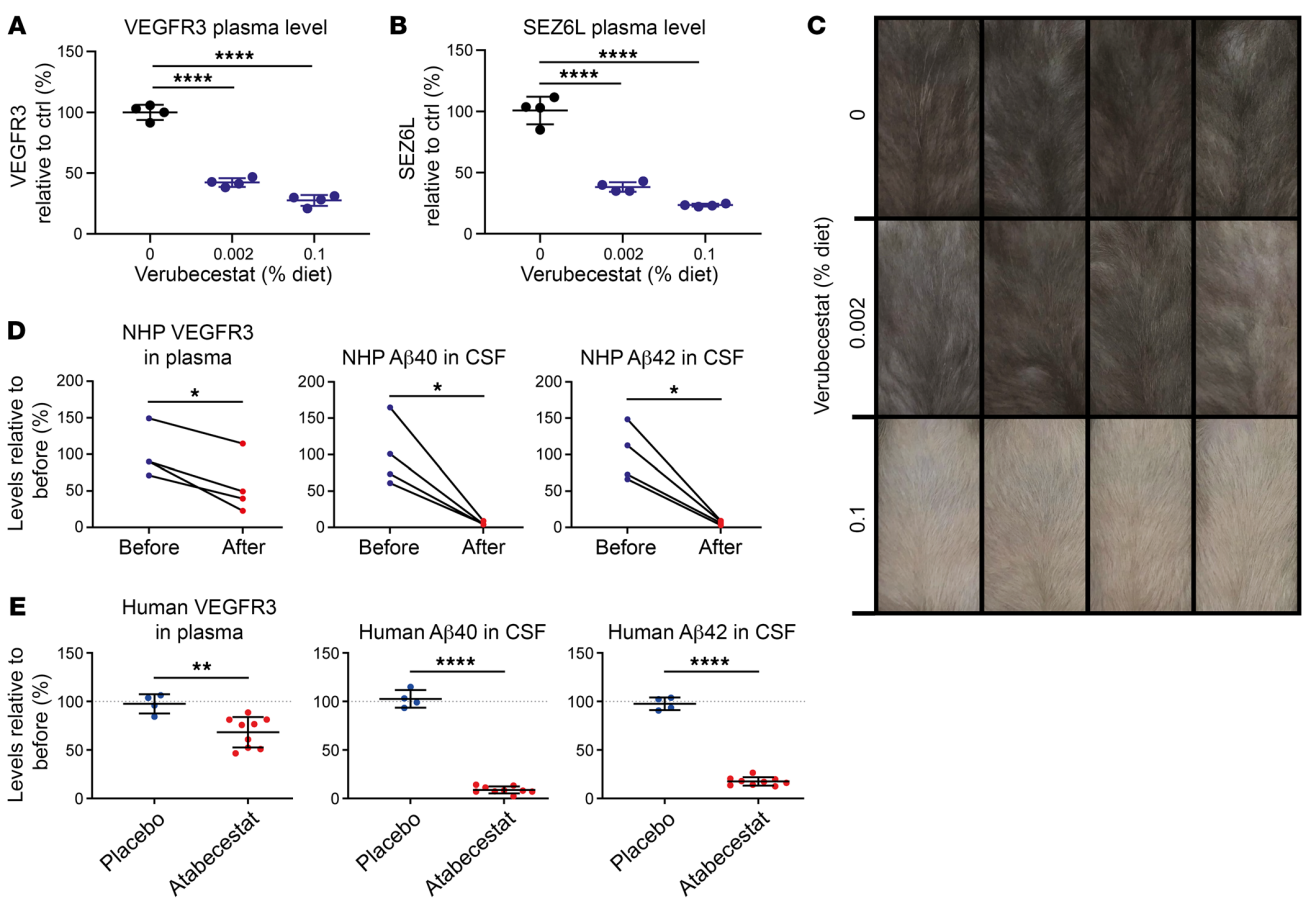
Plasma sVEGFR3 is a superior marker for BACE2 activity. (**A**) Plasma sVEGFR3 and (**B**) sSEZ6L levels in mice, fed with diet supplemented with 0 (black), 0.002%, and 0.1% of verubecestat (blue) (*n* = 4). (**C**) Photographs of the fur pigmentation of the corresponding mice. (**D** and **E**) Relative ELISA quantifications of sVEGFR3 in plasma and CSF Aβ40 as well as Aβ42 levels of (**D**) verubecestat-treated NHPs (*n* = 4) before and after treatment and (**E**) clinical trial participants treated with atabecestat (*n* = 9) or placebo (*n* = 4). Human Aβ1-40 (Aβ40) and Aβ1-42 (Aβ42) CSF data for the selected individuals were extracted from a previous publication (41). NHP data were normalized to the predose mean; human data are expressed as postdose/predose ratio for each individual. All dot plots were normalized on the control or predose mean, respectively, and (**A** and **B**) depict the SD alongside the calculated *P* values, calculated by 1-way ANOVA, followed by Bonferroni’s multiple-comparison test (**A** and **B**), paired *t* test (**D**), or unpaired *t* test (**E**). *P < 0.05; **P < 0.01; ****P < 0.0001.

To determine whether sVEGFR3 measurement is also more sensitive than fur depigmentation with regard to changes in BACE2 activity, we treated WT mice for 3 weeks with a diet containing no or 0.002% or 0.1% dietary verubecestat (approximately 0, 2, and 100 mg/kg/d drug intake, respectively). While the low verubecestat dose (0.002%) strongly lowered plasma sVEGFR3 and sSEZ6L (Figure 7, A and B), there was only a minimal effect on fur pigmentation (Figure 7C). At the high dose (0.1%), verubecestat strongly reduced plasma sVEGFR3 and sSEZ6L as well as fur pigmentation, as seen with the clearly grayed fur (Figure 7C). Thus, our benchmarking of sVEGFR3 measurement against the fur-pigmentation assay reveals that sVEGFR3 measurement is the more sensitive and faster-responding readout of changes in BACE2 activity with better pharmacodynamics and pharmacokinetics predictability.

### sVEGFR3 responds to BACE2 inhibition in plasma from nonhuman primates and humans.

Finally, we measured sVEGFR3 with an ELISA in the plasma of 4 nonhuman primates (NHP) treated for 7 days with 10 mg/kg verubecestat and in the plasma of 9 healthy human individuals treated in a phase 1 clinical trial for 28 days with doses of 50 mg of the BACE1/2 inhibitor atabecestat (41). The atabecestat dose was able to reduce CSF Aβ1-40 by up to 90% (41). From each NHP and each clinical trial participant, 2 plasma samples were available, 1 before dosing started and 1 sample after the last dosing.

In all NHPs and clinical trial participants, the BACE inhibitor reduced sVEGFR3 plasma abundance by an average of 35% in NHP and 25% in clinical trial participants (Figure 7, D and E, and Supplemental Figure 10 for time-course in NHP and for individual changes in humans), demonstrating BACE2-dependent sVEGFR3 production in primates. As a control, Aβ40 and Aβ42 in the CSF were reduced by more than 80% (Figure 7, D and E), demonstrating efficient target engagement. A similar reduction was observed for Aβ40 in the plasma of the atabecestat-treated individuals (Supplemental Figure 11). As expected, the placebo control did not alter sVEGFR3 in plasma or Aβ40 and Aβ42 in the CSF (Figure 7E and Supplemental Figure 10, B–D). The effect size of the sVEGFR3 reduction in NHP and human plasma was less compared with the maximum of achieved inhibition in mice (73%, Figure 6G). Potentially, humans produce more of the sVEGFR3 splice form or compensate the reduction of BACE2-mediated sVEGFR3 production through increased cleavage by other proteases. A similar situation is known for APP, where inhibition of its cleavage by BACE1 is accompanied by increased cleavage through ADAM10, such that total shed APP is only mildly reduced (42, 43).

In summary, sVEGFR3 can serve as an evolutionarily conserved pharmacodynamic activity marker for BACE2 in vivo.

## Discussion

Our study identifies VEGFR3 as a substrate for BACE2 in mice and humans and establishes BACE2 cleavage of VEGFR3 as a modulator of physiological VEGFR3 signaling in vitro and in vivo in zebrafish. Additionally, our study is relevant for clinical translation, as it demonstrates that soluble, BACE2-cleaved sVEGFR3 is an easily accessible pharmacodynamic marker for BACE2 activity in plasma across species.

VEGFR3 has many fundamental functions in physiology. It is required for developmental formation of blood and lymphatic vessels, for postnatal angiogenesis, and for postnatal formation and maintenance of lymphatic vessels (23, 31, 37, 44). Altered VEGFR3 expression and activity are also linked to diseases, such as Milroy disease, lymphedema, tumor-associated lymphangiogenesis, and lymphatic metastasis (45), where VEGFR3 is used as a drug target. While VEGFR3 is a transmembrane RTK, a soluble form of VEGFR3 (sVEGFR3) is naturally detected in blood and tissues (46, 47) and is tested as a biomarker for diseases, such as melanoma (26), and for monitoring chemotherapeutic treatment responses to different tumors, for example, to the drugs sunitinib and lenvatinib (48, 49). sVEGFR3 is thought to be a soluble splice form resulting from alternative splicing of the *VEGFR3* gene (46). Our study establishes a mode of sVEGFR3 generation in vitro and in vivo and reveals that (a) more than half of sVEGFR3 detected in mouse plasma derives from BACE2-mediated shedding and that (b) the amount of sVEGFR3 can be pharmacologically adjusted by inhibiting BACE2 activity. The remaining sVEGFR3 after BACE2 inactivation may represent a sVEGFR3 splice form or be generated by proteases other than BACE2. This is similar to other membrane proteins, including the AD-linked APP, that are mainly shed by one protease, but may additionally be shed by other proteases, often to a smaller degree (15).

Ectodomain shedding can regulate the function of a full-length membrane protein, but also contribute to its degradation (15). For 2 previously identified BACE2 substrates, TMEM27 and PMEL, the BACE2-mediated cleavage alters their function in mice in glucose homeostasis and pigmentation, respectively (8, 10). For other BACE2 substrates, which were mostly identified in vitro (13), it remains unclear whether their functions are altered as a result of their proteolytic cleavage. This is different for the BACE2 substrate VEGFR3, for which our study establishes BACE2 cleavage as a mechanism to control VEGFR3 signaling. BACE2 cleavage attenuates VEGFR3 signaling through reduction of the abundance of the full-length VEGFR3 available for signaling and possibly through generation of sVEGFR3, which acts as a decoy receptor attenuating VEGF-C ligand–induced VEGFR3 signaling (50, 51). Because of its decoy function, sVEGFR3 is used to therapeutically reduce VEGFR3 signaling in mice, for example, to suppress metastasis formation in a mammary cancer model (52), in lymphatic cancer models (53, 54), and for wet age-related macular degeneration and diabetic macular edema in a clinical trial (44). Beyond BACE2-mediated cleavage, the signaling function of VEGFR3 can be regulated at several levels, including through ligand processing, ligand availability, coreceptors, and receptor trafficking after internalization (44). Several proteins in the VEGFR3 signaling pathway, for example, VEGFR3 itself (31) or integrin-linked kinase (55), are essential for signaling or its control, so that their deletion results in severe or lethal phenotypes in mice. Our study demonstrates that BACE2 is not essential for VEGFR3 signaling to occur, but rather has a modulatory function, as seen with the mild facial lymphatic phenotype observed in *bace2*-deficient zebrafish. Through attenuation of VEGFR3 signaling, BACE2-mediated cleavage may represent a mechanism for fine-tuning the amount of VEGFR3 signaling. This may be particularly relevant under inflammatory conditions, which increase VEGFR3-dependent lymphangiogenesis.

Our study reveals that BACE2 inhibition increases VEGFR3 signaling in LECs and in vivo in zebrafish. This raises the possibility of considering therapeutic BACE2 inhibition for conditions with reduced VEGFR3 signaling, such as Milroy disease, which presents with lymphedema and can be caused by a heterozygous loss of VEGFR3 function (56). Yet our data in LECs suggest that acute BACE2 inhibition has a stronger effect on VEGFR3 signaling compared with chronic inhibition, indicating that the increased VEGFR3 signaling upon BACE2 inhibition may desensitize or be compensated with time.

BACE1 is a major drug target for AD. Several BACE1-targeted inhibitors have been tested in advanced clinical trials for AD, but also inhibit BACE2, partly even more potently than BACE1 (2). Most of the inhibitors induced a mild cognitive worsening, which is an unacceptable side effect that needs to be understood and prevented before BACE inhibitors may be tested in future prevention trials for AD (2). The cognitive worsening is assumed to be mechanism based and to result from too strong inhibition of either BACE1 or BACE2. Whether reduced BACE2 cleavage of VEGFR3 and enhanced VEGFR3 signaling contribute to the cognitive worsening is unknown. A clear proof is difficult or even impossible, given that cleavage products of several BACE1 substrates, including SEZ6, CHL1, APP, and NRG3, have synaptic functions (57). Hence, cognitive worsening may result from the combined inhibition of VEGFR3 and BACE1 substrates. One mechanism through which BACE2 cleavage of VEGFR3 may potentially contribute to cognitive changes involves the recently described meningeal lymphatic system, which depends on VEGFR3 activity and helps clear Aβ, tau, and other proteins from the central nervous system (58, 59). Notably, decreased CSF clearance is associated with enhanced cognitive decline (60).

For BACE1, several substrate cleavage products, such as Aβ and sSEZ6, are detectable in CSF and partly in blood and can be used to measure target engagement of BACE1 (16, 20). In contrast, for BACE2, target engagement in humans and rodents cannot be routinely assayed because of the lack of substrate cleavage products detectable in body fluids. As a work-around, in rodents, BACE2 inhibition is currently functionally monitored by qualitative scoring of hair depigmentation upon chronic BACE2 inhibition over several weeks (9, 16), which precludes detailed pharmacodynamic analyses. Our study demonstrates that sVEGFR3 may serve as a pharmacodynamic BACE2 activity marker. sVEGFR3 is easily measurable using an MSD-assay, both in rodents and humans, and is superior to the chest hair depigmentation assay, both in terms of sensitivity and rapidness. Importantly, sVEGFR3 can be measured in blood, which is more easily accessible than CSF. Because BACE2 inhibition only moderately reduced sVEGFR3 plasma concentration, the routine use of sVEGFR3 as a pharmacodynamic marker for BACE2 activity requires further optimization, which may comprise the generation of antibodies that specifically detect the neo–C-terminus of the BACE2-cleaved sVEGFR3 and thus do not crossreact with the sVEGFR3 splice variant or sVEGFR3 species generated by other proteases.

We envisage numerous translational opportunities for measuring BACE2 cleavage–specific plasma sVEGFR3. Applications include the use of sVEGFR3 as a BACE2 marker in conditions characterized by altered sVEGFR3 levels, e.g., preeclampsia, hypertension in systemic sclerosis, or treatment-induced changes in sVEGFR3 in VEGFR3-dependent diseases, such as sunitinib-treated cancers (26, 48, 49). In all of these conditions, it remains unclear whether the change in sVEGFR3 results from altered VEGFR3 cleavage by BACE2 or other proteases or from different abundance of the sVEGFR3 splice form. Thus, measurement of the BACE2-specific sVEGFR3 changes holds the potential of providing mechanistic insights into disease pathogenesis and treatment responses. An additional important use for measuring BACE2 cleavage–specific plasma sVEGFR3 is the development of a new generation of drugs specifically targeting BACE1, but not BACE2, for safer treatment of AD and, conversely, the development of BACE2-selective inhibitors (avoiding BACE1 inhibition) for potential treatment and measuring treatment responses in melanoma, glioblastoma, and diabetes, where inhibition of BACE2 is considered as a therapeutic approach (8, 12). Development of BACE1- versus BACE2-specific drugs requires pharmacodynamic markers to measure BACE1 and BACE2 activity in vivo. We propose to use sVEGFR3 and sSEZ6L as easily accessible markers for BACE2 and BACE1 activity, respectively, when analyzing plasma. The SEZ6L homolog SEZ6 is an additional established BACE1 substrate (20, 40), but is predominantly expressed in neurons and was not found in mouse plasma in our proteomic studies, presumably because of its absence from or low abundance in plasma. Yet sSEZ6L and sSEZ6 are also suitable markers for BACE1 activity in brain and can be measured in CSF (20, 61), whereas for BACE2, no suitable and sensitive marker has yet been identified in CSF (6). For AD, the development of BACE1-selective inhibitors has a dual benefit. First, it may prevent the cognitive side effect of the BACE inhibitors if the side effect results from inhibition of cleavage of VEGFR3 or other BACE2 substrates. Second, BACE2 has a protective activity with regard to AD pathology as shown in disease models of trisomy 21 (62) and Hirschsprung disease (63) and seen with the finding that polymorphisms in the BACE2 gene correlate with the age of onset of AD in trisomy 21 (64). The protective effect results because of BACE2’s ability to cleave within the pathogenic Aβ peptide sequence after amino acids 19, 20, and 34 (14, 62). Thus, BACE2 may prevent Aβ formation and contribute to Aβ degradation. Consequently, sparing BACE2 would be beneficial for new BACE1-targeted inhibitors developed for AD.

Our study, identifying a function of BACE2 in cleavage and signaling modulation of VEGFR3, has direct implications for the basic physiology of BACE2 and VEGFR3 and opens translational opportunities for diseases dependent on BACE2 or VEGFR3.

## Methods

### Sex as a biological variable

Mixed sex groups were used for most experiments, but sex was not considered as a biological variable. Details for the individual experiments are provided in the Supplemental Methods.

### Animal work

The animal work is described in detail in the Supplemental Methods.

### Identification of VEGFR3 as a BACE2 substrate

#### Digestion of nonenriched plasma.

For Figure 1, A and B, 1 μL plasma per sample was reduced, alkylated, and digested in 0.1% sodium deoxycholate (SDC) with 3 μg LysC and 3 μg trypsin at room temperature overnight. SDC precipitate was removed after acidification with formic acid (FA) by centrifugation at 16,000 *g*, and samples were stage tipped (65) before mass spectrometric measurements.

#### Digestion of glycoprotein-enriched plasma.

Plasma (10 μL) of the different genotypes was subjected to glycoprotein capturing with hydrazide resin to enrich glycoproteins as previously described (66) followed by an on-bead tryptic digestion with some modifications (for details, see Supplemental Methods).

#### LC-MS/MS analysis.

All murine plasma samples were analyzed on an EASY nLC-1000 nano UHPLC (Thermo Fisher Scientific) coupled online via a Nanospray Flex electrospray ion source equipped with a column oven (Sonation) to a Q-Exactive HF mass spectrometer (Thermo Fisher Scientific). An amount of 1 to 1.3 μg of peptides were separated on self-packed C18 columns (500 mm × 75 μm, ReproSil-Pur 120 C18-AQ, 1.9 μm; Dr. Maisch, High Performance LC GmbH) using a binary 120 minutes (Figure 1, A and B) or 180 minutes (Figure 1, E–I) gradient of water (a) and 100% acetonitrile (b) supplemented with 0.1% FA. A top 20 data-dependent acquisition (DDA) method was used for spectral library generation. Nonenriched plasma samples were measured with a 120-minute gradient in data-independent acquisition mode (Supplemental Table 3). Glycoprotein-enriched plasma samples were analyzed in DDA mode with a 180-minute gradient. Details are provided in Supplemental Methods.

#### MS data analysis.

All DDA data were analyzed via Maxquant (version 1.5.5.1.) software (67) for label-free protein quantification, using default settings with slight modifications (see Supplemental Methods). Glycoprotein-enriched samples were searched against a reviewed canonical database of *Mus musculus* from Uniprot (download: 2017-01-11, entries: 16,843 proteins). For the spectral library generation, 4 WT and 4 *Bace2*-knockout (B2KO) mouse plasma samples, supplemented with the Biognosys iRT kit, were searched against a reviewed isoform database of *Mus musculus* from Uniprot (download: 2017-04-11, entries: 24,992 proteins) using Maxquant and results were loaded into the Biognosys software Spectronaut (version 11.0.15038.22.23735) (68).

Nonenriched DIA plasma samples were analyzed with the self-generated spectral library, using the default settings with Spectronaut. Protein label-free quantitation (LFQ) was performed on the MS1 level and required at least 1 identified peptide.

Changes in protein abundance were evaluated using a Student’s *t* test between the log_2_ transformed LFQ intensities of the experimental groups. A permutation-based FDR estimation was used to account for multiple hypotheses (*P* = 5%; s0 = 0.1) using the software Perseus, version 1.6.2.3 (69). Volcanos only display proteins, which were identified in at least 3 replicates of the related experimental groups.

#### MSD electrochemiluminescence detection of murine plasma VEGFR3.

For quantification of murine plasma VEGFR3, MSD immunoassay plates (L15XA-3, MSD) were coated with VEGF receptor 3 monoclonal antibody AFL4 (14-5988-82, Thermo Fisher), blocked with casein, and incubated with heat-denatured samples. VEGFR3/Flt-4 biotinylated antibody (BAF743, R&D Systems) was used as secondary antibody. For detection, SULFO-TAG–labeled streptavidin (R32AD-1, MSD) was used at the recommended dilution of 1:500 and 2× diluted 4× Read Buffer T (R92TC-1, MSD). Details are provided in Supplemental Methods.

#### Immunological Western blot detection.

Prior to immunoblot analysis, plasma samples were depleted from immunoglobulins using protein A, G, and L magnetic beads (88846, 88848, 88850, Thermo Scientific) followed by glycoprotein enrichment using concanavalin A agarose conjugate (C7555, MilliporeSigma). Proteins were separated on 8% polyacrylamide gels and transferred to nitrocellulose membranes using the Bio-Rad Trans-Blot Turbo system. Membranes were blocked with 0.1% casein, incubated with primary antibody (BAF743, R&D Systems), and washed 3 times followed by incubation with the secondary antibody and 3 washing steps. Immunoblots were developed using ECL Western Blotting Reagents. Images were generated using the ImageQuant LAS 4000 platform. Displayed images were cropped using Photoshop, version 12.1. Details are provided in Supplemental Methods.

### Cleavage characterization of BACE2 for VEGFR3

#### HEK293 overexpression.

HEK293 cells (ATCC) were transfected with *Bace2* and *Vegfr3* plasmids (ratio 1:3) using lipofectamine 2000 (11668019, Thermo Fisher), murine full-length *Bace2* in pcDNA3.1 (provided by Hyeryun Choe, Harvard Medical School, Boston, Massachusetts, USA; ref. 14), murine full-length 2xHa-*Vegfr3*-2xFlag in pFUGW (Genescript), and corresponding empty backbones (14883, Addgene). Details are provided in Supplemental Methods.

#### Collection of cell supernatant media and lysates.

Medium was collected, supplemented with protease inhibitor (Pi; P8340, MilliporeSigma), and centrifuged at 4°C for 10 minutes at 15,300 *g* to remove cell debris. Cells were washed with ice-cold PBS and lysed on ice for 10 minutes in 150 μL of STET lysis buffer with 2 μL/mL Pi. Deglycosylated samples were generated according to the manufacturer’s protocol, using PNGase F (P0704, New England Biolabs). Details are provided in Supplemental Methods.

#### Immunological Western blot detection.

Murine VEGFR3 was detected as described above. Anti–HA-7 (H9658, MilliporeSigma) and anti–FLAG M2 (F1804, MilliporeSigma) were used for detection of the utilized tags. Anti β-actin (A5316, MilliporeSigma) and anti-BACE2 (ab5670, Abcam) were used for the detection of actin and BACE2.

#### Enzymatic and cellular BACE activity determination.

BACE1 and BACE2 enzymatic activity was assessed via a fluorescence resonance energy transfer (FRET) assay as described previously (20). Cellular BACE1 activity was determined as described previously (20). Cellular BACE2 activity was determined using mouse insulinoma 6 (MIN6; C0018008, AddexBio) cells expressing murine *Vegfr3*. BACE inhibitors were added to the cells and incubated for 18 hours; media were collected for subsequent VEGFR3–MSD-assay analysis. Details are provided in Supplemental Methods.

#### VEGFR3 purification for cleavage site determination.

MIN6 cells stably expressing murine *Vegfr3* were used, and conditioned media were immunoprecipitated using the Dynabead Protein Immunoprecipitation Kit (10007D, Thermo Scientific) and 20 μL AFL4 antibody per 50 μL beads. Eluted VEGFR3 was deglycosylated, using the Deglycosylation Kit (P6044, New England Biolabs). Details are provided in Supplemental Methods.

#### Sample preparation for cleavage-site determination.

Proteolytic digestion was performed using a modified protocol for single-pot solid-phase enhanced sample preparation (SP3) (70). Samples were digested using LysN (90301, Thermo Fisher). Details are provided in Supplemental Methods.

#### LC-MS/MS and data analysis for cleavage-site determination.

SP3 digested samples were separated on self-packed C18 columns using a 60-minute gradient and analyzed on a Q-Exactive HF mass spectrometer using a top 10 DDA method. The identified cleavage site–derived peptide was validated using a synthetic peptide with the same sequence including its modifications (cysteine carbamidomethylation, free COOH group, N→D, Peps4Life), which was analyzed with the same method. Details are provided in Supplemental Methods.

### Endogenous VEGFR3 cleavage in LECs

#### Immunological detection in LECs.

Human microvascular endothelial cells (HMVEC-dlyAd; CC-2543, Lonza), referred to as LECs, were cultured in EBM supplemented with EBM Growth Kit (CC-3202, Lonza); 200,000 cells per 6 wells were used, and 10 μL of 5 μM siRNA smart pools (003747-00, 003802-00, 001810-10, Horizon) were used for *BACE* knockdown. For pharmacological experiments, cells were treated with DMSO, 2 μM β-secretase inhibitor IV (565788, Merck), or 100 nM verubecestat (MBS579527, BIOZOL) for 24 hours. LECs were lysed using 100 μL of STET. Immunological Western blot detection was performed as described, using the anti-human VEGFR3 antibody (MAB3757, Chemicon), the same BACE2 antibody, and additionally, anti-BACE1 (5606S, Cell Signaling Technology) for detection of BACE1. Densitometric quantifications were performed on the resulting images using the software Fiji ImageJ (2.0.0-rc-67/1.52c). Details are provided in Supplemental Methods.

#### Gene expression analysis in LECs.

250,000 Cells were seeded in each well of a 6-well plate and grown for 24 hours in 1600 μL EBM. For pharmacological inhibition, cells were serum starved overnight. The next morning, medium was replaced with fresh EBM supplemented with DMSO, 100 nM verubecestat, or 1.5 μg/mL VEGF-C156S (752-VC, R&D Systems) and incubated for 100 minutes.

Transfection for *BACE* knockdown was performed as indicated above, and after 24 hours of incubation, cells were serum starved overnight via the exchange of serum-free EBM. The next morning, medium was replaced with fresh EBM, supplemented with DMSO, 100 nM verubecestat, or 1.5 μg/mL VEGF-C156S, and incubated for 100 minutes. Cells were collected and RNA was purified using the QIAGEN RNA Preparation Kit (74104, QIAGEN) and RNA was transcribed into cDNA using iScript Reverse Transcription Mix (1708841, Bio-Rad) according to the manufacturer’s protocol. For the qPCR reaction, SYBR Green (1725270, Bio-Rad) Master Mix (10 μL SYBR Green Buffer, 2 μL SYBR Green Primer, and 8 μL cDNA) was made for each gene (qHsaCID0012647, qHsaCID0012156, qHsaCED0044238, qHsaCID0020886, qHsaCED0047198, qHsaCEP0041396; Bio-Rad). Samples were measured in duplicate using default comparative Ct methods and the default 2-hour ramp speed of the StepOnePlus Thermocycler (Thermo Scientific). Primer efficiencies were considered for relative gene expressions, and *GAPDH* was used as a homebox gene. Details are provided in Supplemental Methods.

### BACE2-dependent VEGFR3 function in zebrafish

#### Imaging of zebrafish facial lymphatics.

Imaging of facial lymphatics followed the protocol as described previously (36). In brief, embryos were subjected to fluorescence imaging at 3 dpf and 5 dpf following Tricaine (MS-222) treatment and embedding in 0.8% low melting agarose containing MS222. Fluorescence images were captured on a Leica SP8 inverted microscope. Images were processed and analyzed using Fiji ImageJ. Statistical analysis was performed using Graphpad Prism 9. Brightness and contrast were adjusted for improved visualization.

### Pharmacodynamic properties of VEGFR3

#### Proteomic analysis.

Murine plasma proteomics was performed as described above for undepleted plasma.

#### MSD electrochemiluminescence detection of plasma VEGFR3 and SEZ6L.

Murine VEGFR3 MSD assay was performed as described above. For murine SEZ6L MSD assay, plates were coated using 3 μg/μL sheep anti-mouse SEZ6L antibody (AF4804, R&D Systems). The primary and secondary detection antibodies were an in-house–generated rat monoclonal anti-SEZ6L antibody (clone 21A11, IgG-2a) and SULFO-TAG–labeled goat anti-rat (R32AH-1, MSD). Plasma was diluted 1:1 in blocking buffer, but was not heat denatured. An 11-point, half-log SEZ6L standard curve (3000–0.3 pM) was generated by serial dilution of recombinant mouse SEZ6L (4804-S6, R&D Systems) in blocking buffer.

For human VEGFR3 MSD-assays VEGFR3 was detected using goat polyclonal anti-human VEGFR3/Flt-4 biotinylated antibody (BAF349, R&D Systems). Details are provided in Supplemental Methods.

NHP (*Macaca fascicularis*, female, 7–8 years of age, *n* = 4) sVEGFR3 was quantified using a commercial human ELISA kit (27779, Immuno-Biological Laboratories), according to the manufacturer’s protocol.

#### Human plasma sVEGFR3 measurements.

Human plasma samples for VEGFR3 measurement were obtained from elderly White patients (aged 50–90 years) who had been diagnosed as having preclinical AD or as having mild cognitive impairment (MCI) due to AD and were enrolled in a 28-day trial designed to evaluate the safety, pharmacokinetics, and pharmacodynamics of the nonselective BACE inhibitor atabecestat (Study ALZ1005; ClinicalTrials.gov NCT01978548) (41). Samples collected at baseline and day 28 from 5 randomly selected patients that received a 50 mg daily oral dose of atabecestat in study ALZ1005 were analyzed for sVEGFR3 levels as described above.

### NHP CSF Aβ measurement

The CSF samples for Aβ measurement were obtained 8 hours after last administration from 4 NHPs treated for 7 days with 10 mg/kg verubecestat. Aβ40 was quantified using a Human β Amyloid (aa 1–40) ELISA Kit (Wako) and Aβ42 was quantified using a Human β Amyloid (aa 1–42) ELISA High Sensitive Kit (WAKO) according to the manufacturer’s protocols.

### Statistics

Statistical analysis was done using GraphPad Prism, version 9.5.1, Microsoft Excel 2019, and Perseus software, version 1.6.2.3. Details are provided in the figure legends. Analyses were performed using the 2-tailed, unpaired *t* test and 1-way or 2-way ANOVA with Bonferroni’s multiple-comparison test. When multiple *t* tests were applied, either *P* values were corrected by Bonferroni’s multiple-comparison test or *t* tests were combined with permutation-based FDR correction.

### Study approval

All murine animal procedures were carried out in accordance with the European Communities Council Directive (86/609/EEC), and onsite procedures were approved by the committee responsible for animal ethics of the government of Upper Bavaria (no. 02-19-067) and Belgium (no. LA1100119). Zebrafish experiments were performed in accordance with animal protection standards of the Ludwig-Maximilians University Munich and were approved by the government of Upper Bavaria (Regierung von Oberbayern, Munich, Germany) or the Animal Ethics Committee at the University of Münster. Fish maintenance was in accordance with Federation of European Laboratory Animal Science Associations (FELASA) guidelines (71). All NHP experiments and sampling were conducted by Shionogi & Co. and performed according to AAALAC and the Shionogi Ethics Committee.

### Data availability

The mass spectrometry proteomics data have been deposited to the ProteomeXchange Consortium via the PRIDE partner repository (72) with the data set identifiers PXD041577, PXD041579, and PXD042669. Requests for clinical materials may require an MTA. Values for all data points in graphs are reported in the Supporting Data Values file.

## Author contributions

SFL designed the study with help from DM, BH, and SAM and AS. AS performed all mass spectrometric experiments with the help of SAM and respective immunoblot validation in the different mouse models. AS, together with XF and with the support of LIH and MA, carried out all experiments related to LEC and HEK cells. LSH and EL contributed to the design of LEC signaling experiments. BH set up MSD-assays for sVEGFR3 and performed additional immunoblot validations of murine and human sVEGFR3. BH and ELS handled MIN6 cell culture–related work and generation of purified sVEGFR3. Mouse experiments were mostly conducted by BH, with help from IV and BDS. Verubecestat treatments in zebrafish were carried out by SSK and SSM or by FVB with support from BS, GJ, and MA. NHP experiments and the corresponding ELISA were performed by MI, KH, AY, and NH. MP set up the sSEZ6L MSD-assay with help from RF. Data analysis and interpretation were done by AS, BH, SAM, DM, SKT, and SFL. The manuscript was written by SFL with input from all authors. The order of the two first authors’ names was determined by joint decision of both authors and their supervisors.

## Supplementary Material

Supplemental data

Unedited blot and gel images

Supplemental table 1

Supporting data values

## Version 1 06/18/2024

In-Press Preview

## Version 2 08/15/2024

Electronic publication
