# Supplementary material for: The Alzheimer’s disease–linked protease BACE2 cleaves VEGFR3 and modulates its signaling
Source: J Clin Invest. 2024 Jun 18;134(16):e170550. doi: 10.1172/JCI170550 (PMC11324312; doi:10.1172/JCI170550)
Supplement: Supplemental data [file jci-134-170550-s092.pdf]

Supplementary Materials and Methods

Animal work

Mice

Mice were maintained on a 12:12h light:dark reverse light cycle under standard vivarium conditions (22 ± 2 ºC and 55 ± 10 RH). Plasma and/or tissues were collected under Ketamine/Domitor or isoflurane anesthesia 4 h after last dosing (in case of PO dosing) or after light onset (in case of medicated diets). Sex was not considered as a biological variable. For most experiments, both male and female animals were used. Mice used for the proteomic experiments (Figure 1) included: *Bace1* tm KO (1), C57BL/6-*Bace2*<tm1.2>$^{-/-}$ (RIKEN Brain Science Institute), and BDKO mice produced by breeding the aforementioned *Bace1* tm KO mice and *Bace2* ΔE6$^{-/-}$ mice, which harbor a deletion of exon 6 of Bace2 that contains the active site of the enzyme (Dominguez et al, 2005). The RIKEN *Bace2*<tm1.2>−/− mice used in this study lack the BACE2 protein completely and were imported from the RIKEN BRC through the National Bio-Resource Project of the MEXT/AMED, Japan. For pharmacology experiments (Figure 6) *Bace2* ΔE6-/- mice were used (1).

Figure 1 A-D: *Bace2* KO mice (B2KO) with C57BL/6JRj background (C57BL/6-*Bace2*<tm1.2>$^{+/+}$ and $^{-/-}$; n=6; female; age: 18-19 weeks). Mice were imported from the RIKEN BRC through the National Bio-Resource Project of the MEXT/AMED, Japan.

Figure 1 E-I: WT (C57BL/6JRj, 2 male, 7 female); B1KO (*Bace1* tm KO $^{-/-}$, 3 male, 6 female); B2KO (*Bace2* ΔE6 KO$^{-/-}$;, 7 male, 2 female) (2); BACE1 WT (*Bace1* tm KO $^{+/+}$, 6 male, 3 female); BACE1/2 double KO / BDKO (Bace1 tm KO $^{-/-}$, *Bace2* ΔE6 KO$^{-/-}$; 2 male; 7 female) age: 16-21 weeks.

Figure 6 A-D: pharmacological BACE inhibition, 3 to 4-month-old male C57BL/6JRj mice (N = 14/group) were dosed for 14 days at 0, 30 or 100 mg/kg PO b.i.d. with vehicle (0.5% methylcellulose/0.1%Tween 80), Compound 89 or LY2811376 respectively in a volume of 10 ml/kg.

Figure 6 E-F: B1KO (*Bace1* tm KO $^{-/-}$; 4 male and 5 female per treatment group, age: 8-12 months); B2KO (*Bace2* ΔE6 KO$^{-/-}$;; 9-10 males per treatment group, age: 3-6 months), and WT (C57BL/6JRj, Janvier, France, 8-9 males per treatment group; age: 3-4 months) were assigned to vehicle (20% cyclodextrin) or verubecestat (3 days at 50 mg/kg PO b.i.d. in volume of 10 ml/kg at 12 h intervals) treatment.

Figure 6 G: C57BL/6JRj (n=6 per group; all male; age: 7-10 weeks) were treated with verubecestat (50 mg/kg PO b.i.d. in volume of 10 ml/kg at 12 h intervals) for 0, 1, 3, 5, and 7 days.

Figure 7 A-B: To assess the impact of verubecestat treatment on BACE substrate levels (time course) and fur depigmentation (21-day treatment), verubecestat was administered in the diet of C57BL/6JRj mice (Janvier, France, n=4 per group; all male; age: 7-10 weeks). For this, the AIN-93G rodent diet (Bio Services BV, Uden NL) was used as the experimental diet to which drug was incorporated. AIN-93G diet was initially formulated with 2% w/w soybean oil. To produce the medicated diet, verubecestat was dissolved in and aliquot of soybean oil by stirring for 48 h to produce a white milky mixture in which no particulates could be observed. The drug-oil mixture was mixed with the base diet mix to produce a diet with a final concentration of 15% soybean oil (w/w). For fur depigmentation, mice were depilated using Veet facial wax under isoflurane anesthesia prior to verubecestat treatment. Newly grown hair was assessed after 21 days. Pictures were taken under same light conditions, using a customary camera.

Supplementary Figure 5: *Sez6l*$^{-/-}$ and C57BL/6JRj (n=3, all male) (3, 4).

Most *in vivo* mouse material (except for Sez6l$^{-/-}$ material, sampled at DZNE) was sampled at the respective sites of Janssen Pharmaceutica and Shionogi & Co., Ltd: Samples were sent to the DZNE for processing and analysis.

Zebrafish

Zebrafish husbandry and experiments were performed in accordance with animal welfare guidelines and good scientific practices as recommended by (5). Either the wildtype strain AB was used, or the transgenic lines listed below. No inbred lines were used. The age of the parental fish was not monitored. All embryos were kept at 28.5°C in E3 media (5 mM NaCl, 0.17 mM KCl, 0.33 mM $CaCl_2$, and 0.33 mM $MgSO_4$) and were staged according to (6).

The transgenic line *Tg(flt4:mCitrine)^hu7135^*, which drives expression of mCitrine under the control of the Fms-related tyrosine kinase 4 promoter (*flt4*) (7, 8) was used for analysis of venous and lymphatic endothelial cells. The transgenic line *Tg(lyve1:dsRed2)^nz101^*, which drives expression of dsRed under the control of the *lyve1* promoter (9), was also used for analysis of lymphatic endothelial cells as well as the $Tg(fli1a:nEGFP)_{y7}$ line which expresses nuclear GFP (10). For verubecestat experiments, embryos were collected within a 30 min time window from mass matings, and exposed to vehicle or 100 µM verubecestat from 1 hour post fertilization onwards.

The double transgenic line *Tg(claudin k:Gal4)*; *Tg(14xUAS:GFP)* that drives membrane-bound GFP expression under control of the *claudin k* promoter (11) was used for analysis of myelination and is referred to as *claudin k:GFP* throughout the manuscript.

Identification of VEGFR3 as a BACE2 substrate

Digestion of non-enriched plasma

For Figure 1A and B, plasma was diluted 1:10 in 50 mM ammonium bicarbonate (ABC) and 10 µl of the dilution were combined with 10 µl denaturation buffer (0.5% SDC in 250 mM ABC). 1 µl of 10 mM DTT was added and the mixture incubated at room temperature (RT) for 20 min. 1 µl of 55 mM iodoacetamide (IAA) was added for alkylation and incubated at RT for 30 min. After another 20 min incubation step with 1 µl of 10 mM dithiothreitol (DTT) at RT, 3 µg of LysC (90307, Thermo Scientific) in 50 mM ABC was added and incubated at RT for 3 h. Next, 3 µg trypsin (90057, Thermo Scientific) in 50 mM ABC was added and incubated at RT overnight. Digested peptides were acidified with 150 µl 0.1% formic acid (FA) and 10 µl 8% FA and SDC precipitated at 4°C for 20 min. Precipitate was removed by centrifuging at 4°C for 10 min and 20,000 g. Samples were stage-tipped (12) before mass spectrometric measurements.

Digestion of glycoprotein-enriched plasma

Plasma (10 µL) of the different genotypes was subjected to glycoprotein capturing to enrich for glycoproteins. Briefly, plasma was diluted ten-fold using glycolink coupling buffer (Thermo Scientific) and glycans were oxidized using sodium periodate (final concentration: 1mM) for 45 min at RT in the dark. Samples were desalted using Zeba Spin Desalting Columns (Thermo Scientific). Oxidized glycoproteins were covalently coupled to Ultralink hydrazide resin (Thermo Scientific) using 0.2 M aniline as a catalyzer and incubation on an end-over-end mixer at room temperature for 4 h. Afterwards, beads were washed on snapcap columns (Thermo Scientific) with 400 µL 1% SDS (4×), 1M NaCl (4×), and 8M urea (4×) each in 100 mM TrisHCl pH 8.5. Beads were transferred to 1.5 mL protein Lobind tubes. Protein disulfide bonds were reduced using 10 mM dithiothreitol and free cysteines were alkylated in 55 mM iodoacetamide. The resin was washed twice with 200 µL of 50 mM ammoniumbicarbonate. Finally, proteins were digested using 0.15 µg of LysC (Promega) for 3 h at 37°C and 0.15 µg of trypsin (Promega) for 16 h at 37°C. The resin was removed by centrifugation and the peptides in the supernatant were purified using C18 STAGE tips. Eluted peptides were concentrated by vacuum centrifugation and dissolved in 0.1% formic acid for mass spectrometric analysis.

LC-MS/MS analysis

All murine plasma samples were analyzed on an Easy nLC-1000 nano UHPLC coupled online via a Nanospray Flex electrospray ion source equipped with a column oven (Sonation, Germany) to a Q-Exactive HF mass spectrometer. An amount of 1-1.3 µg of peptides were separated on self-packed C18 columns (500 mm × 75 µm, ReproSil-Pur 120 C18-AQ, 1.9 µm; Dr. Maisch, Germany) using a binary 120 min (Figure 01 A-B) or 180 min (Figure 01 E-I) gradient of water (A) and 100% acetonitrile (B) supplemented with 0.1% FA (120min: 0 min, 3% B; 2 min 6% B; 92 min, 30% B; 112 min, 44% B; 120, 75%, 182 min: 0 min, 2% B; 3.5 min 5% B; 137.5 min, 25% B; 168.5 min, 35% B; 180 min , 60% B).

Spectra for data independent acquisition (DIA) library generation were acquired at a resolution of 120,000 (AGC target 3E+6). The 20 most intense peptide ions were chosen for fragmentation by higher energy collisional dissociation (resolution 15,000, maximum ion trapping time: 50 ms, isolation width: 1.6 m/z, AGC target 1e+5, NCE:26%). A dynamic exclusion of 180 s was applied for fragment ion spectra acquisition.

Non-enriched plasma samples were measured with a 120 min gradient in data independent acquisition mode. Full MS spectra were acquired at a resolution of 120,000 (AGC target 5E+6). DIA fragmentation

spectra were acquired by higher-energy collisional dissociation of all ions in 20 windows of variable size (resolution: 30,000, AGC target: 3E+6, stepped NCE 23.4%, 26%, 28.6%).

DIA windows for murine plasma. Optimized m/z window distribution for Sequential Window Acquisition of All Theoretical Mass Spectra (Swath-MS) based data independent acquisition.

Window	m/z start	m/z end	Center	Isolation width [m/z]
1	300	385	342.5	85
2	384	424	404	40
3	423	453	438	30
4	452	480	466	28
5	479	505	492	26
6	504	529	516.5	25
7	528	552	540	24
8	551	575	563	24
9	574	598	586	24
10	597	621	609	24
11	620	645	632.5	25
12	644	671	657.5	27
13	670	698	684	28
14	697	726	711.5	29
15	725	759	742	34
16	258	796	777	38
17	795	841	818	46
18	840	901	870.5	61
19	900	1051	975.5	151
20	150	1402	1226	352

Glycoprotein enriched plasma samples were analyzed in data depended acquisition mode with a 180 min gradient. Full MS spectra were acquired at a resolution of 60,000 (AGC target: 3E+6). The 15 most intense peptide ions were chosen for fragmentation by higher-energy collisional dissociation (resolution: 15,000, maximum ion trapping time: 50 ms, isolation width: 2 m/z, AGC target: 3E+3, NCE: 25%). A dynamic exclusion of 120 s was applied for fragment ion spectra acquisition.

MS data analysis

All DDA data were analyzed via the Maxquant (1.5.5.1.) software (13), using the default setting with slight modifications as follows. Trypsin/P was defined as protease and the minimal peptide length was set to six amino acids. Two missed cleavages were allowed. The option first search was used to recalibrate the peptide masses within a search window of 20 ppm. For the main search, the peptide mass tolerance was set to 4.5 ppm. Tolerances for peptide fragment ions were set to 20 ppm for HCD. Carbamidomethylation of cysteine was defined as static modification. Acetylation of N-termini as well as oxidation of methionines were set as variable modifications. The false discovery rate for both peptides and proteins was adjusted to less than 1% using a target and decoy approach (concatenated forward and reversed database). Label free quantification (LFQ) of proteins required at least two ratio counts of razor peptides. Only unique peptides were used for quantification. The option “match between runs” was enabled with a matching time of 1.5 min.

For the spectral library generation, four WT and four B2KO mouse plasma samples, supplemented with the Biognosys iRT kit, were searched against a reviewed isoform database of Mus Musculus from Uniprot (download: 2017-04-11, entries: 24,992 proteins) using Maxquant. Match between runs, label free quantification and contaminant inclusion were disabled. Generated results were loaded into the Biognosys software Spectronaut (11.0.15038.22.23735) (14) and a library was generated using the default settings.

Using Spectronaut, the non-enriched DIA plasma samples, supplemented with the Biognosys iRT kit, were matched against the self-generated library, using the default settings. Briefly, a 1% FDR was applied to peptide and protein identifications and LFQ was performed on the MS1 level. LFQ of proteins required at least one identified peptide and was done with up to three peptides. For the glycoprotein enrichment samples, spectra were searched against a reviewed canonical database of Mus Musculus from Uniprot (download: 2017-01-11, entries: 16,843 proteins). Changes in protein

abundance were evaluated using a Student's t-test between the log2 transformed LFQ intensities of the experimental groups. A permutation-based FDR estimation was used to account for multiple hypotheses ($p = 5\%$; $s0 = 0.1$) using the software Perseus (15). Volcanos only display proteins, which were identified in at least 3 replicates of the related experimental groups.

Meso Scale Discovery (MSD) electrochemiluminescence detection of murine plasma VEGFR3
For quantification of murine plasma VEGFR3, MSD immunoassay plates (L15XA-3) were coated overnight with 30 µl of 3 µg/ml rat VEGF Receptor 3 Monoclonal Antibody AFL4 (Catalog No. 14-5988-82, ThermoFisher) and blocked for 2 h in 0.1% casein in PBS, (Blocker™ Casein, Catalog No. 37528, ThermoFisher) before adding samples. Mouse plasma samples were diluted 1:8 in blocking buffer, heated at 70ºC for 10 min while shaking at 700 RPM, cooled on ice and centrifuged briefly before loading on the blocked MSD plate. The heat denaturation step was critical for detection of VEGFR3 in plasma samples. An 11-point, half-log standard curve (3000 – 0.3 pM) was generated in blocking buffer using rmVEGFR3/Flt-4 Fc Chimera Protein (Catalog No. 743-R3, R&D Systems) and heat denatured like plasma. Subsequently 50 µl sample was allowed to bind overnight at 4ºC. VEGFR3 was detected using 0.4 µg/ml goat polyclonal anti-mouse VEGFR3/Flt-4 biotinylated antibody (Catalog No. BAF743, R&D Systems). For quantification, 1 µg/ml SULFO-TAG labeled streptavidin (Catalog No. R32AD-1, Mesoscale Discovery) was used at the recommended dilution of 1:500 and 2x diluted 4x Read buffer T (Catalog No. R92TC-1, Mesoscale Discovery) was added before reading on a Meso Sector plate reader. Between steps, wells were washed 5 times with 200 µl wash buffer (0.05% Tween-20 in PBS) using a AquaMax 4000 Microplate washer (Molecular Devices). During coating, blocking, and sample/antibody incubation steps plates were shaken on an orbital shaker at 600 rpm.

Immunological western blot detection
Prior immunoblot analysis, samples were depleted from immunoglobulins and enriched for glycoproteins. Per sample, 50 µl of pierce protein A magnetic beads (88846, Thermo Scientific) were mixed with pierce protein G magnetic beads (88848, Thermo Scientific) and washed twice with 1ml binding/washing buffer (2 mM $MgCl_2$, 2 mM $MnCl_2$, 2 mM $CaCl_2$, 0.5 M NaCl, 20 mM Tris HCl pH 7.5). Additionally, 50 µl of pierce protein L magnetic beads (88850, Thermo Scientific) were washed in the same manner. 20 µl of plasma was combined with 480 µl of PBS, supplemented with 0.05% Tween 20, 2 µg/ml protease inhibitor cocktail (Pi) (Sigma) cocktail and the mixture was added to the protein A/G mix. Samples were incubated at RT for 1 h on an overhead rotator and supernatants were transferred to the protein L beads. After another 1 h of incubation at RT with the overhead rotator, supernatants were collected for the glycoprotein enrichment.

The whole Ig-depleted volume was used for glycoprotein enrichment via Concanavalin A agarose conjugate (C7555, Sigma). Concanavalin A slurry was washed twice with PBS via an overhead rotator and was afterwards combined with the plasma. Binding buffer was added to a total volume of 1 ml and the mixture was incubated overnight at 4 °C on an overhead rotator. Beads were spun down for 30 s, at 2,000 g and the supernatant was discarded. Beads were washed trice for 5 min with 1 ml binding buffer and eluted twice for 30 min with 250 µl elution buffer (500 mM methyl-alpha-D-mannopyranosidase, 10 mM EDTA, 20 mM Tris HCl pH 7.5). Until further processing, the combined eluate was stored at -20 °C in lobind tubes.

Equal amounts of proteins were diluted in reducing or non-reducing sample buffer (8% SDS, 0.025% bromphenol blue, 125 mM Tris pH 6.8, +/- 10% β-mercaptoethanol), boiled for 5 min, at 95 °C and separated on 8% polyacrylamide gels. Gels were separated in an electrophoresis chamber (Biorad) filled with SDS running buffer (25 mM Tris, 240 mM Glycin, 0.1 % SDS). The separated proteins in the gels were transferred to nitrocellulose membranes using the BioRad Trans-Blot Turbo system according to the manufacturer's protocol. Membranes were blocked ovn in blocking buffer (0.1 % casein in PBS), while shacking. Afterwards, membranes were incubated with the primary antibody (BAF743) diluted in washing buffer (0.05 % Tween-20 in PBS), 0.05% BSA (v/v), 0.0005% NaAzide overnight, shaking at 4°C. Membranes were washed three times with washing buffer, while shaking for 5 min and incubated with the secondary antibody-diluted in washing buffer and 0.05% BSA (v/v) - for 45 min at RT, while shaking. After incubation, membranes were again washed three times, 5 min, while shaking and developed using ECL Western Blotting Reagents. Images were generated, using the ImageQuant LAS 4000 platform. Displayed images were cropped, using the software Photoshop 12.1.

Cleavage characterization of BACE2 for VEGFR3

HEK293 overexpression

800,000 viable HEK293 cells (ATCC) were seeded in each plate of a six well plate and grown in 1600 µl Optimem for 24 h. For transfection, 6.3 µg of lipofectamine 2000 was mixed with 196 µl serum free Optimem and a total amount of 1 µg of transfection plasmids (ratio *Bace2*: *Vegfr3* /pCDNA3.1: pFUGW = 1:3) were combined with 200 µl of serum free Optimem. After 5 min of equilibration to RT, tubes were mixed and incubated another 20 min. Afterwards the transfection mix was added to the cells. After 24 h, cells were washed once with serum free Optimem and then supplemented with 1 ml serum free Optimum. After another 24 h, lysates and media were collected for further analysis. Following plasmids were used for transfection: murine full length *Bace2* in pcDNA3.1 (kindly provided by Hyeryun Choe, Harvard Medical School, Boston (16).), murine full length 2xHa-VEGFR3-2xFlag in pFUGW (Genescript) and corresponding empty backbones (14883, Addgene).

Collection of cell supernatant media and lysates

Medium was collected, supplemented with 4 µl Pi and centrifuged at 4 °C for 10 min and 15,300 g Afterwards, medium was transferred to a fresh tube and stored at -20 °C. Cells were washed with ice cold PBS and 150 µl of STET (50 mM Tris pH 7.5, 150 mM NaCl, 2 mM EDTA, 1% TritonX-100), freshly supplemented with 2 µl/ml Pi, was used for lysis. After adding the lysis buffer, cells were incubated on ice for 10 min, scraped off and transferred into a fresh Eppendorf tube. After another 20 min incubation on ice, lysates were centrifuged at 4 °C for 10 min and 15,300 g and transferred into a fresh tube. Lysates and media were stored at -20 °C. Deglycosylated samples were generated according to the manufacturer's protocol, using PNGase F (P0704, NEB).

Immunological western blot detection

Murine VEGFR3 was detected as described. Anti HA-7 (H9658, Sigma) and Anti-FLAG M2 (F1804, Sigma) were used for detection of the utilized tags. Anti β-Actin (A5316, Sigma) and Anti-BACE2 (ab5670, Abcam) were used for the detection of Actin and BACE2.

Enzymatic and cellular BACE activity determination

BACE1 and BACE2 enzymatic activity was assessed via a FRET (Fluorescence Resonance Energy Transfer) assay (17). Therein recombinant soluble BACE1 (Amino acids/AA 1-454) or recombinant soluble BACE2 (AA 21-466) were combined with an APP derived 13 amino acids containing peptide, which contains the "Swedish" Lys-Met/Asn-Leu mutation of the APP β-secretase cleavage site. This APP peptide substrate contains two fluorophores: On the one hand (7-methodxycoumarin-4-yl) acetic acid (Mca), which is a fluorescent donor with a excitation wavelength at 320 nm and an emission at 405 nm. On the other hand, 2,4.dinitrophenyl (Dnp), a Janssen pharmaceutica proprietary quencher acceptor. Proteolysis rates are linearly related to proteolysis via BACE1/BACE2. The BACE protease/substrate mix was incubated with different amounts of inhibitor for 120 min in a 384-well plate. Fluorescence was measured via a Fluoroskan microplate fluorometer. No enzyme was present in the reaction mix for the low control. Cellular BACE1 activity was determined, using the SK-N-BE(2) neuroblastoma cell line, expressing human WT *APP*. BACE inhibitors were added to the cells in indicated concentrations and incubated for 18h and media were collected for subsequent Aβ42-MSD-assay analysis (17-19). Cellular BACE2 activity was determined using mouse insulinoma 6 (MIN6) cells, expressing murine *Vegfr3*. BACE inhibitors were added to the cells in indicated concentrations and incubated for 18h and media were collected for subsequent VEGFR3-MSD-assay analysis

VEGFR3 purification for cleavage site determination

MIN6 cells stably expressing murine *Vegfr3* were generated as follows: MIN6 cells (Addexbio) were transfected using a murine *Vegfr3* (RDC1787, R&D systems) cloned into a pcDNA 3.1 vector. Before transfection using Lipofectamine 2000, the plasmid was linearized using the restriction endonuclease ScaI. Cells were placed under selection pressure for 19 days (800 µg G418 (Gibco) per ml medium) after which 30 individual colonies were selected for expansion. Colonies were selected due to the difficulty of expanding individual MIN6 cells. Six colonies survived and could be expanded to evaluate *Vegfr3* expression via western blotting. The colony with the strongest expression was selected for downstream processing. Supernatant of 11 T75 flasks of stably *Vegfr3* transfected MIN6 cells was collected after 24-48 h of cell growth in Opti-MEM reduced serum medium (Gibco) and cell debris was removed via 5 min centrifugation at 4 °C and 1,000 rpm. Supernatants were thereafter concentrated in 30 kDa Vivaspin Turbo 15 columns via 10 min centrifugation at RT and 4,000 g. Next, VEGFR3 was immunoprecipitated using the Dynabead Protein IP kit (10007D, Thermo Scientific) and 20 µl AFL4 antibody per 50 µl beads. Non-denaturing conditions were used for the elution process and the whole eluted protein amount was deglycosylated, using the NEB deglycosylation kit (P6044). The final

protein concentration was estimated via densitometric western blot quantification against a recombinant VEGFR3 standard curve of known concentration (743-R3, R&D).

Sample preparation for cleavage site determination

Proteolytic digestion was performed using a modified protocol for single-pot solid-phase enhanced sample preparation (SP3) (20). Magnetic SeraMag A and SeraMag B beads were mixed 1:1 and a fourfold volume of H2O was added. Tubes were then placed in a magnetic rack and the supernatant discarded. Beads were rinsed two times with 500 µl H_2O and re-suspended in water to a concentration of 4 µg beads/µl. 5 µg of purified protein and 50 mM ABC were combined to a volume of 40 µl and 2.5 µl 200 mM DTT were added. Samples were mixed at 45 °C for 30 min and 1,200 rpm and subsequently 5 µl 400 mM IAA were added at 24 °C for 30 min and 1,200 rpm in the dark. Another 2.5 µl 200 mM DTT was added and 10 µl of mixed beads was combined with the protein mixture. 240 µl of 100% EtOH was mixed with the sample, followed by a 30 min incubation step at RT and 1000 rpm. For supernatant removal, beads were placed in a magnetic rack and the supernatant was discarded. Beads were then washed four times with 80% EtOH in the same manner. Samples were digested using LysN (90301, ThermoFisher). The next day, 2 µl of 8% FA were added and the bead mix supernatant was loaded on 0.2 µm filter columns, which were washed once, using 200 µl 0.1% FA and by centrifuging 2 min for 2,000 g. The flow through was dried in a SpeedVac and resuspended in 20 µl of 0.1% FA.

LC-MS/MS and data analysis for cleavage site determination

1500 fmol of the SP3 digested samples were separated on self-packed C18 columns (500 mm × 75 µm, ReproSil-Pur 120 C18-AQ, 1.9 µm; Dr. Maisch, Germany) using a binary 60 min gradient of water (A) and 100% acetonitrile (B) supplemented with 0.1% FA (0 min, 2% B; 3.5 min 5% B; 45.5 min, 35% B; 50.5 min, 60% B; 51.5 min, 95% B; 60.5 min, 95% B).

Samples were analyzed in data depended acquisition mode. Full MS spectra were acquired at a resolution of 70,000 (AGC target: 3E+6). The 10 most intense peptide ions were chosen for fragmentation by higher-energy collisional dissociation (resolution: 17,500, maximum ion trapping time: 100 ms, isolation width: 2 m/z, AGC target: 1E+5, NCE: 25%). A dynamic exclusion of 60s was applied for fragment ion spectra acquisition.

As described, Maxquant was used for raw data analysis with following differences: The digestion mode was set as unspecific and no protein LFQ was performed. The option “match between runs” was disabled. In addition to already indicated modifications, Asn -> Asp was set as a variable modification in order to consider the deglycosylation step by PNGase F. The false discovery rate for both peptides and proteins was adjusted to less than 95%. Spectra were searched only against the whole uniprot VEGFR3 sequence. Specific and semispecific peptides from all digestion mixes with a score > 40 were used as an input for the Qarip mapping tool. The C-terminus of the semi-specific peptide with closest localization to the transmembrane domain was identified via LysN digestion and chosen as the likely cleavage site.

As a quality control for the peptide’s identity, a synthetic peptide with the same sequence and sample preparation dependent modifications (cysteine carbamidomethylation, free COOH group, N → D, Peps4Life) was measured in an identical manner. The software Skyline138 (v.20.2.0.343) was subsequently used to compare the synthetic isotopic MS1 spectrum and associated fragmentation MS2 spectra with the corresponding spectra from the LysN measurement. The VEGFR3 ectodomain sequence (AA01-AA775) was used to generate the peptide and transition list.

Following Skyline settings were changed from the default: For peptide settings, LysN was set as the digestion enzyme and no missing cleavages were allowed. A 5min retention time window was used for inter sample comparison and peptides were filtered for a length between 6-25 AA. The spectral information from the LysN associated Maxquant results (see above) was used to generate a Skyline spectral library, using the default library settings. Peptide matching was picked via this library. For the transitions, y, b and precursor ions were considered. All precursor ions with charges from 1-3 and all fragment ions with charges from 1-2 were considered for analysis. First, all product ions (from ion 1 to last ion) were displayed and filtered for the 3 most intense product ions, based on the generated spectral library. The library ion matching tolerance was set to 0.055 m/z. The instrument range was defined as 140-2,000 m/z Full scan MS1 filtering was based on the number (count) of 3 isotope peaks and the mass analyzer was set to the Orbitrap system, with a resolving power of 140,000 at 200 m/z. Only scans within 5 min of the spectral library were considered. The raw file of the synthetic peptide was subsequently imported, filtered by described settings and compared to the LysN derived library. The dotp value histograms were exported to visualize precursor and fragmentation pattern similarities.

BACE2 cleavage assay of VEGFR3
The synthetic peptide Ac-AKGCVNSSASVAVEGSEDKGS with N-terminal acetylation and cysteine carbamidomethylation was order from JPT, Germany. The peptide was dissolved in 50 mM Sodium Acetate buffer (pH 4.4). Recombinant murine BACE2 (RnD Systems, US). 0.5 µg of synthetic peptide was incubated overnight at 37°C in 20 µL a 50 mM Sodium Acetate buffer (pH 4.4) with 0.5 µg recombinant BACE2, 0.5 µg BACE2 and Verubecesat (final conc. 10 µM) or without BACE2. Samples were analyzed in triplicates by mass spectrometry to identify and quantify peptide cleavage products. Peptides were purified using C18 STAGE Tips.

Peptides were separated with a binary 60 min gradient on a C18 column (180 mm × 75 µm, ReproSil-Pur 120 C18-AQ, 1.9 µm; Dr. Maisch, Germany). Samples were analyzed in data dependent acquisition mode. Full MS spectra were acquired at a resolution of 70,000 (AGC target: 3E+6). The 10 most intense peptide ions were chosen for fragmentation by higher-energy collisional dissociation (resolution: 17,500, maximum ion trapping time: 100 ms, isolation width: 2 m/z, AGC target: 1E+5, stepped NCE: 25, 27.5, 30%).

The raw data was analyzed using Maxquant version 2.5.1.0. The synthetic peptide sequence and the murine BACE2 sequence were searched with an unspecific search of peptides with at least 5 amino acids length. Extracted ion chromatograms of identified peptides were generated using the software Xcalibur Version 2.0 (Thermo Scientific). The area under the curve was calculated using Graphpad Prism 10. Sequences of cleavage products with at least 50-fold or higher between BACE2 and BACE2 plus Verubecestat were considered as BACE2 dependent cleavage products.

Endogenous VEGFR3 cleavage in LECs

Immunological detection in LECs
Human microvascular endothelial cells (HMVEC-dlyAd (CC-2543, Lonza)), referred to as lymphatic endothelial cells (LECs), were isolated from human lymphatic breast tissue and enriched for lymphatic cells. HMVEC were cultured in EBM, which unless stated otherwise, was supplemented with EBM growth kit (CC-3202, Lonza). Cells derive from a single donor and consist of > 95% LECs.

200,000 viable cells were seeded in each plate of a six well plate and grown in 1600 µl EBM for 24 h.

BACE knockdown transfections were performed as described with following differences: Instead of overexpression constructs, 10 µl of 5 µM siRNA smart pools (003747-00, 003802-00, 001810-10, Horizon) were transfected. 24h before media collection, Optimem was replaced with 800 µl of fresh EBM. For pharmacological experiments, cells were seeded as described and pre-treated with DMSO, 2 µM β-secretase inhibitor IV (565788, Merck) or 100 nM verubecestat (MBS579527, Biozol) for 2 h. Afterwards media were replaced with fresh, inhibitor supplemented EBM and cell material was collected after 24h as described. LECs were lysed, using 100 µl of STET. Immunological western blot detection was performed as described, using the anti-human VEGFR3 MAB3757 antibody (Chemicon), the same BACE2 antibody, and additionally Anti-BACE1 (5606S, Cellsignal) for detection of BACE1. Densitometric quantifications were performed on resulting images, using the software Fiji ImageJ (2.0.0-rc-67/1.52c).

Gene expression analysis in LECs
250,000 viable cells were seeded in each well of a 6 well plate and grown for 24 h in 1600 µl EBM.

For pharmacological inhibition, cells were serum starved overnight. The next morning, medium was replaced with fresh EBM, supplemented with DMSO, 100 nM verubecestat or 1.5 µg/ml VEGF-C156S (752-VC, R&D) and incubated for 100 min.

For *BACE* knockdown with subsequent inhibition, 250,000 viable cells were seeded in each well of a 6 well plate and grown for 24 h in 1600 µl EBM. Transfection was performed as indicated above and after 24 h of incubation, cells were serum starved overnight via the exchange of serum free EBM. The next morning, medium was replaced with fresh EBM, supplemented with DMSO, 100 nM verubecestat or 1.5 µg/ml VEGF-C156S and incubated for 100 min or 48 hours. Cells were collected and RNA was purified, using the Qiagen RNA preparation kit (74104), according to the manufacturer's protocol. RNA was transcribed into cDNA, using the iScript Reverse Transcription mix (1708841, Biorad), according to the manufacturer's protocol. DNA concentrations were determined via Nanodrop measurements and cDNA stocks of 25 ng/µl were generated for each sample. For primer efficiency controls via standard curves, equal amounts of cDNA were pooled and a dilution series 50, 25, 12.5, 6.25, 3.125,

1.56, 0.78 ng/µl was generated. For the qPCR reaction, a SybrGreen (1725270, Biorad) master mix (10 µl SybrGreen Buffer, 2 µl SybrGreen Primer and 8 µl cDNA) was made for each gene (qHsaCID0012647, qHsaCID0012156, qHsaCED0044238, qHsaCID0020886, qHsaCED0047198, qHsaCEP0041396, Biorad). Samples were measured in duplicates, using the default comparative Ct methods and the default 2 h ramp speed of the StepOnePlus thermocycler (Thermo Scientific). Primer efficiencies were considered for relative gene expressions and *GAPDH* was used as a homebox gene.

BACE2 dependent VEGFR3 function in zebrafish

Imaging of zebrafish facial lymphatics

Imaging of facial lymphatics followed the protocol as described in (21). In brief, embryos were subjected to fluorescence imaging at 3dpf and 5dpf following Tricaine (MS-222) treatment and embedding in 0.8% low melting agarose containing MS222. Fluorescence images were captured on a Leica SP8 inverted microscope. Images were processed and analyzed using Fiji-ImageJ. Statistical analysis was performed using Graphpad Prism 9. Brightness and contrast were adjusted for improved visualization.

Pharmacodynamic properties of VEGFR3

Proteomic analysis

Murine plasma proteomics was performed as described above for undepleted plasma.

Mesoscale-MSD-assay

Murine VEGFR3 MSD assay was performed as described above. Murine SEZ6L MSD was performed in a similar manner with following differences: Plates were coated using 3 µg/µl sheep anti-mouse SEZ6L antibody AF4804 (R&D Systems) as the capture antibody. Plasma was diluted 1:1 in blocking buffer but was not heat denatured. An 11-point, half-log SEZ6L standard curve (3000 – 0.3 pM) was generated by serial dilution of recombinant mouse SEZ6L (4804-S6, R&D Systems) in blocking buffer. The primary and secondary detection antibodies were an in-house generated rat monoclonal anti-SEZ6L antibody (clone 21A11, IgG-2a) and SULFO-TAG labeled goat anti-rat (R32AH-1, Mesoscale) used at a 1:400 and 1:500 dilution respectively. The SEZ6L monoclonal antibody was generated by immunization of Lou/c rats with murine SEZ6L ectodomain using standard hybridoma procedures as described before (17).

Human VEGFR3 Mesoscale-MSD-assays were performed like the murine MSD-assay with following differences:

40 µl plasma were diluted 1:4 in blocking buffer. A 12-point, half-log standard curve (300 – 0.001 pM) was generated by serial dilution of recombinant human VEGFR3 (349-F4-050, R&D Systems). Samples were heated for 10 min at 70 °C and 700 rpm and were cooled immediately on ice. Before loading, HBR-9 (3KC564, Scantibodies Laboratory) was added to a final concentration of 0.225 mg/ml. VEGFR3 was detected using goat polyclonal anti-human VEGFR3/Flt-4 biotinylated antibody (BAF349, R&D Systems).

NHP (*Macaca fascicularis*, female, 7-8 years of age, N = 4) sVEGFR3 was quantified using a commercial human ELISA kit (27779, Immuno-Biological Laboratories), according to the manufacturer's protocol.

Human plasma sVEGFR3 measurements.

Human plasma samples for VEGFR3 measurement were obtained from elderly Caucasian patients (aged 50-90 years) who had been diagnosed as preclinical AD, or as MCI due to AD and were enrolled in a 28-day trial designed to evaluate the safety, pharmacokinetics and pharmacodynamics of the non-selective BACE inhibitor atabecestat (Study ALZ1005; ClinicalTrials.gov, NCT01978548) (22). Samples collected at baseline and day 28 from 5 randomly selected patients that received a 50 mg daily oral dose of atabecestat in study ALZ1005 were analyzed for sVEGFR3 levels as described above.

NHP CSF Aβ measurement.

The CSF samples for Aβ measurement were obtained 8 hours after last administration from four NHPs treated for seven days with 10 mg/kg verubecestat. Aβ40 was quantified using a Human β Amyloid (1-40) ELISA Kit (Wako) and Aβ42 was quantified using a Human β Amyloid (1-42) ELISA Kit High Sensitive (WAKO), according to the manufacturer's protocols.

Statistics

Statistical analysis was done using GraphPad Prism, Microsoft Excel and Perseus software. Details are indicated in the figure legends. Analyses were performed using the unpaired t-test and by one-way or two-way ANOVA with Bonferroni's multiple comparison test. When multiple t-tests were applied, p-values were either corrected by Bonferroni's multiple comparison test, or t-tests were combined with permutation-based FDR correction.

Study approval

All murine animal procedures were carried out in accordance with the European Communities Council Directive (86/609/EEC) and onsite procedures were approved by the committee responsible for animal ethics of the government of Upper Bavaria (02-19-067) and Belgium (LA1100119). Zebrafish experiments were performed in accordance with animal protection standards of the Ludwig-Maximilians University Munich and were approved by the government of Upper Bavaria (Regierung von Oberbayern, Munich, Germany) or the animal ethics committee at the University of Münster. Fish maintenance was in accordance with FELASA guidelines (5). All NHP experiments and sampling were conducted by Shionogi & Co., Ltd and performed according to AAALAC and Shionogi ethics committee.

Data Availability

The mass spectrometry proteomics data have been deposited to the ProteomeXchange Consortium via the PRIDE partner repository (23) with the dataset identifiers PXD041577, PXD041579, and PXD042669. Supporting data values are provided as supplementary excel file.

References

1. Dominguez D, Tournoy J, Hartmann D, Huth T, Cryns K, Deforce S, et al. Phenotypic and biochemical analyses of BACE1- and BACE2-deficient mice. *The Journal of biological chemistry.* 2005;280(35):30797-806.
2. Voytyuk I, Mueller SA, Herber J, Snellinx A, Moechars D, van Loo G, et al. BACE2 distribution in major brain cell types and identification of novel substrates. *Life science alliance.* 2018;1(1):e201800026.
3. Miyazaki T, Hashimoto K, Uda A, Sakagami H, Nakamura Y, Saito SY, et al. Disturbance of cerebellar synaptic maturation in mutant mice lacking BSRPs, a novel brain-specific receptor-like protein family. *FEBS letters.* 2006;580(17):4057-64.
4. Ong-Pålsson E, Njavro JR, Wilson Y, Pigoni M, Schmidt A, Müller SA, et al. The β-Secretase Substrate Seizure 6-Like Protein (SEZ6L) Controls Motor Functions in Mice. *Molecular neurobiology.* 2022;59(2):1183-98.
5. Aleström P, D'Angelo L, Midtlyng PJ, Schorderet DF, Schulte-Merker S, Sohm F, et al. Zebrafish: Housing and husbandry recommendations. *Laboratory animals.* 2020;54(3):213-24.
6. Kimmel CB, Ballard WW, Kimmel SR, Ullmann B, and Schilling TF. Stages of embryonic development of the zebrafish. *Developmental dynamics : an official publication of the American Association of Anatomists.* 1995;203(3):253-310.
7. Gordon K, Schulte D, Brice G, Simpson MA, Roukens MG, van Impel A, et al. Mutation in vascular endothelial growth factor-C, a ligand for vascular endothelial growth factor receptor-3, is associated with autosomal dominant milroy-like primary lymphedema. *Circulation research.* 2013;112(6):956-60.
8. Hogan BM, Herpers R, Witte M, Heloterä H, Alitalo K, Duckers HJ, et al. Vegfc/Flt4 signalling is suppressed by Dll4 in developing zebrafish intersegmental arteries. *Development.* 2009;136(23):4001-9.
9. Okuda KS, Astin JW, Misa JP, Flores MV, Crosier KE, and Crosier PS. lyve1 expression reveals novel lymphatic vessels and new mechanisms for lymphatic vessel development in zebrafish. *Development.* 2012;139(13):2381-91.
10. Roman BL, Pham VN, Lawson ND, Kulik M, Childs S, Lekven AC, et al. Disruption of acvrl1 increases endothelial cell number in zebrafish cranial vessels. *Development.* 2002;129(12):3009-19.
11. Münzel EJ, Schaefer K, Obirei B, Kremmer E, Burton EA, Kuscha V, et al. Claudin k is specifically expressed in cells that form myelin during development of the nervous system and regeneration of the optic nerve in adult zebrafish. *Glia.* 2012;60(2):253-70.
12. Rappsilber J, Mann M, and Ishihama Y. Protocol for micro-purification, enrichment, pre-fractionation and storage of peptides for proteomics using StageTips. *Nature protocols.* 2007;2(8):1896-906.
13. Tyanova S, Temu T, and Cox J. The MaxQuant computational platform for mass spectrometry-based shotgun proteomics. *Nature protocols.* 2016;11(12):2301-19.
14. Bruderer R, Bernhardt OM, Gandhi T, Miladinović SM, Cheng LY, Messner S, et al. Extending the limits of quantitative proteome profiling with data-independent acquisition and application to acetaminophen-treated three-dimensional liver microtissues. *Molecular & cellular proteomics : MCP.* 2015;14(5):1400-10.
15. Tyanova S, Temu T, Sinitcyn P, Carlson A, Hein MY, Geiger T, et al. The Perseus computational platform for comprehensive analysis of (prote)omics data. *Nature methods.* 2016;13(9):731-40.
16. Farzan M, Schnitzler CE, Vasilieva N, Leung D, and Choe H. BACE2, a beta -secretase homolog, cleaves at the beta site and within the amyloid-beta region of the amyloid-beta precursor protein. *Proceedings of the National Academy of Sciences of the United States of America.* 2000;97(17):9712-7.

17. Pigoni M, Wanngren J, Kuhn PH, Munro KM, Gunnersen JM, Takeshima H, et al. Seizure protein 6 and its homolog seizure 6-like protein are physiological substrates of BACE1 in neurons. *Molecular neurodegeneration.* 2016;11(1):67.
18. Struyfs H, Van Broeck B, Timmers M, Fransen E, Sleegers K, Van Broeckhoven C, et al. Diagnostic Accuracy of Cerebrospinal Fluid Amyloid-β Isoforms for Early and Differential Dementia Diagnosis. *Journal of Alzheimer's disease : JAD.* 2015;45(3):813-22.
19. Van Broeck B, Timmers M, Ramael S, Bogert J, Shaw LM, Mercken M, et al. Impact of frequent cerebrospinal fluid sampling on Aβ levels: systematic approach to elucidate influencing factors. *Alzheimer's research & therapy.* 2016;8(1):21.
20. Hughes CS, Moggridge S, Müller T, Sorensen PH, Morin GB, and Krijgsveld J. Single-pot, solid-phase-enhanced sample preparation for proteomics experiments. *Nature protocols.* 2019;14(1):68-85.
21. Hußmann M, Schulte D, Weischer S, Carlantoni C, Nakajima H, Mochizuki N, et al. Svep1 is a binding ligand of Tie1 and affects specific aspects of facial lymphatic development in a Vegfc-independent manner. *eLife.* 2023;12.
22. Timmers M, Streffer JR, Russu A, Tominaga Y, Shimizu H, Shiraishi A, et al. Pharmacodynamics of atabecestat (JNJ-54861911), an oral BACE1 inhibitor in patients with early Alzheimer's disease: randomized, double-blind, placebo-controlled study. *Alzheimer's research & therapy.* 2018;10(1):85.
23. Perez-Riverol Y, Bai J, Bandla C, García-Seisdedos D, Hewapathirana S, Kamatchinathan S, et al. The PRIDE database resources in 2022: a hub for mass spectrometry-based proteomics evidences. *Nucleic acids research.* 2022;50(D1):D543-d52.

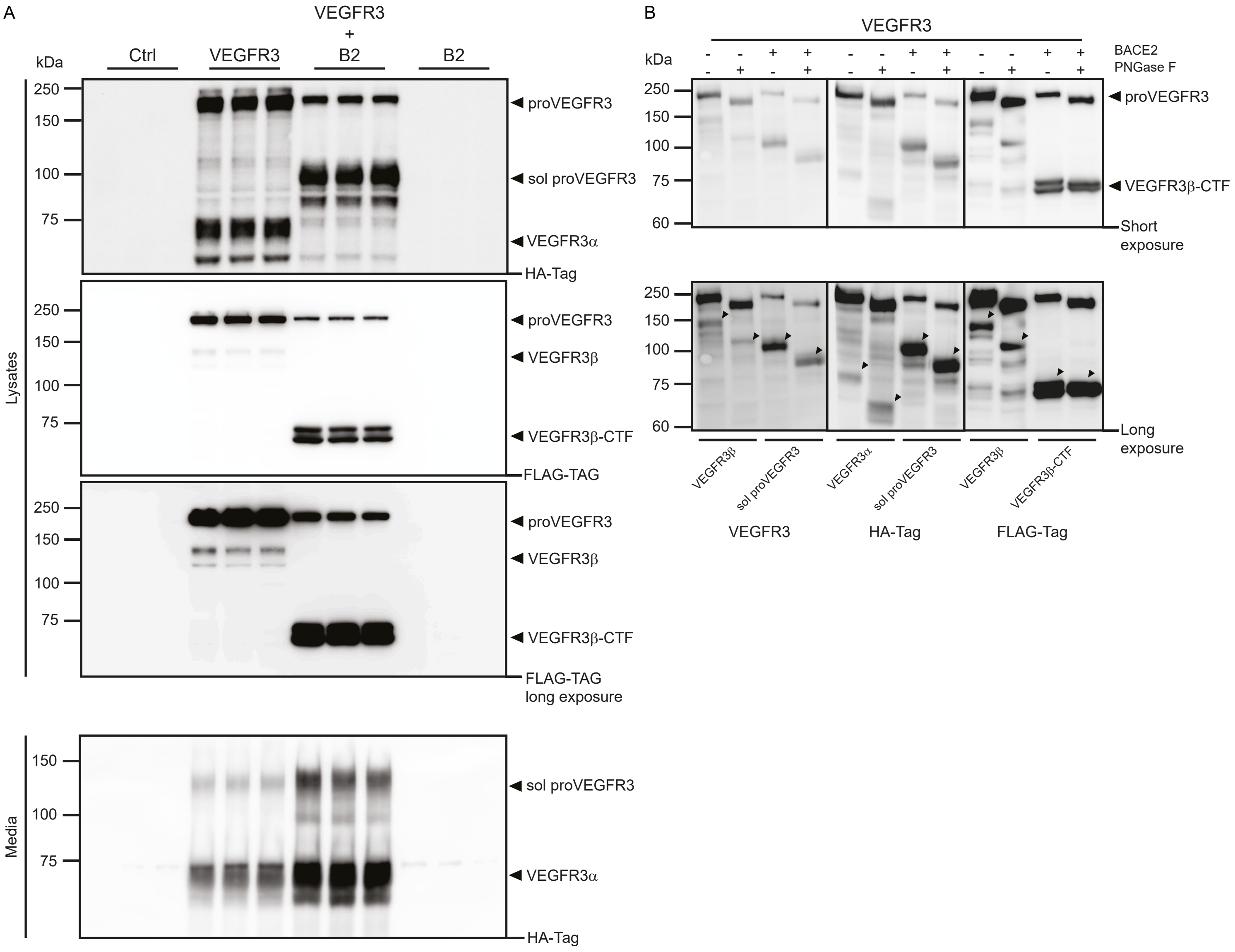

Supplementary Figure 1

Characterization of VEGFR3 fragments upon VEGFR3 expression in HEK cells. (A) Immunoblot detection of VEGFR3 in transfected HEK293 cell lysates and conditioned media with empty control plasmids (Ctrl), *Bace2* (B2), *Vegfr3* and *Vegfr3* + *Bace2* co-expression (VEGFR3 + B2). Lysates were blotted for the HA and FLAG tag at the N- and C-terminus of VEGFR3, for BACE2 and actin. Media were blotted for the N-terminal HA tag on VEGFR3. For better visualization of VEGFRβ, an enhanced exposure of the lysate is shown in the lower panel. **(B)** Immunological detection of VEGFR3, HA and FLAG fragments, after PNGase F deglycosylation. The lower panels show a longer exposure time of the blots. Arrowheads indicate bands of VEGFR3 fragments (as indicated at the bottom) before and after size shift. For fragment nomenclature, see Figure 2A. sVEGFR3 is not detectable under reducing conditions. The additional BACE2 cleavage product sol proVEGFR3 in the lysates appears at around 100 kDa, which derives from cleavage of the not yet maturely glycosylated proVEGFR3 early in the secretory pathway. Representative data from three independent experiments.

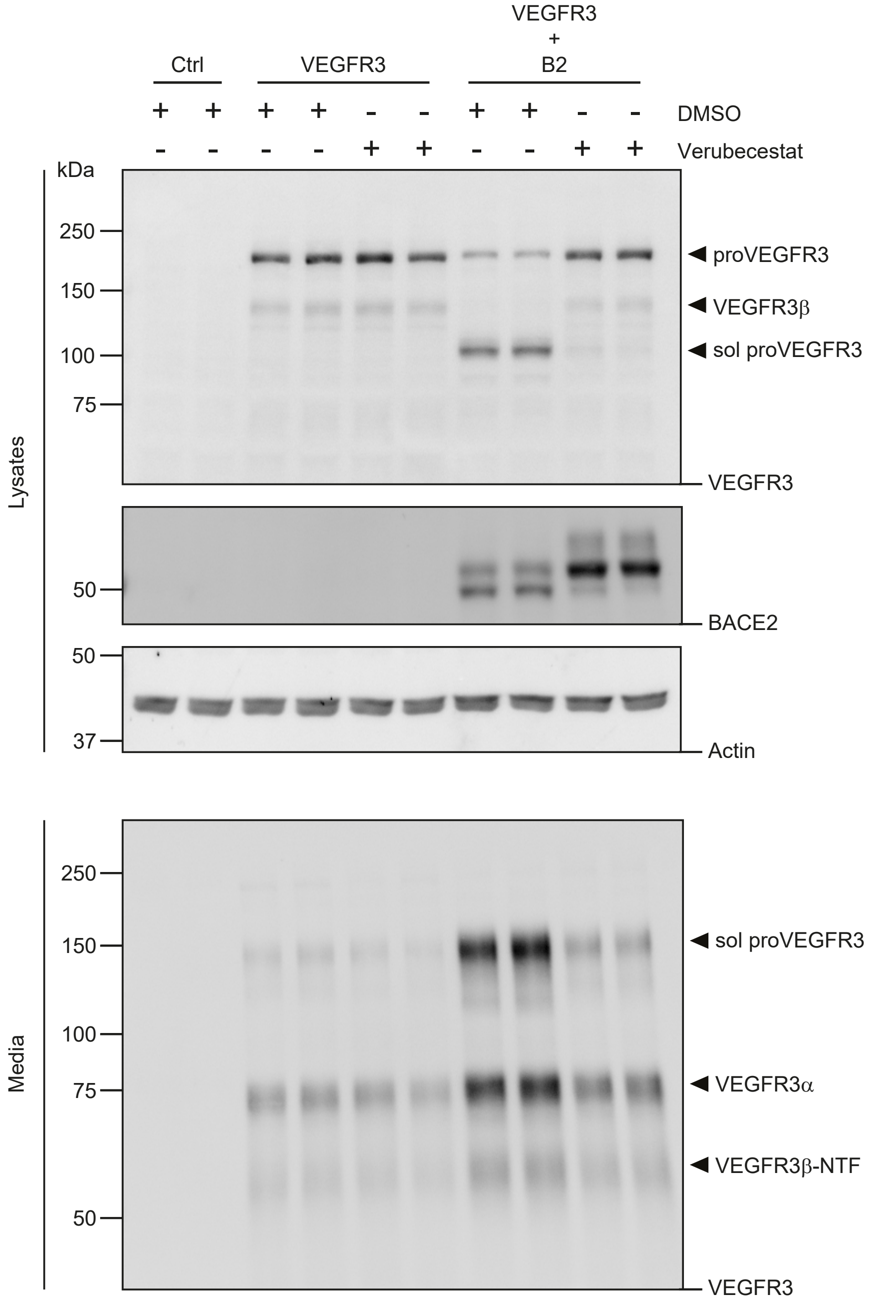

Supplementary Figure 2

Verubecestat blocks VEGFR3 cleavage in *Vegfr3*- and *Bace2*-overexpressing HEK293 cells. Immunoblot detection of VEGFR3 in lysates and media of HEK293 cells transfected with empty control plasmids (Ctrl), *Vegfr3* and *Vegfr3* + *Bace2* (VEGFR3 + B2). Cells were either treated with verubecestat (+) or with DMSO (-) as control. Lysates and media were probed with an anti-VEGFR3 antibody which targets the ectodomain (see also Figure 2A). Lysates were further blotted for BACE2 and actin. sVEGFR3 is not detectable under reducing conditions. The additional BACE2 cleavage product sol proVEGFR3 in the lysates appears at around 100 kDa, which derives from cleavage of the not yet maturely glycosylated proVEGFR3 early in the secretory pathway. Representative data from two independent experiments are shown.

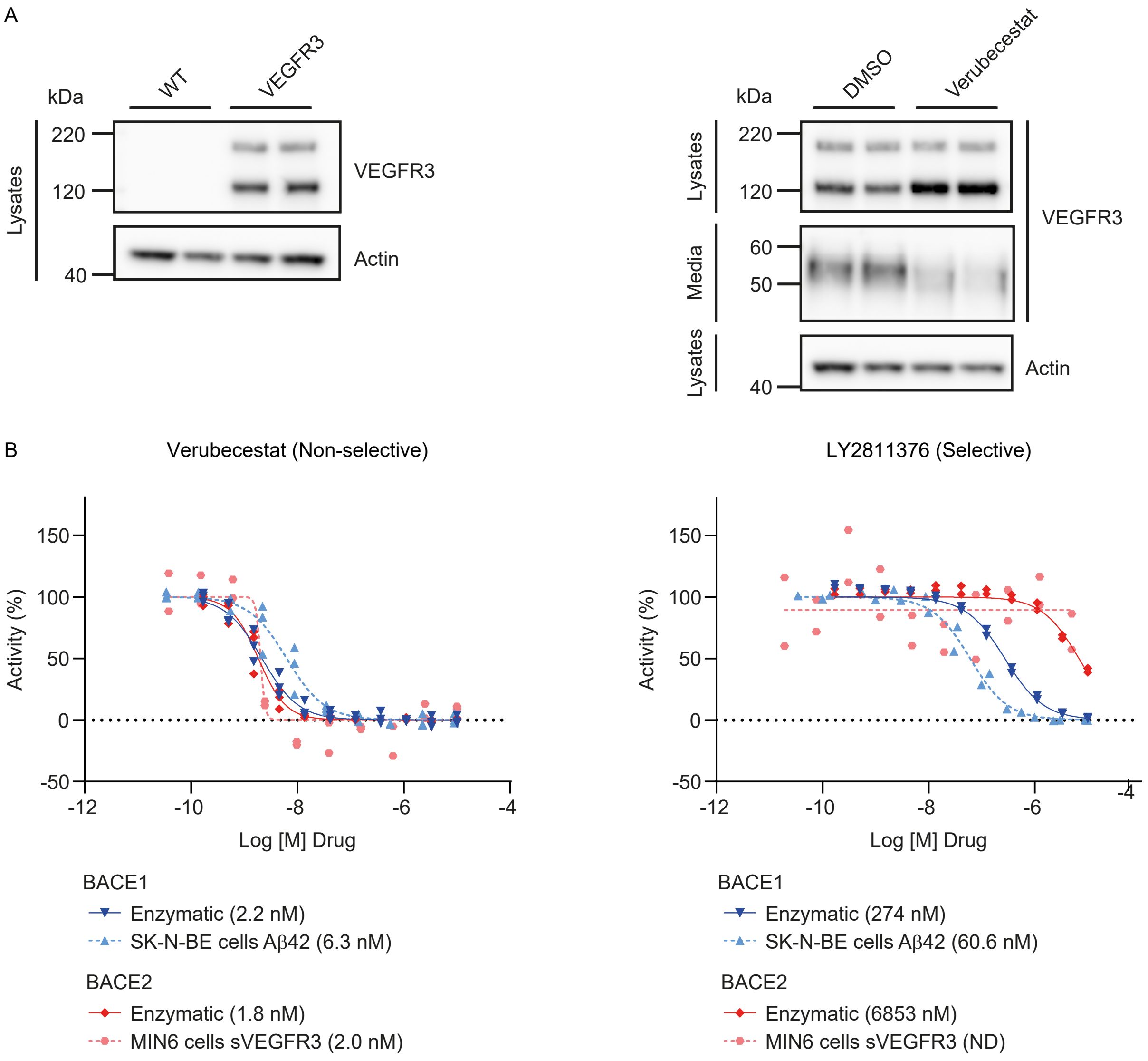

Supplementary Figure 3

BACE2-dependent VEGFR3 cleavage in stably *Vegfr3*-transfected MIN6 cells and enzymatic cleavage assays. (A) Immunoblot detection of VEGFR3 in *Vegfr3*-overexpressing MIN6 cell lysates (VEGFR3) compared to the non-transfected wildtype (WT) MIN6 cells (left panel) and in lysates as well as conditioned media from VEGFR3-overexpressing MIN6 cells treated with 100 nM verubecestat or DMSO as control (right panel). **(B)** The plots depict inhibition curves of BACE1 and BACE2 upon application of the unselective BACE inhibitor verubecestat, which blocks both BACE1 and BACE2, and the BACE1-selective inhibitor LY2811376. Blue and red lines refer to BACE1- and BACE2-specific assays, respectively. Dark continuous lines represent enzymatic, fluorescent peptide cleavage assays, where recombinant BACE1 or BACE2 were incubated with an APP-derived fluorophore-coupled quenched peptide. Upon cleavage by BACE1 or BACE2, the quenching is terminated and fluorescence is observed. When BACE1 and BACE2 are inhibited, less peptide is cleaved and the fluorescence decreases (for details see methods section). Bright dotted lines represent results from cell culture-based MSD-immunoassays, with generation of the Aβ42 peptide from neuroblastoma SK-N-BE cells (which express endogenous *BACE1*) as a readout specifically for BACE1 activity and with generation of sVEGFR3 from VEGFR3-transfected MIN6 cells (which express endogenous BACE2) as a readout specifically for BACE2 activity.
Data Information: Individual data points (N = 1-4) are shown. Brackets indicate the derived IC50 values for the given inhibitors in these assays.

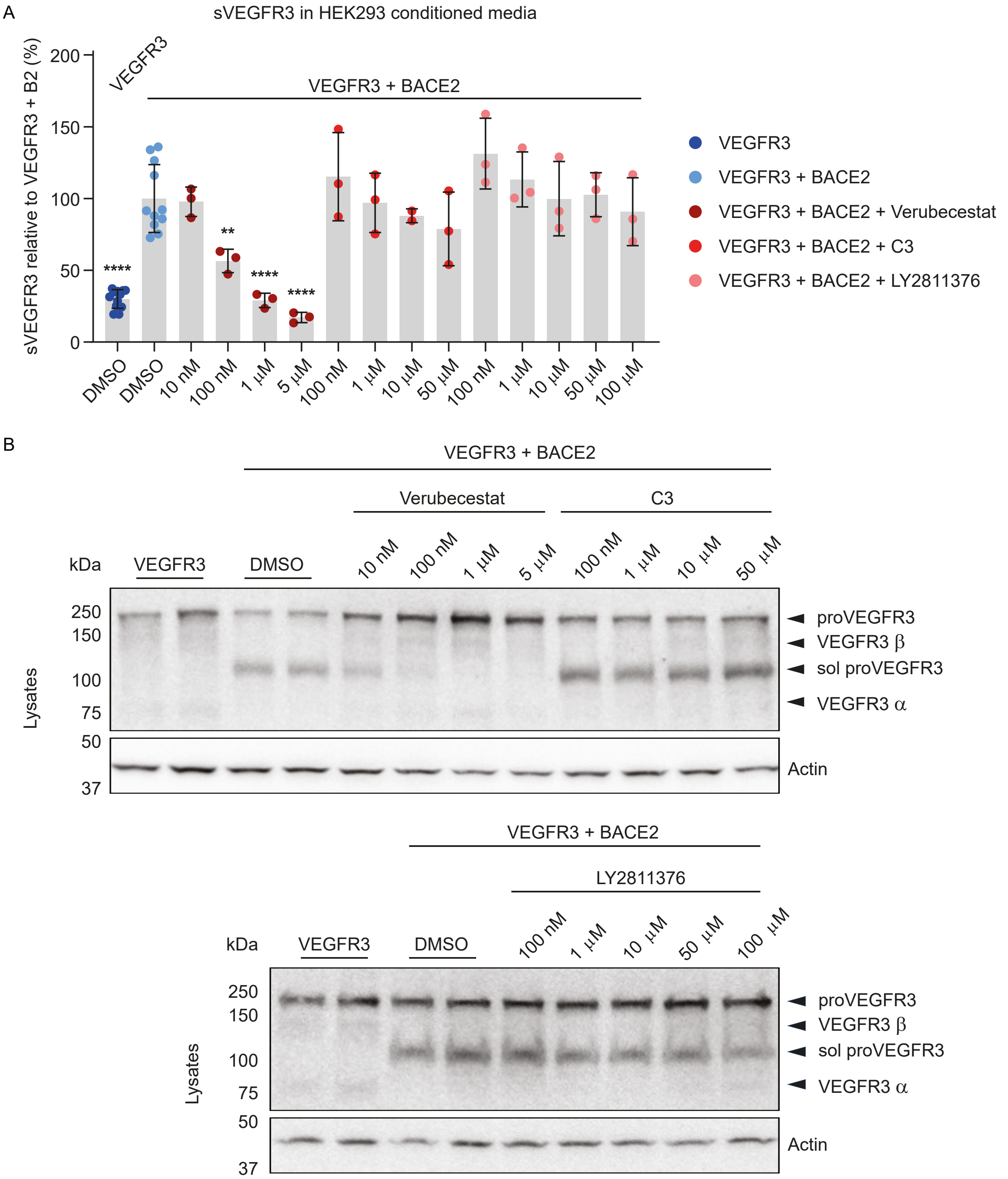

Supplementary Figure 4

Verubecestat inhibits BACE2 cleavage of VEGFR3 in *Vegfr3*- and *Bace2*-overexpressing HEK293 cells.
HEK293 cells were either transfected with *Vegfr3* + *Bace2* or *Vegfr3* and a control plasmid. Cells transfected with *Vegfr3* + *Bace2* were treated with verubecestat (inhibiting BACE1/2), C3 (BACE1-preferring), LY2811376 (BACE1-preferring) or DMSO as control for 24 h. sVEGFR3 in the conditioned media was then quantified by **(A)** ELISA, and VEGFR3 in the lysate was detected and showed by (**B**) immunoblotting.

Data information: Data in A are shown with mean and SD. N = 12 (VEGFR3), N = 11 (VEGFR3 + BACE2), N = 2-3 for all other groups. Allconditions were compared against the VEGFR3 + BACE2 DMSO control by one-way ANOVA with Dunnett's multiple comparison.

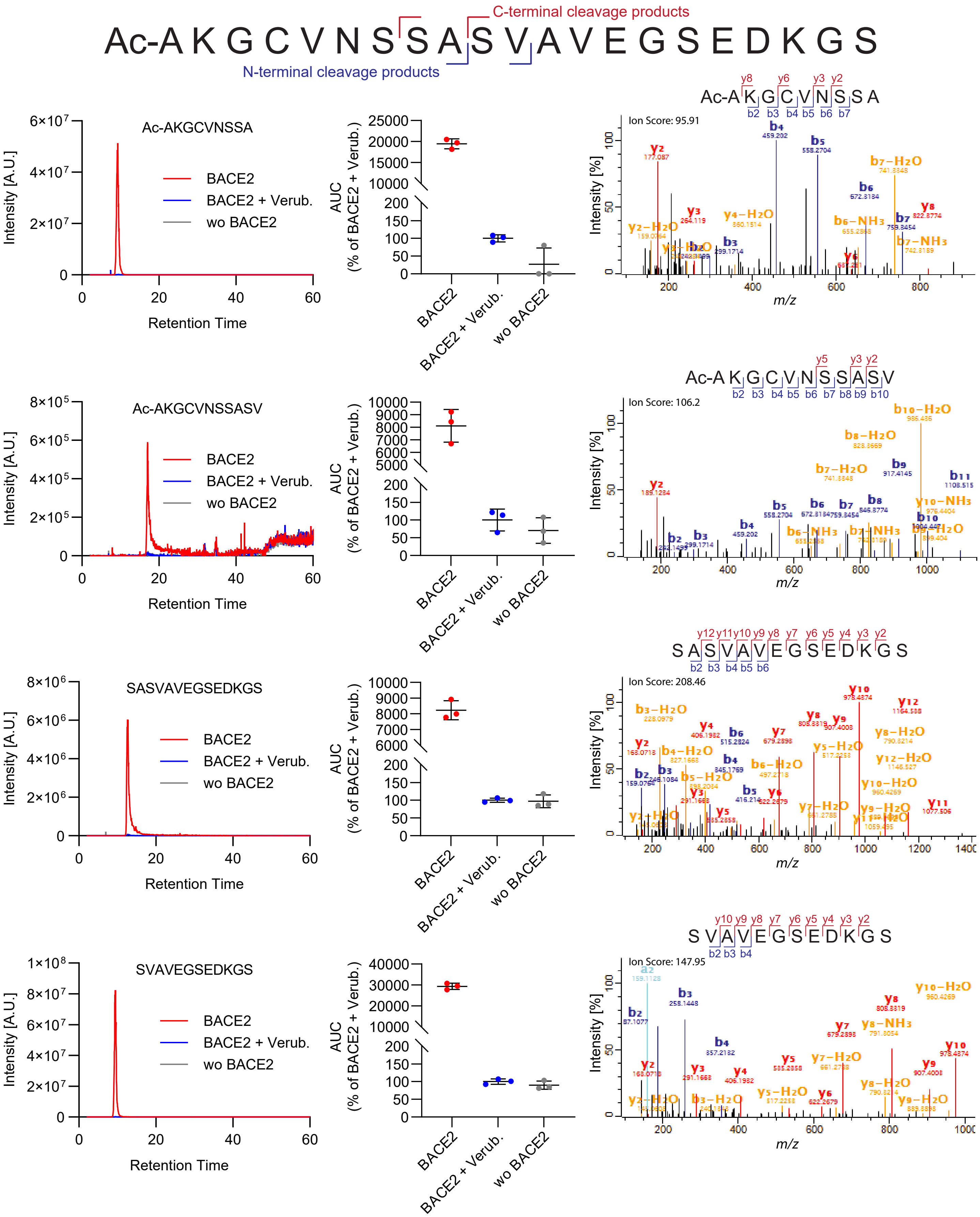

Supplementary Figure 5

BACE2 cleavage assay of VEGFR3. The synthetic peptide Ac-AKGCVNSSASVAVEGSEDKGS with N-terminal acetylation and cysteine carbamidomethylation was incubated overnight at 37°C in a 50 mM Sodium Acetate buffer (pH 4.4) with recombinant BACE2, BACE2 and Verubecesat or without BACE2. Samples were analyzed in triplicates by mass spectrometry to identify and quantify peptide cleavage products. The sequence of the peptide with indicated identified cleavage products are shown on top. Four peptides showed at least a 50-fold higher abundance between incubation with BACE2 and BACE2 + Verubecestat. Representative extracted ion chromatograms of those 4 peptides are shown below. The area under the curve (AUC) was used for relative quantification. The dot plots show the relative abundance of the peptides compared to the control BACE2 + Verubecestat. Fragment ion spectra for the identification of the peptides are shown on the right with indication of the fragmention products within their sequences.

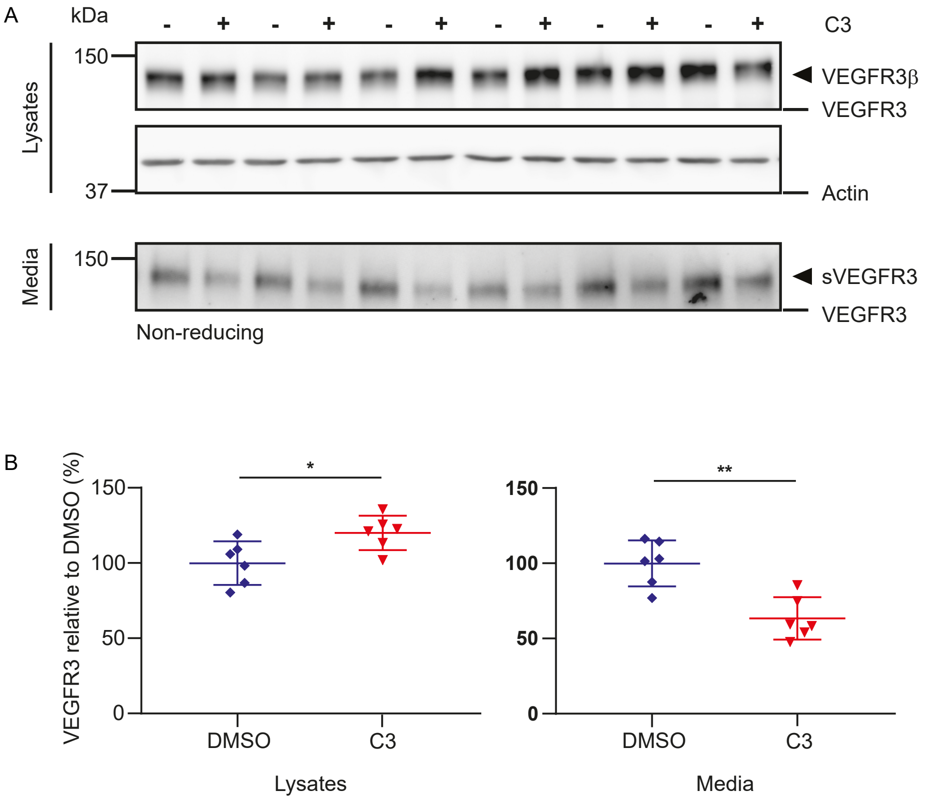

Supplementary Figure 6

Validation of the endogenous cleavage of VEGFR3 in LECs. (A) Immunoblot analysis of cells treated with DMSO (-) or 500 nM of the BACE inhibitor C3 (+). Lysates were blotted for VEGFR3 and actin. Media were blotted for sVEGFR3. **(B)** Corresponding densitometric quantifications, derived from VEGFR3β-CTF (lysate) and sVEGFR3 (medium).

Data Information: All dot plots were normalized on the vehicle control mean and depict mean and SD, with p-values (* $p < 0.05$, ** $p < 0.01$), calculated by unpaired t-test. Data derives from N = 6 biological replicates.

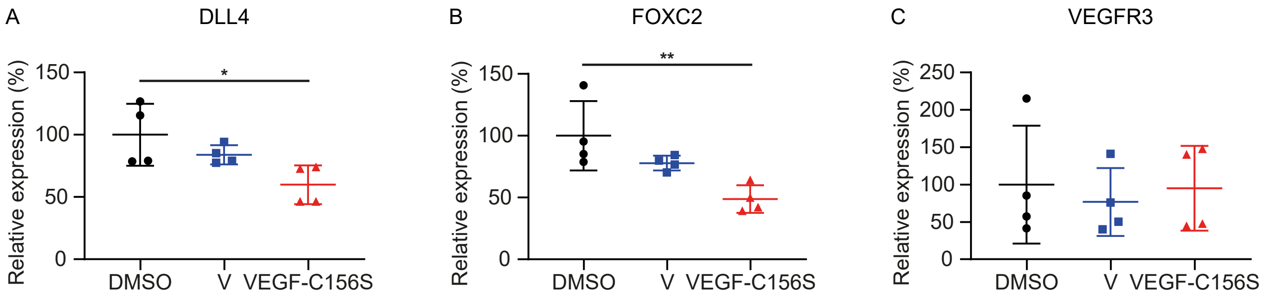

Supplementary Figure 7

VEGFR3 signaling in LECs after prolonged treatment with verubecestat or VEGF-C. Gene expression levels of **(A)** DLL4, **(B)** FOXC2 and **(C)** VEGFR3 after 48 h of treatment with 100 nM verubecestat (V) or 1.5 µg/mL VEGF-C156S. DMSO was used as control. Data were normalized on the control mean and are depicted with mean and SD. Data derive from N = 4 biological replicates in each group. The treatment groups were compared to the DMSO control by one-way ANOVA combined with Dunnett's multiple comparison test (* $p < 0.05$, ** $p < 0.01$).

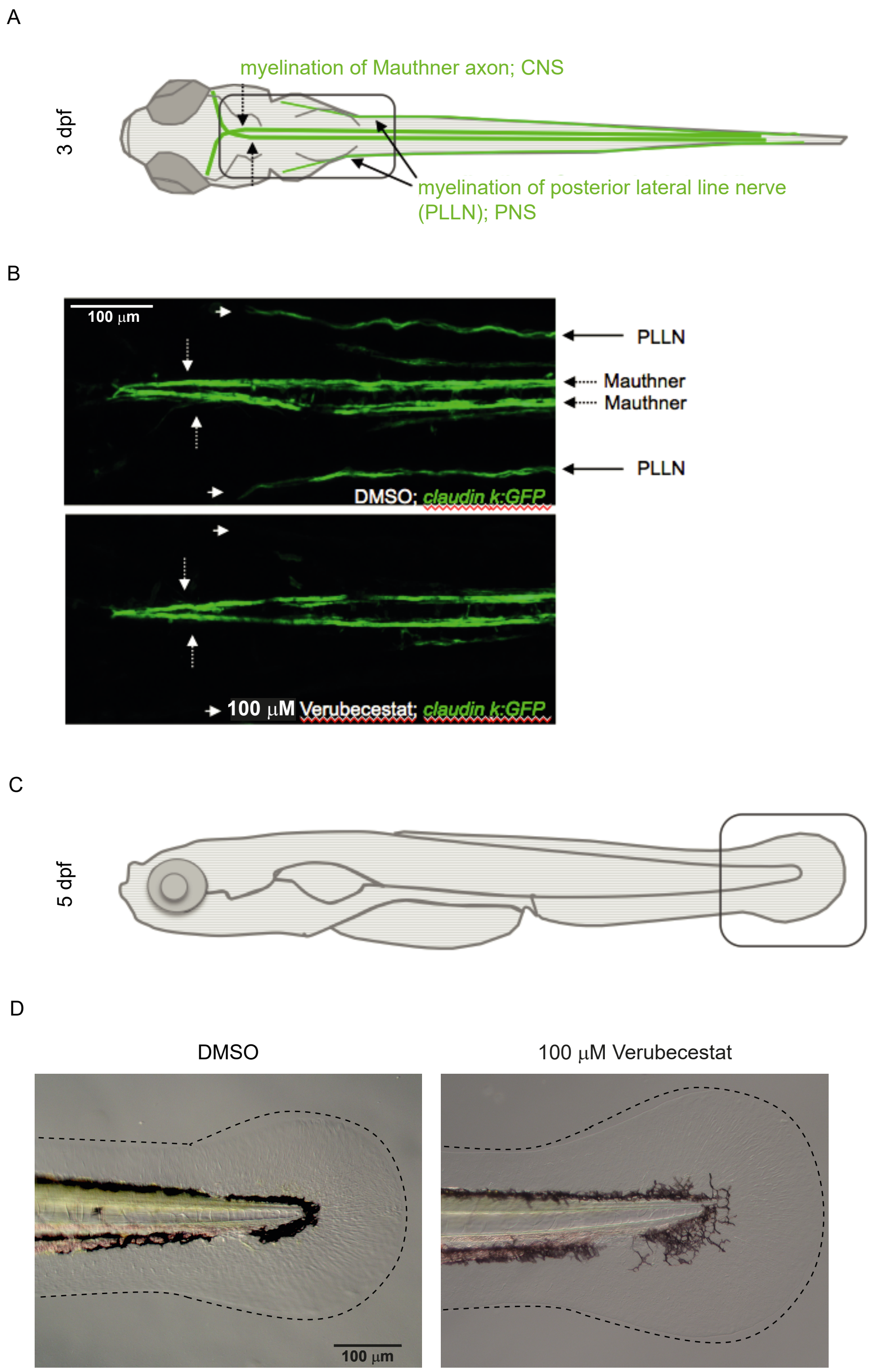

Supplementary Figure 8

Influence of BACE inhibitors on zebrafish PNS myelination and fin melanophore migration. (A) Myelination of Mauthner axons (CNS, dotted arrows) and lateral line nerves (PNS, arrows) are visualized by claudin k:GFP in zebrafish larvae; schematic view from dorsal. **(B)** Dorsal views of larvae at 3 dpf, expressing the transgene claudin K:GFP and visualizing myelinating cells in the CNS (dotted arrows), treated with DMSO or 100 µM verubecestat. At 3 dpf, myelination of the posterior lateral line nerves (PLLN) is absent after BACE inhibitor treatment while the myelinated Mauthner axons of the CNS are unaffected (N ≥ 22). Scale bar in B represents 100 µm. **(C)** Schematic lateral view of a 3 dpf old larvae. Boxed tail tip corresponds to imaged area in D. **(D)** Lateral views of the tail tip of zebrafish larvae at 3 dpf treated with 1% DMSO or 100 µM verubecestat. In Bace inhibitor-treated larvae the melanophores are more dispersed and migrate into the fin fold compared to DMSO treated control larvae (N ≥ 20). Scale bar in D represents 100 µm, dashed line shows fin outline.

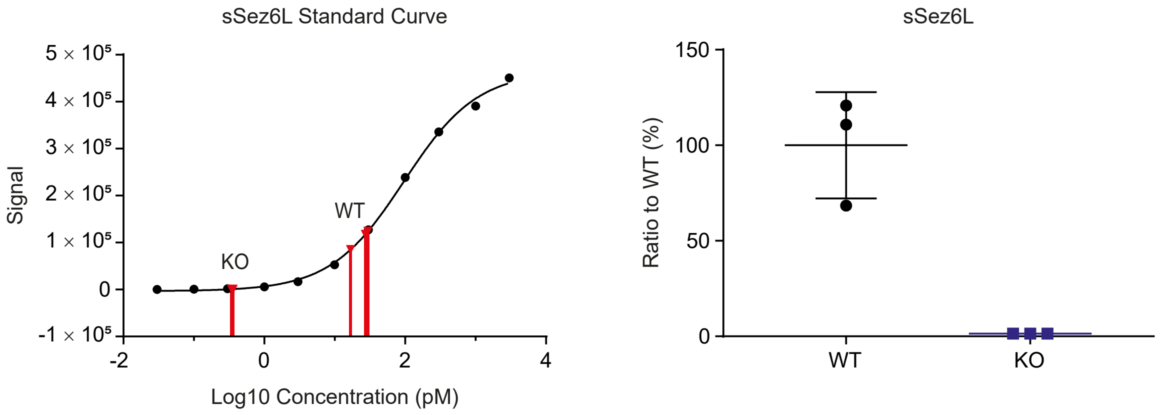

Supplementary Figure 9

Set-up of sSEZ6L MSD-immunoassay. Left: Standard curve of sSEZ6L, where the y-axis depicts signal intensities resulting from the corresponding standard concentrations on the x-axis. Measured plasma sSEZ6L concentrations from WT and SEZ6L KO (KO) mice are indicated with red lines. Right: Dot plot representation of the measured plasma sSEZ6L, as indicated in red in the left panel. Data are normalized to the mean of the WT samples to graphically present the degree of sSEZ6L reduction upon KO of SEZ6L.
Data Information: All standard curve data points depict the mean of 2 technical replicates. The dot plot was normalized on the WT control mean and depicts mean and SD. Data derive from N = 3 biological replicates.

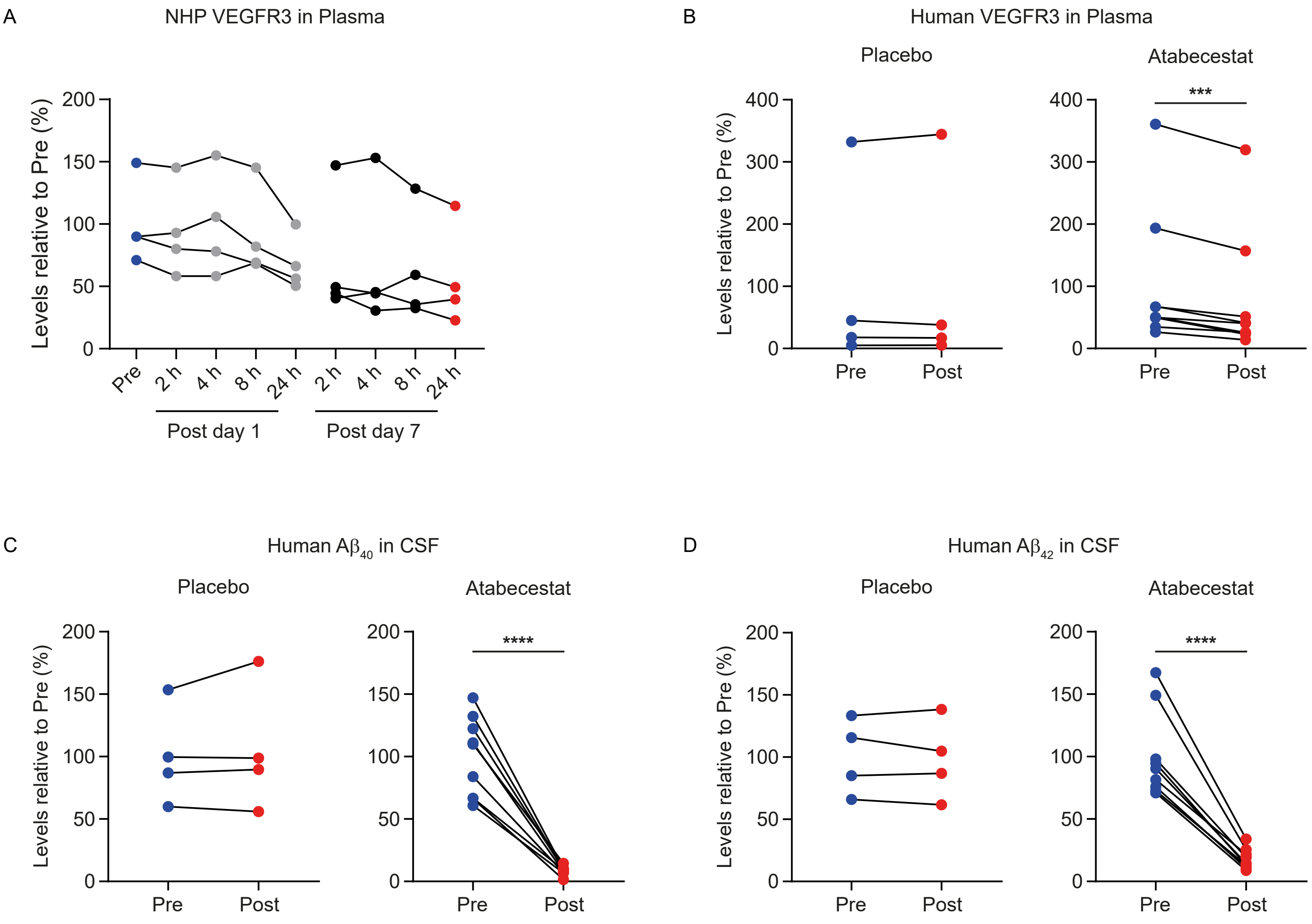

Supplementary Figure 10

NHP and human sVEGFR3 plasma levels and human CSF levels of Aβ40 and Aβ42. (A) Relative ELISA quantifications of sVEGFR3 from the plasma of verubecestat-treated NHPs (N = 4) before (Pre) and at different time points after treatment. Data were normalized on the pre-dose mean. Pre-dose (blue dots) as well as post-dose values (red dots) from day 7, 24 h are the same as shown in Figure 7D. sVEGFR3 levels decreased within the first day of treatment, followed by a plateau phase with almost constant concentrations until post day 7 for three of the animals. In the fourth animal (top line) sVEGFR3 also decreased after dosing, but returns to baseline before the next dose, potentially due to a faster drug metabolism in that NHP.
Relative ELISA quantification of **(B)** plasma sVEGFR3 as well as of **(C)** Aβ40 and **(D)** Aβ42 in CSF of clinical trial participants treated with atabecestat (N = 9) or a placebo (N = 4). Data were normalized on the pre-dose mean. Individual post-dose/pre-dose ratios of the data are shown in Figure 7E. For statistical analysis, the paired t-test was applied (*** $p < 0.001$, **** $p < 0.0001$).

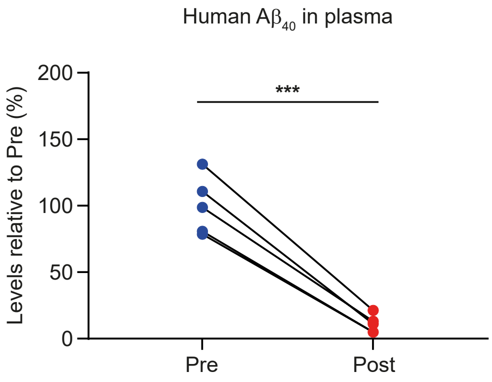

Supplementary Figure 11

Effect of BACE inhibition on plasma Aβ40 levels. Aβ1-40 (Aβ40) concentrations were determined by immunoassay in plasma from atabecestat-treated clinical trial participants (N = 5) before (pre) and after (post) treatment. Plasma Aβ40 data for the selected individuals were extracted from a previous publication (1) and were normalized on the pre-dose mean value. For statistical analysis, the paired t-test was applied (*** $p < 0.001$).

Suppl. Table 2: IC_{50} values of reference BACE inhibitors on BACE1 and BACE2 proteolytic activtiy.

	Enzymatic IC_{50} (nM)			Binding Ki (nM)		
	BACE1	BACE2	B1/B2 Ratio	BACE1	BACE2	B1/B2 Ratio
Cpd 89[a]	7.8	33.1	4.3	1.0	10.9	10.7
LY2811376	274	6853	25	92.4	2741	30
Verubecestat	2.2	1.8	0.8	0.4	0.4	1
Atabecestat	9.3	42.7	4.6	0.9	10.3	11.5

IC50 values were determined using BACE1 and BACE2 enzymatic assays.
[a]Compound 89 (2, 3) is a potent low-nanomolar BACE1 inhibitor with an approximate 4-fold selectivity over BACE2.

Suppl. Table 3: Used DIA windows for murine plasma. Optimized m/z window distribution for Sequential Window Acquisition of All Theoretical Mass Spectra (Swath-MS)-based data independent acquisition.

Window	m/z start	m/z end	Center	Isolation width [m/z]
1	300	385	342.5	85
2	384	424	404	40
3	423	453	438	30
4	452	480	466	28
5	479	505	492	26
6	504	529	516.5	25
7	528	552	540	24
8	551	575	563	24
9	574	598	586	24
10	597	621	609	24
11	620	645	632.5	25
12	644	671	657.5	27
13	670	698	684	28
14	697	726	711.5	29
15	725	759	742	34
16	258	796	777	38
17	795	841	818	46
18	840	901	870.5	61
19	900	1051	975.5	151
20	150	1402	1226	352

References

1. Timmers M, Streffer JR, Russu A, Tominaga Y, Shimizu H, Shiraishi A, et al. Pharmacodynamics of atabecestat (JNJ-54861911), an oral BACE1 inhibitor in patients with early Alzheimer's disease: randomized, double-blind, placebo-controlled study. *Alzheimer's research & therapy.* 2018;10(1):85.
2. Hilpert H, Guba W, Woltering TJ, Wostl W, Pinard E, Mauser H, et al. β-Secretase (BACE1) inhibitors with high in vivo efficacy suitable for clinical evaluation in Alzheimer's disease. *Journal of medicinal chemistry.* 2013;56(10):3980-95.
3. Nash A, Gijsen HJM, Hrupka BJ, Teng KS, Lichtenthaler SF, Takeshima H, et al. BACE inhibitor treatment of mice induces hyperactivity in a Seizure-related gene 6 family dependent manner without altering learning and memory. *Scientific reports.* 2021;11(1):15084.
