# Supplementary material for: The Alzheimer’s disease–linked protease BACE2 cleaves VEGFR3 and modulates its signaling
Source: J Clin Invest. 2024 Jun 18;134(16):e170550. doi: 10.1172/JCI170550 (PMC11324312; doi:10.1172/JCI170550)
Supplement: Unedited blot and gel images [file jci-134-170550-s093.pdf]

Full unedited blots for Figure 1D

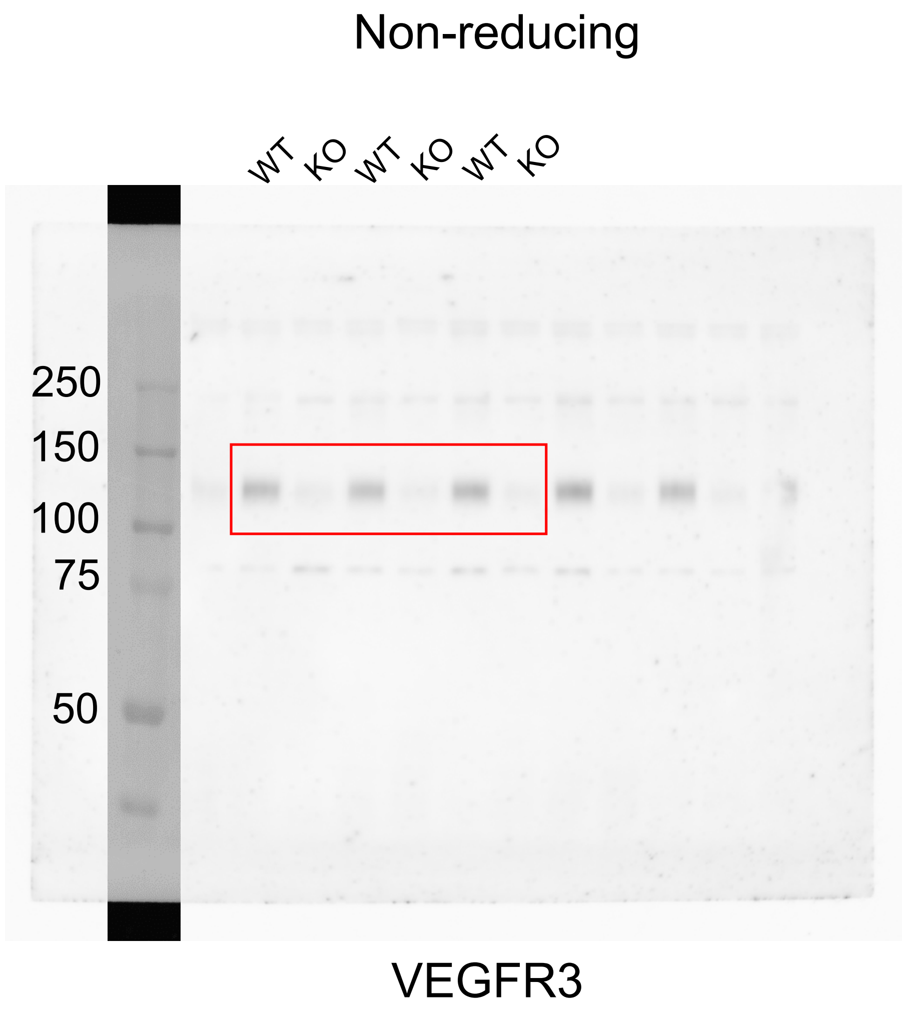

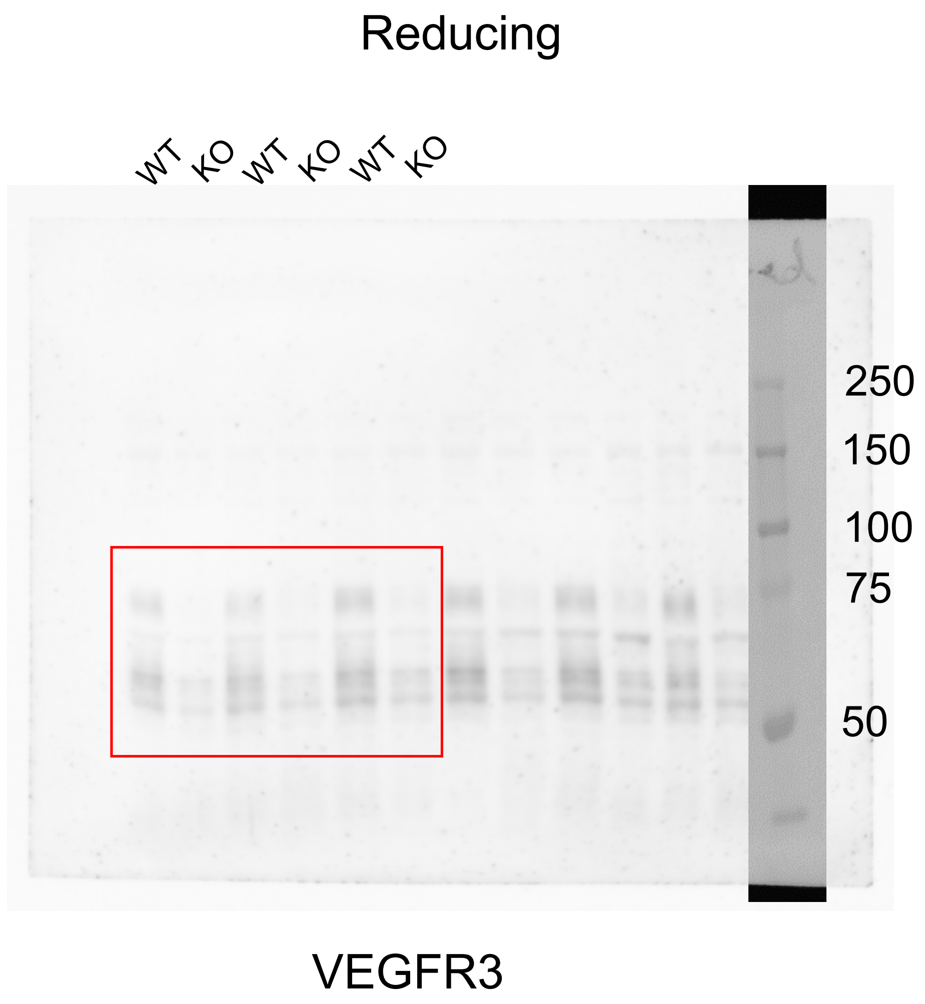

Full unedited blots for Figure 2B

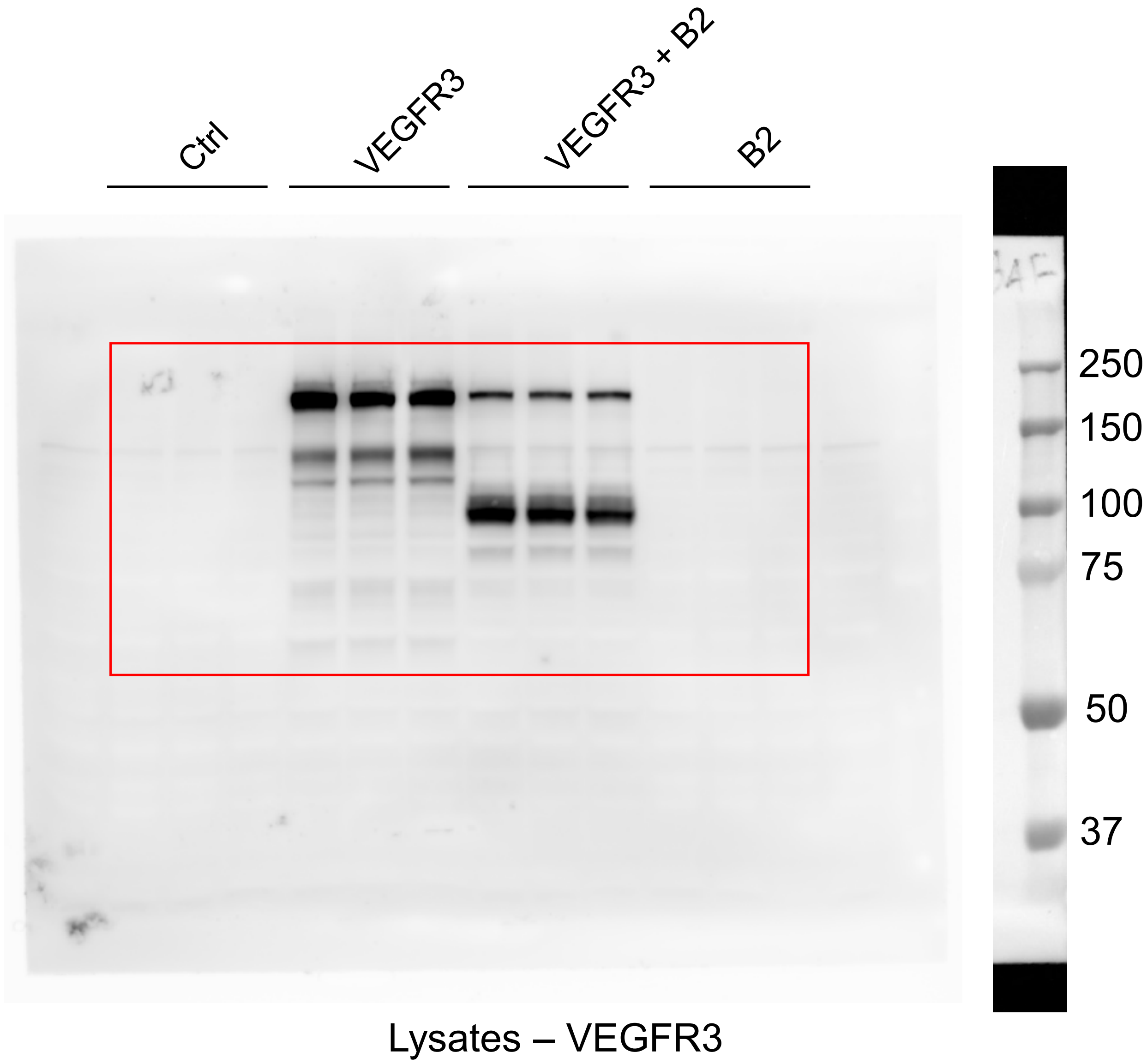

Full unedited blots for Figure 2B

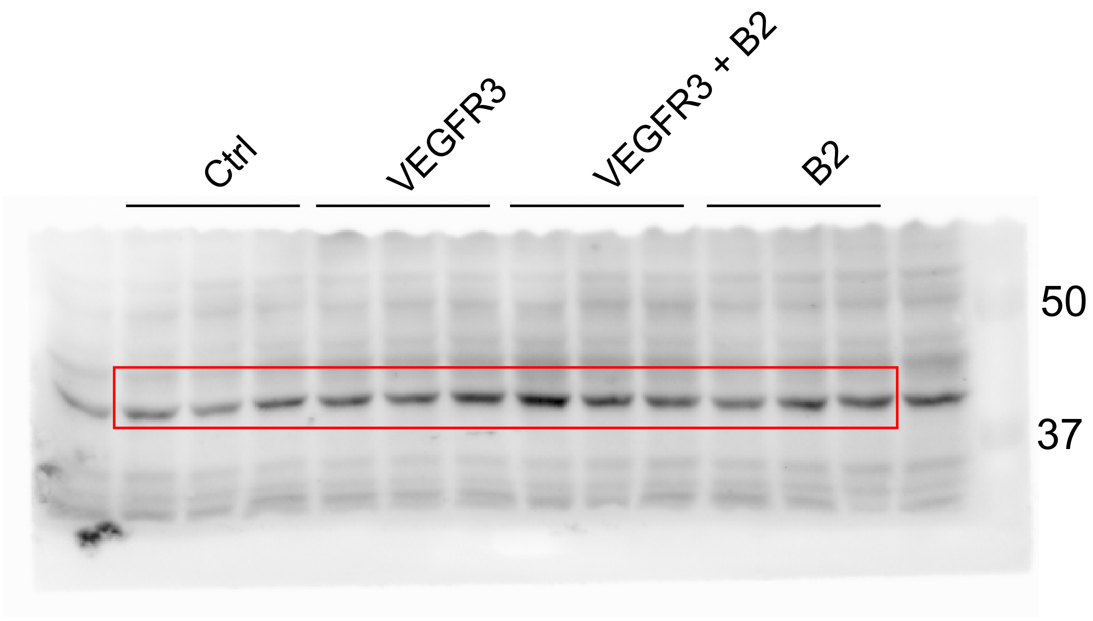

Lysates – Actin

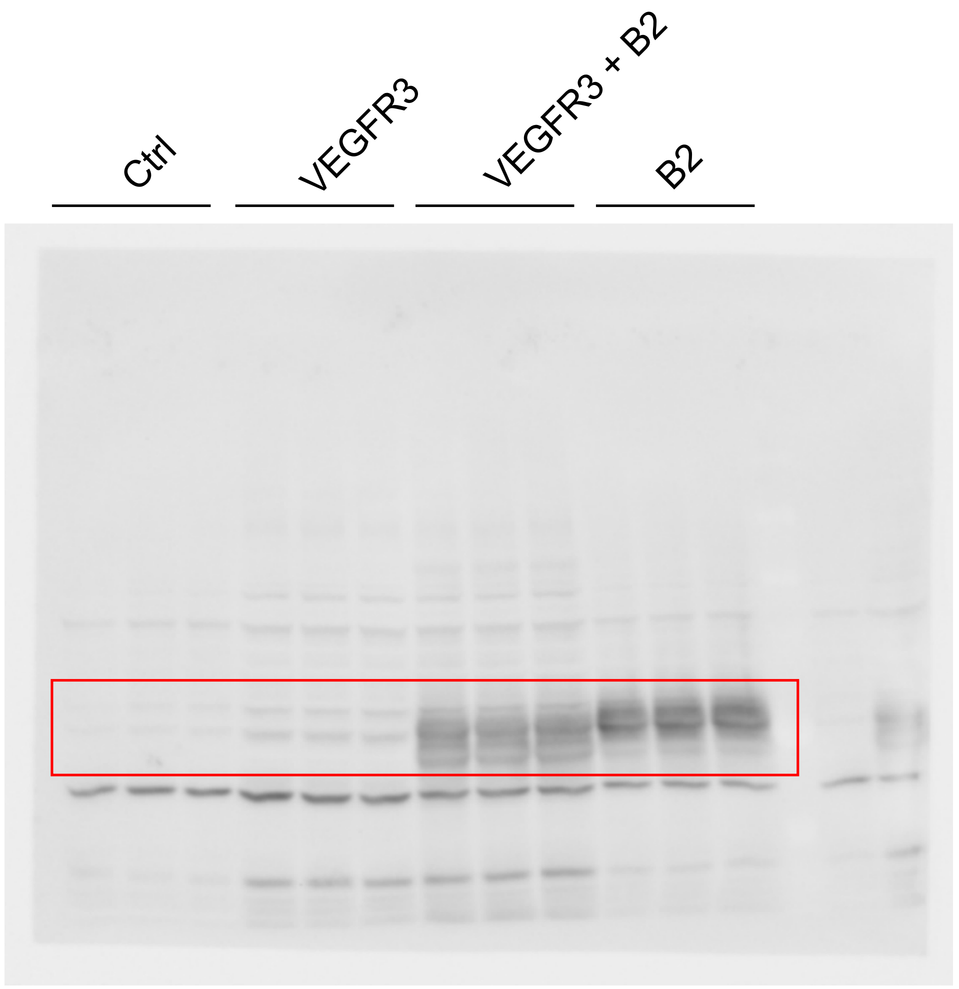

Lysates – BACE2

Full unedited blots for Figure 2B

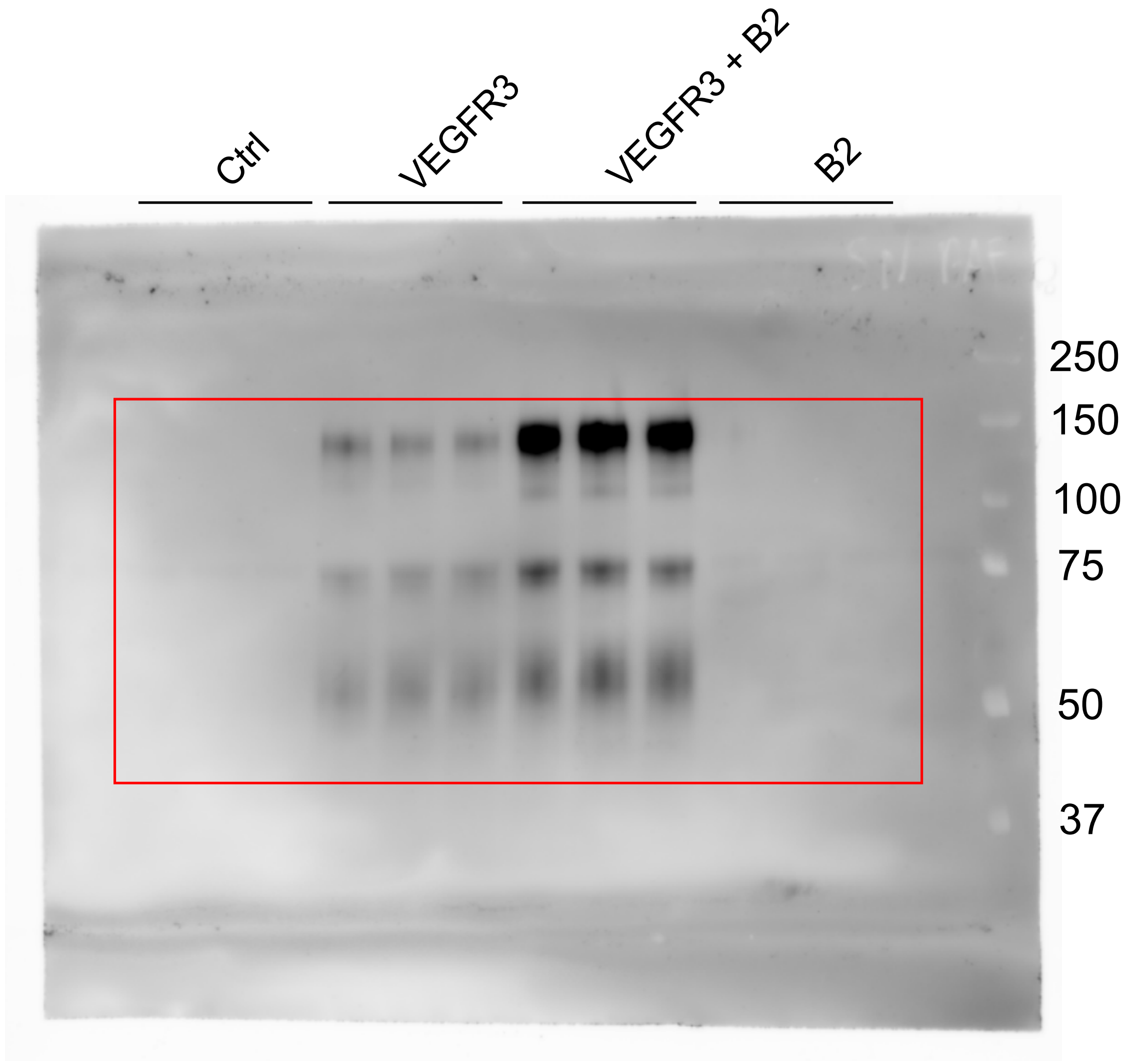

Media – VEGFR3

Full unedited blots for Figure 3A

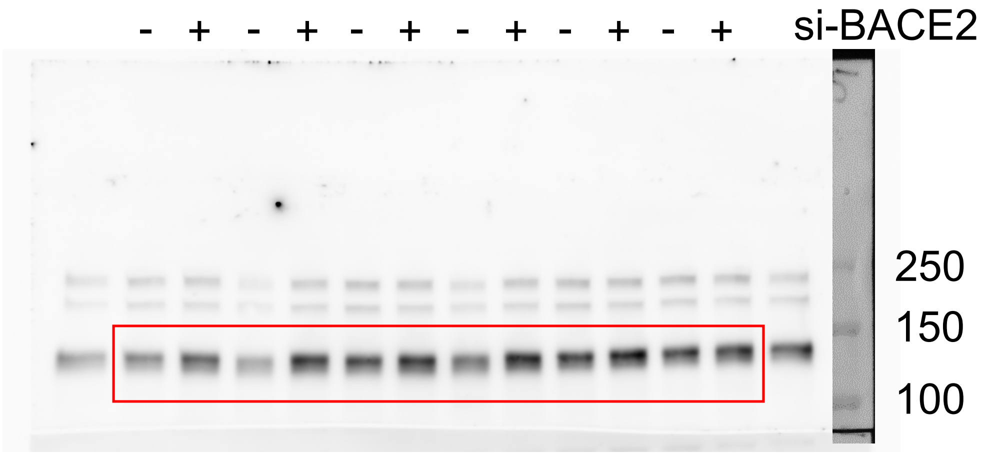

Lysates – VEGFR3

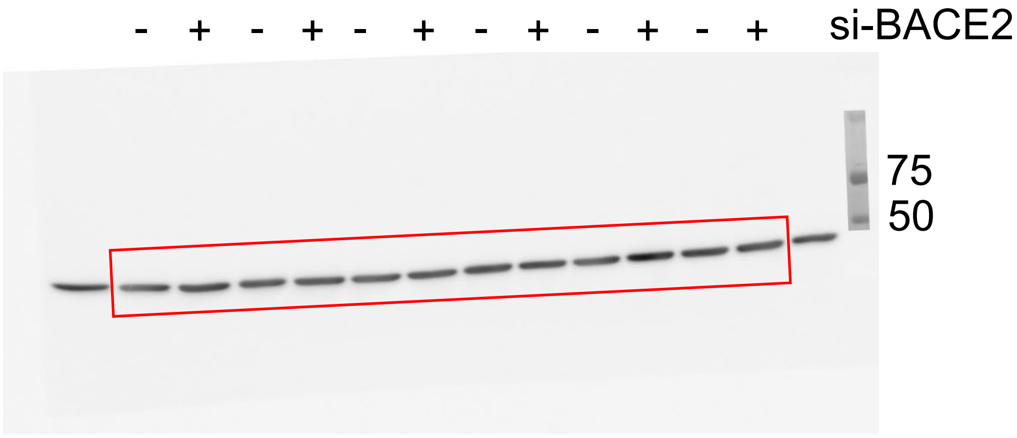

Lysates – Actin

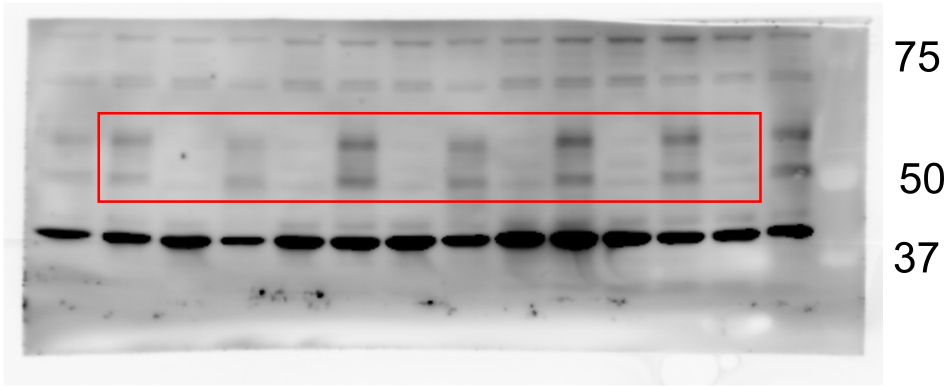

Lysates – BACE2

Full unedited blots for Figure 3A

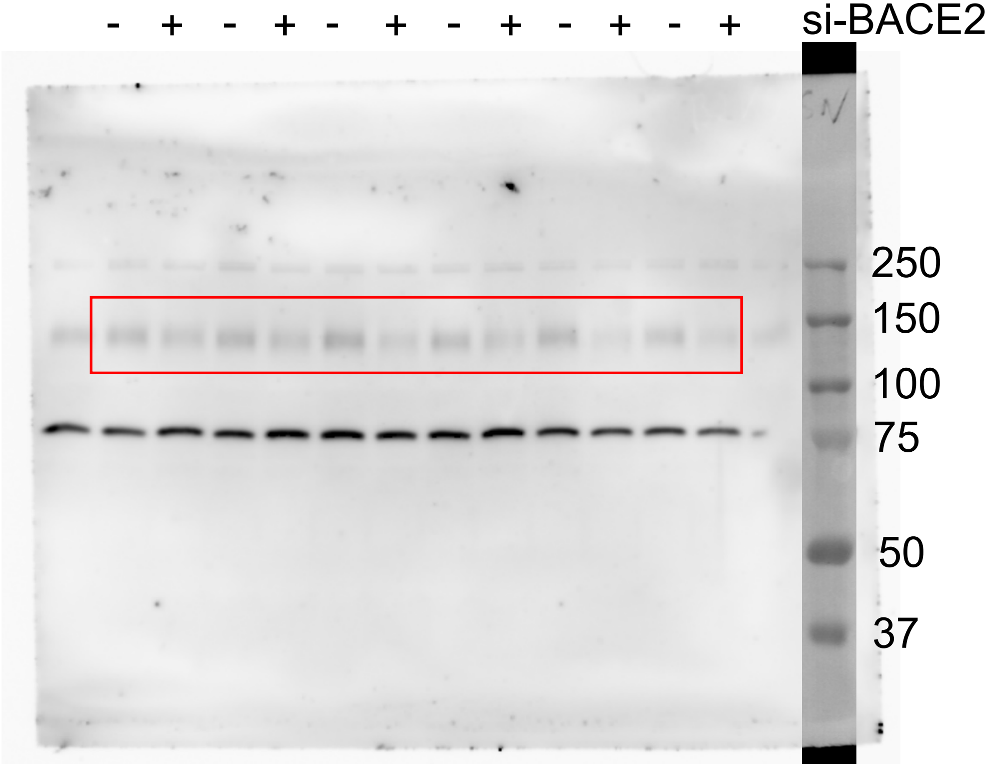

Media – VEGFR3

Full unedited blots for Figure 3A

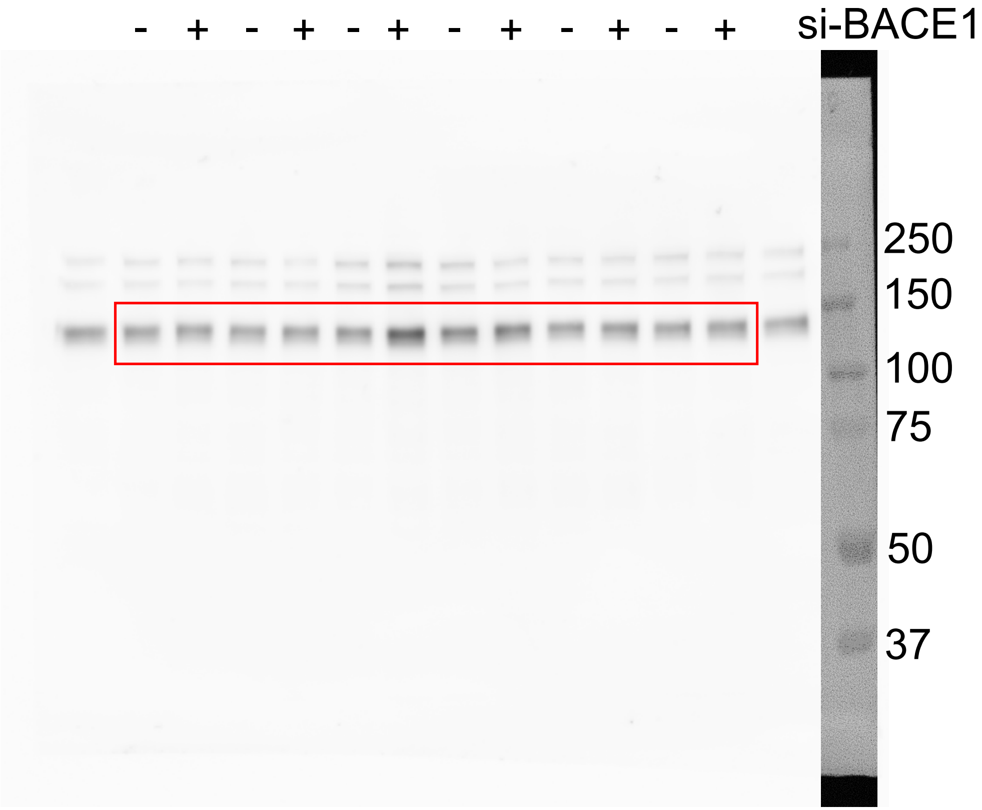

Lysates – VEGFR3

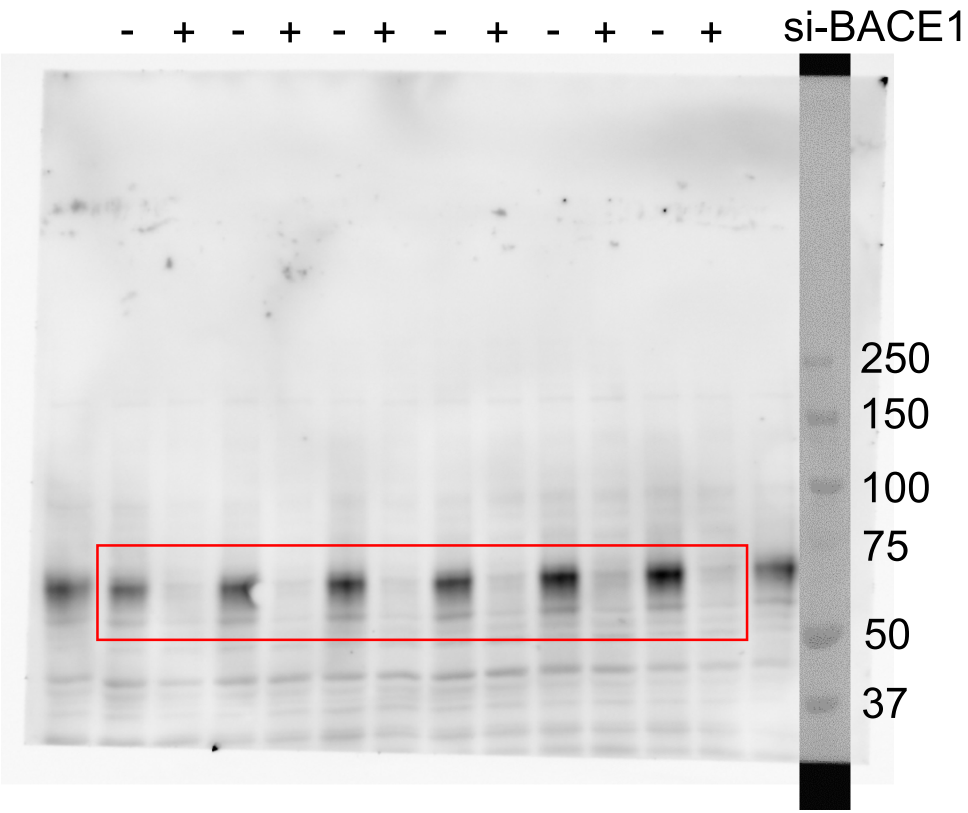

Lysates – BACE1

Full unedited blots for Figure 3A

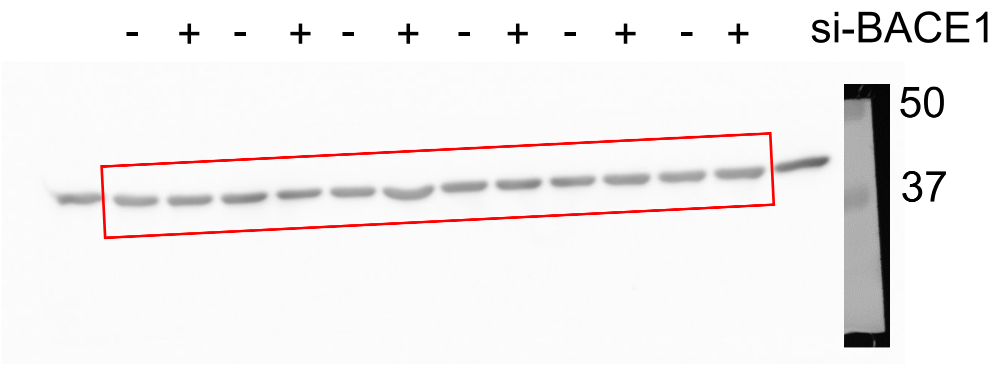

Lysates – Actin

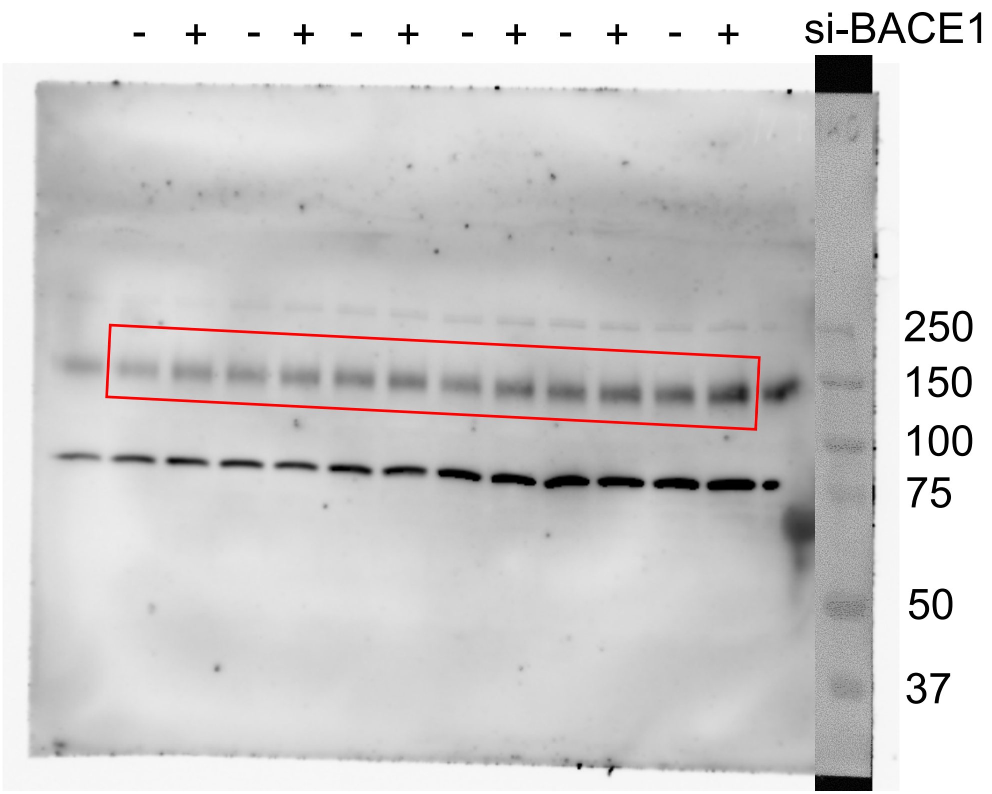

Media – VEGFR3

Full unedited blots for Figure 3C

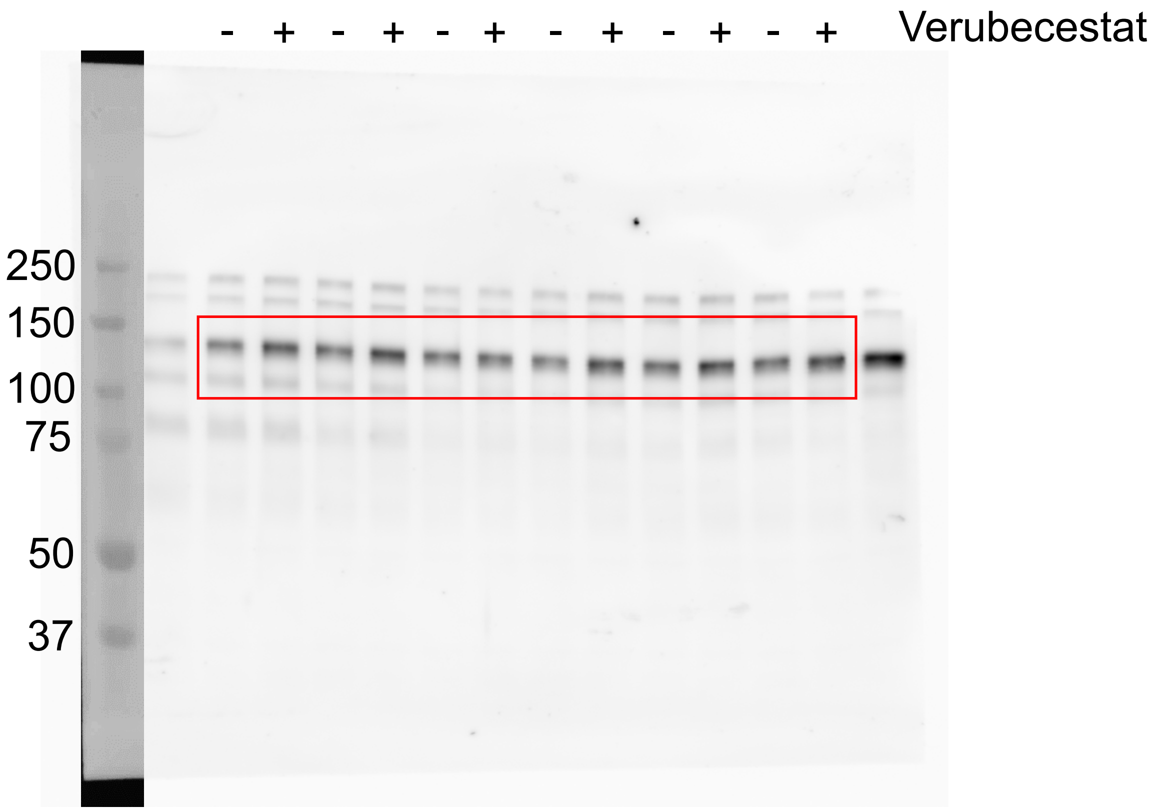

Lysates – VEGFR3

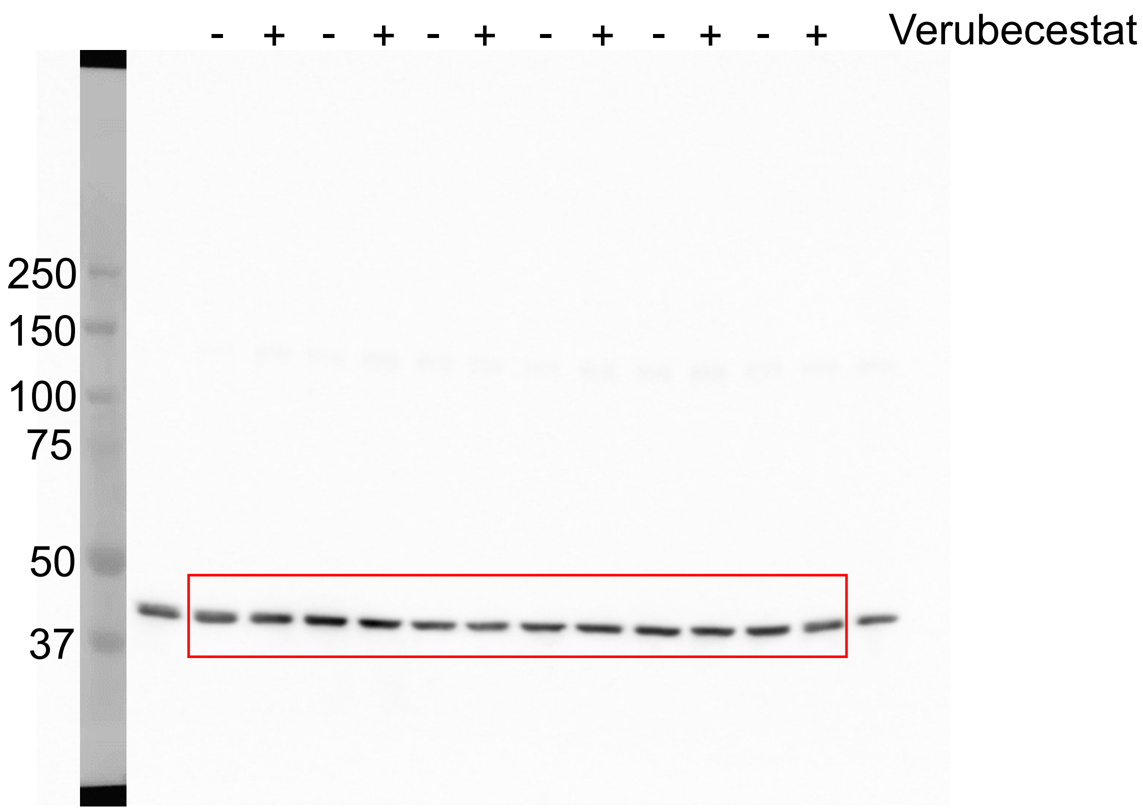

Lysates – Actin

Full unedited blots for Figure 3C

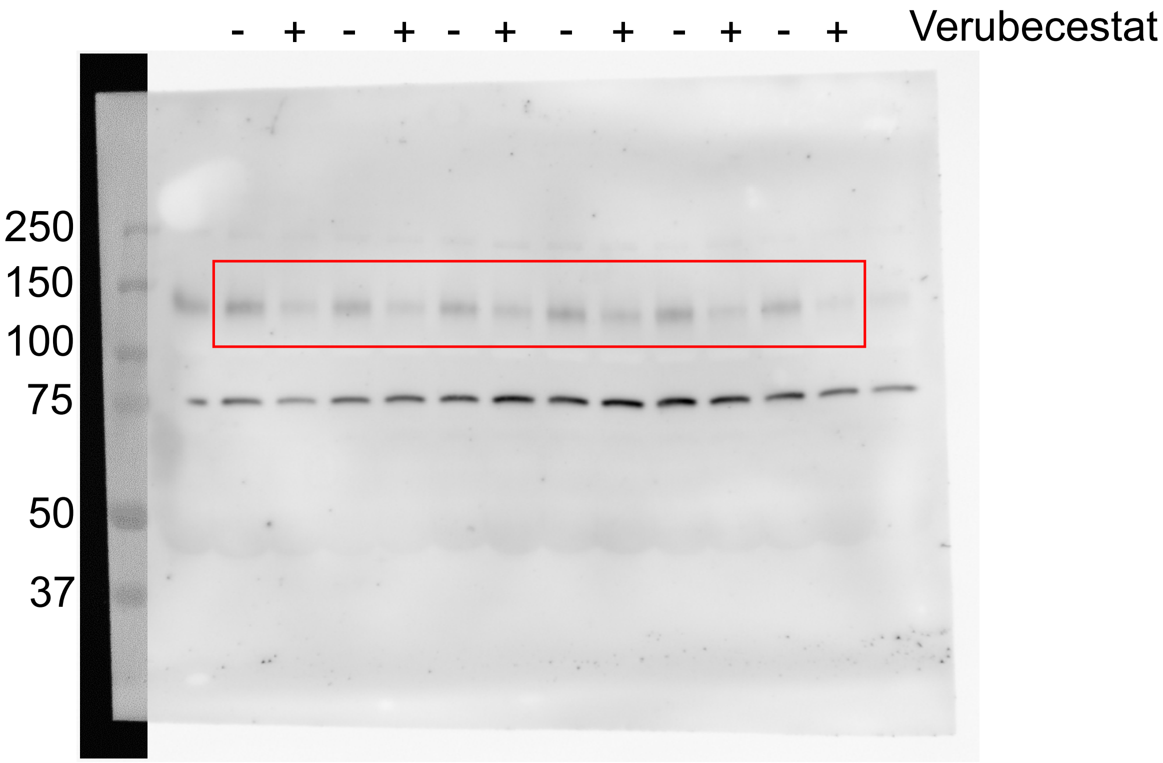

Media – VEGFR3

Full unedited blots for Supplementary Figure 1A

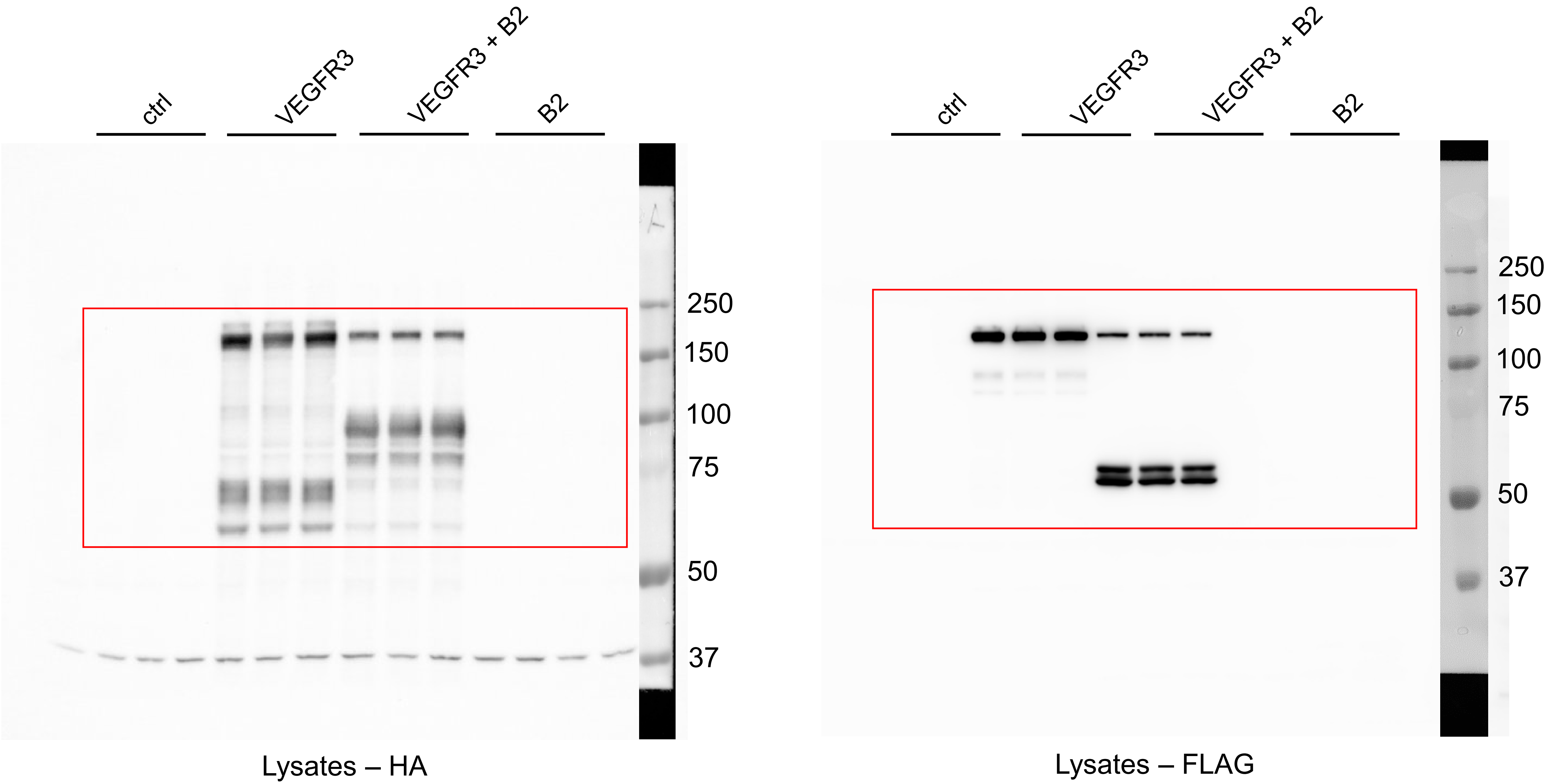

Full unedited blots for Supplementary Figure 1A

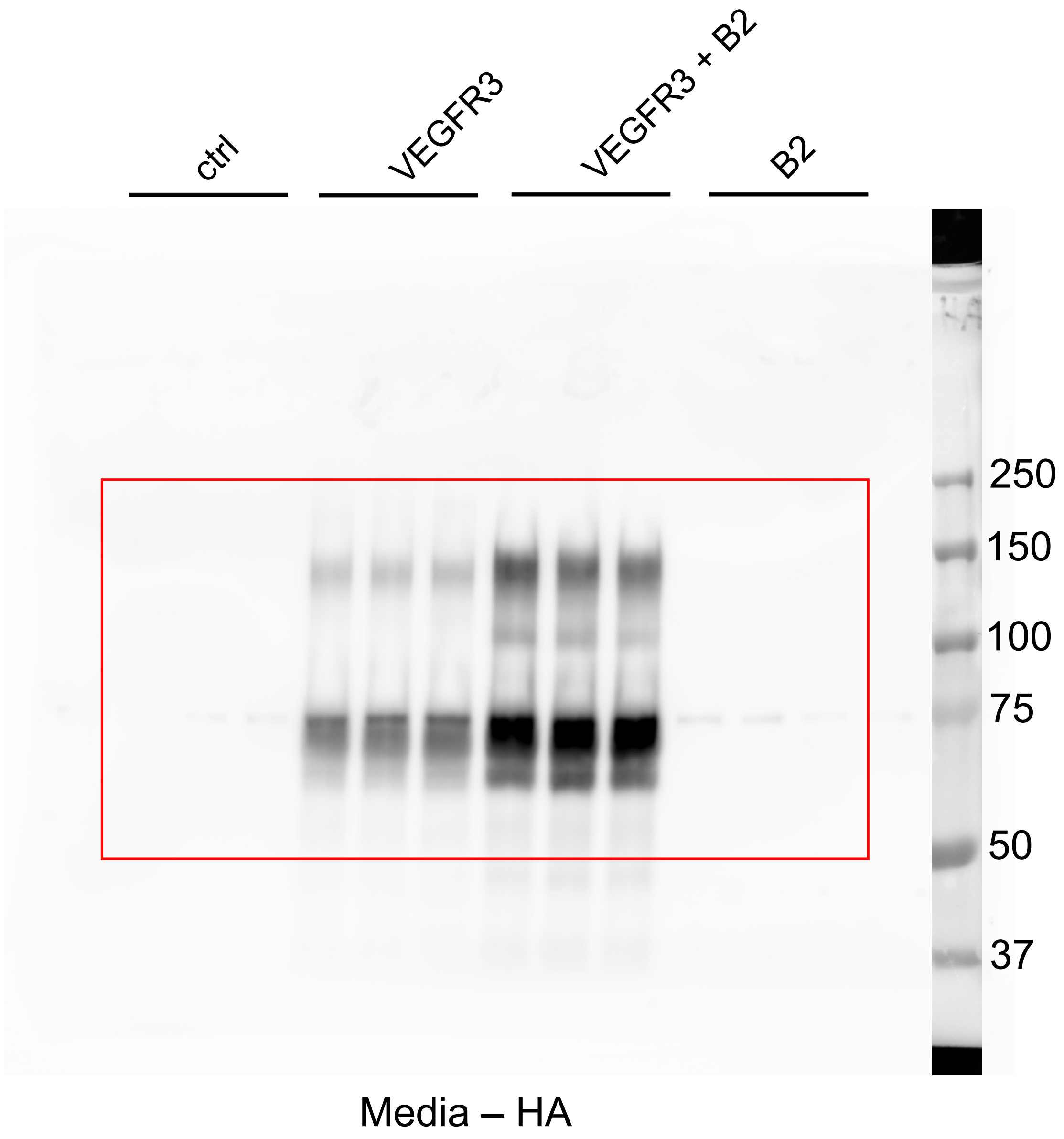

Full unedited blots for Supplementary Figure 1B

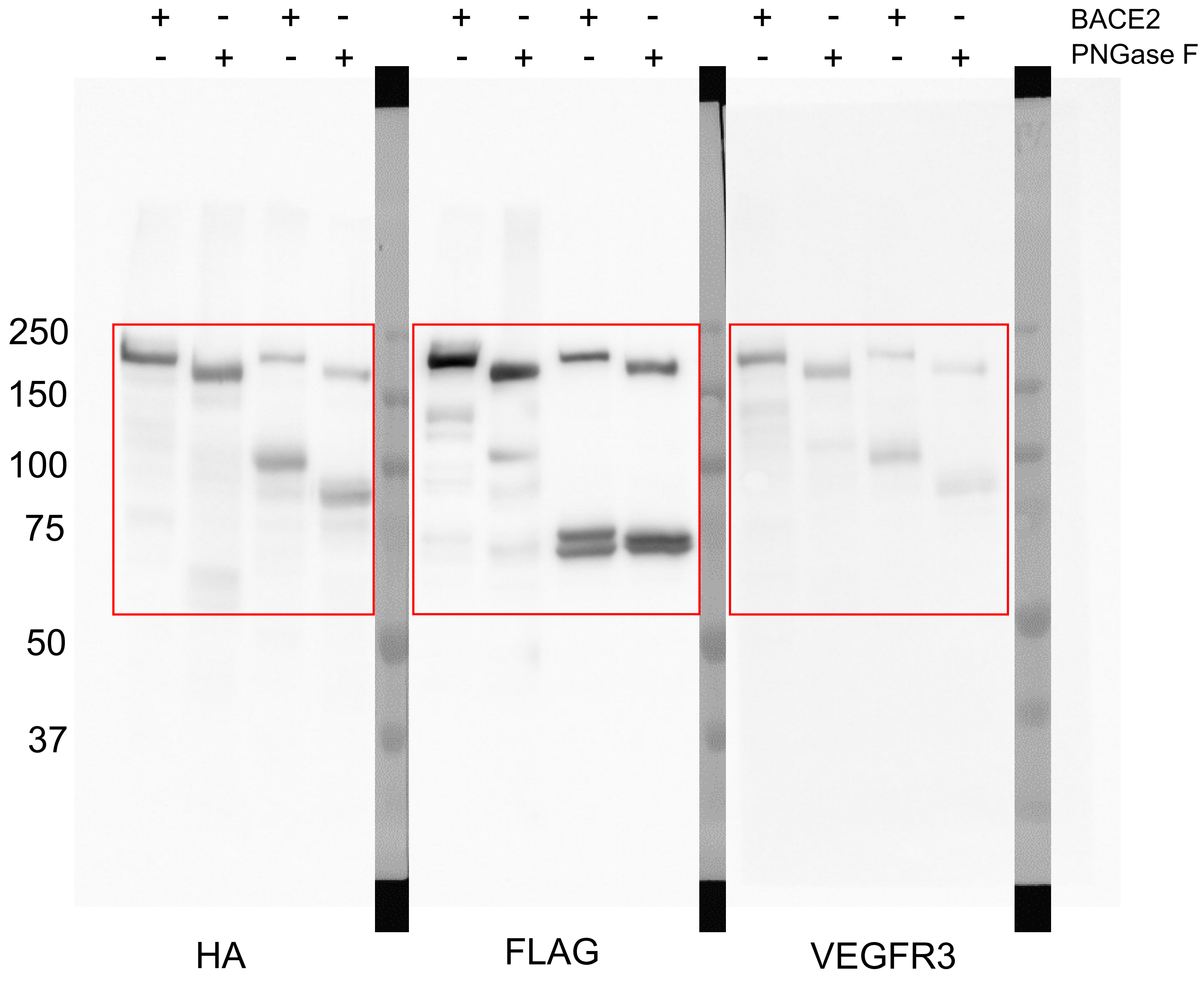

Full unedited blots for Supplementary Figure 2

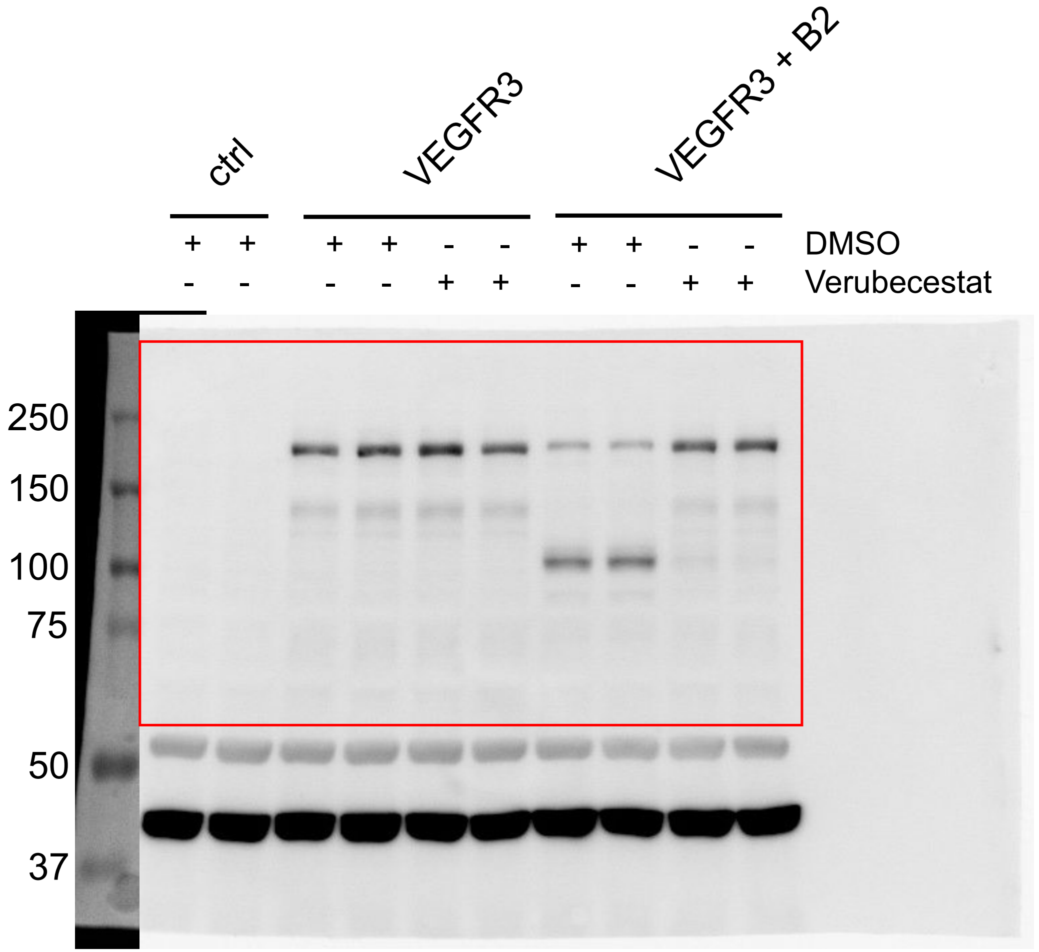

Lysates – VEGFR3

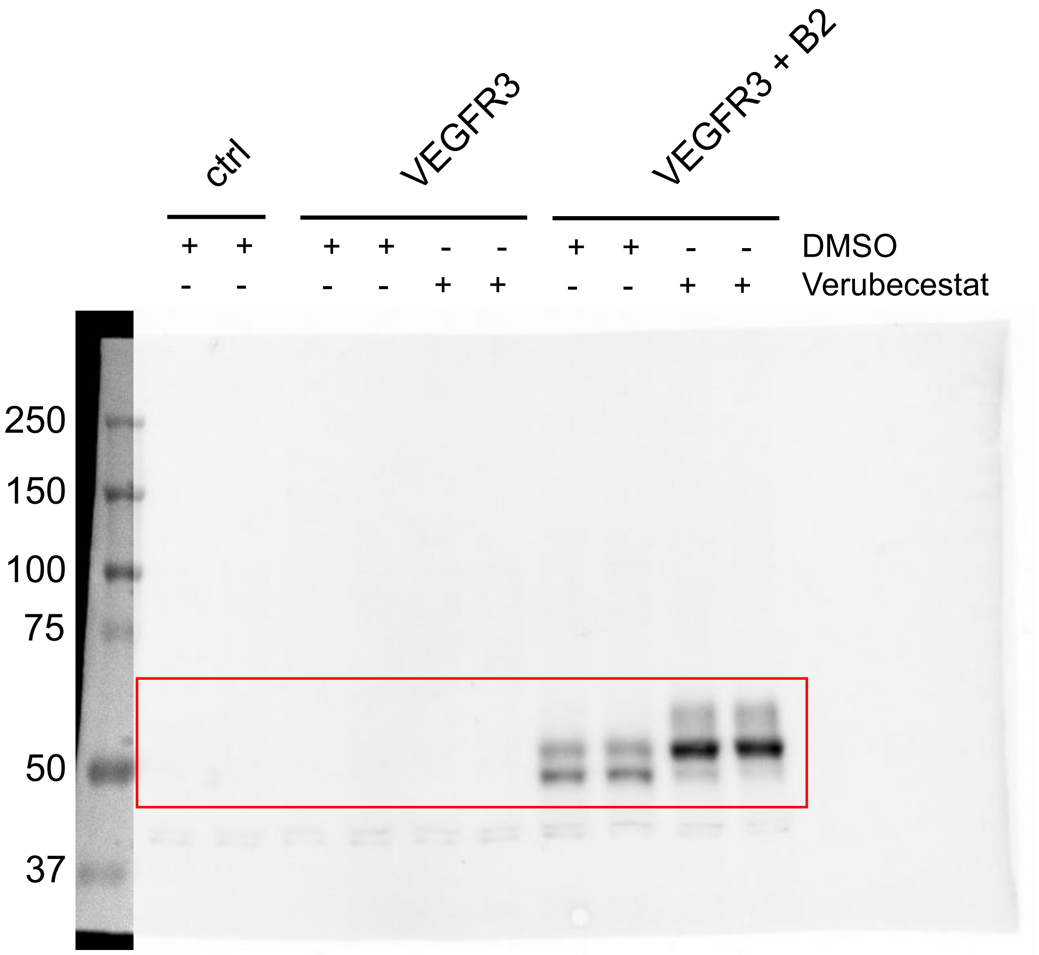

Lysates – BACE2

Full unedited blots for Supplementary Figure 2

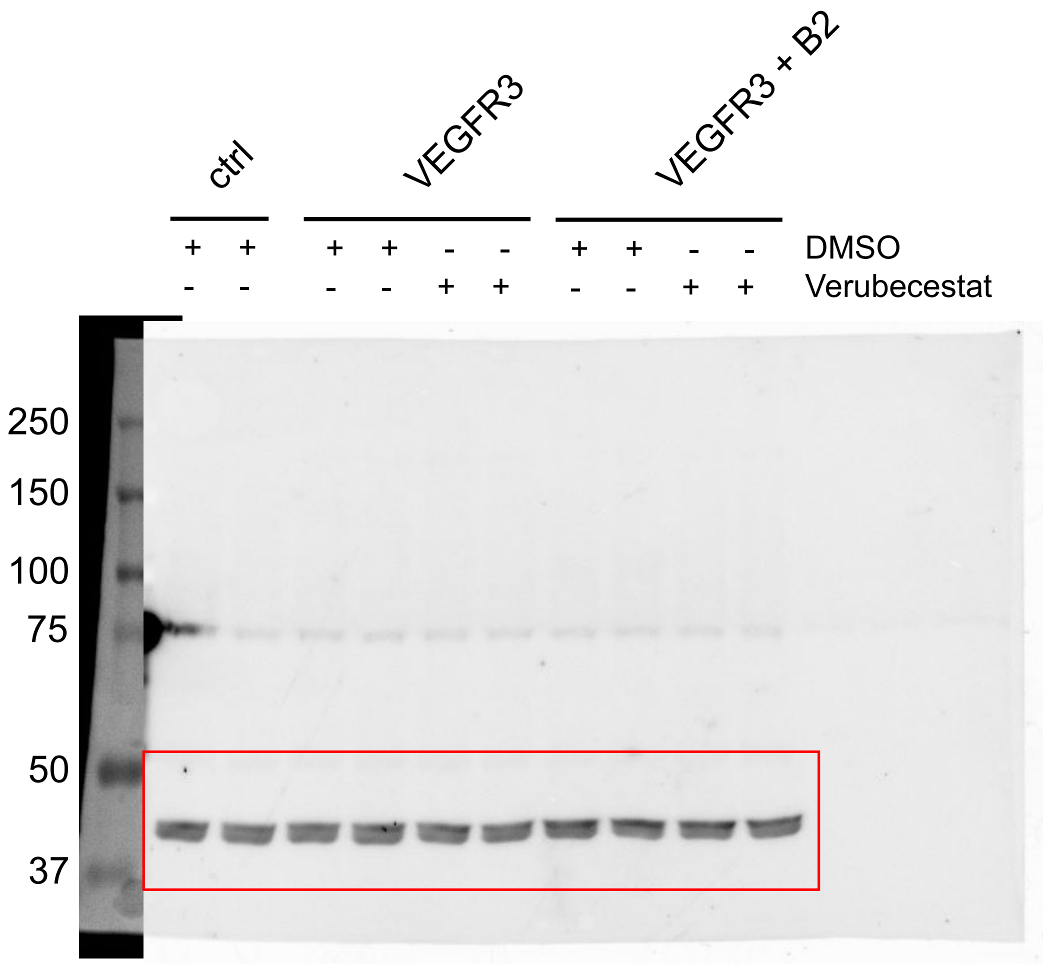

Lysates – Actin

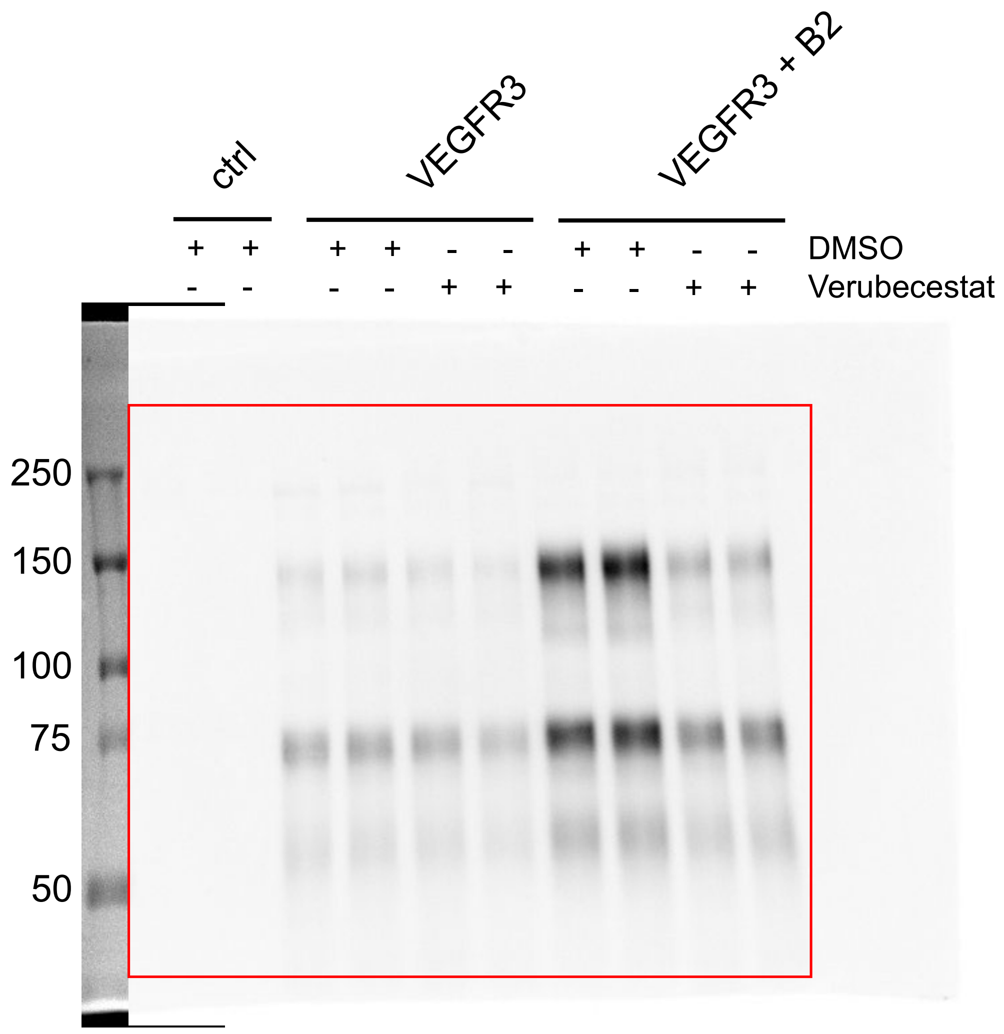

Media – VEGFR3

Full unedited blots for Supplementary Figure 3A

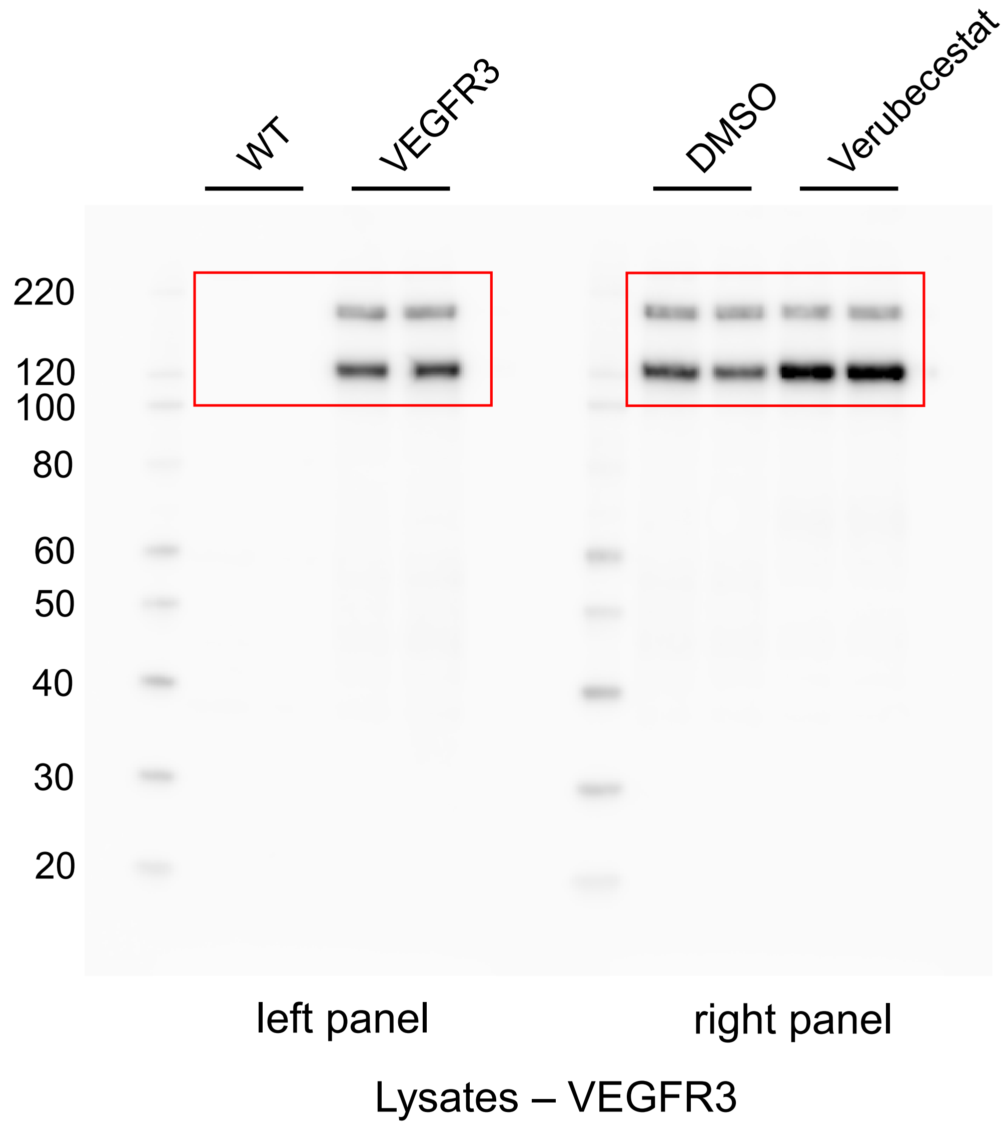

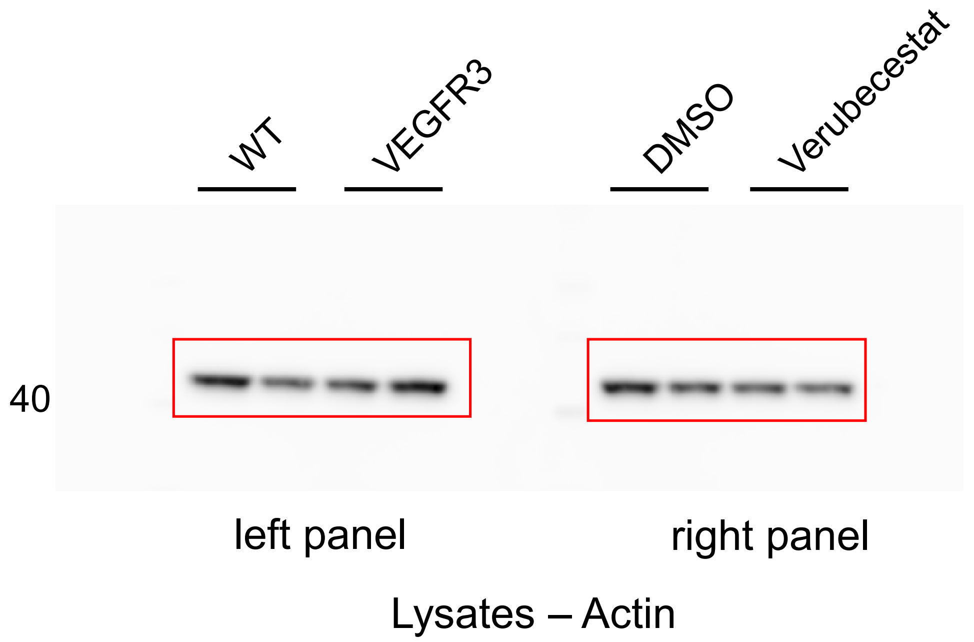

Full unedited blots for Supplementary Figure 3A

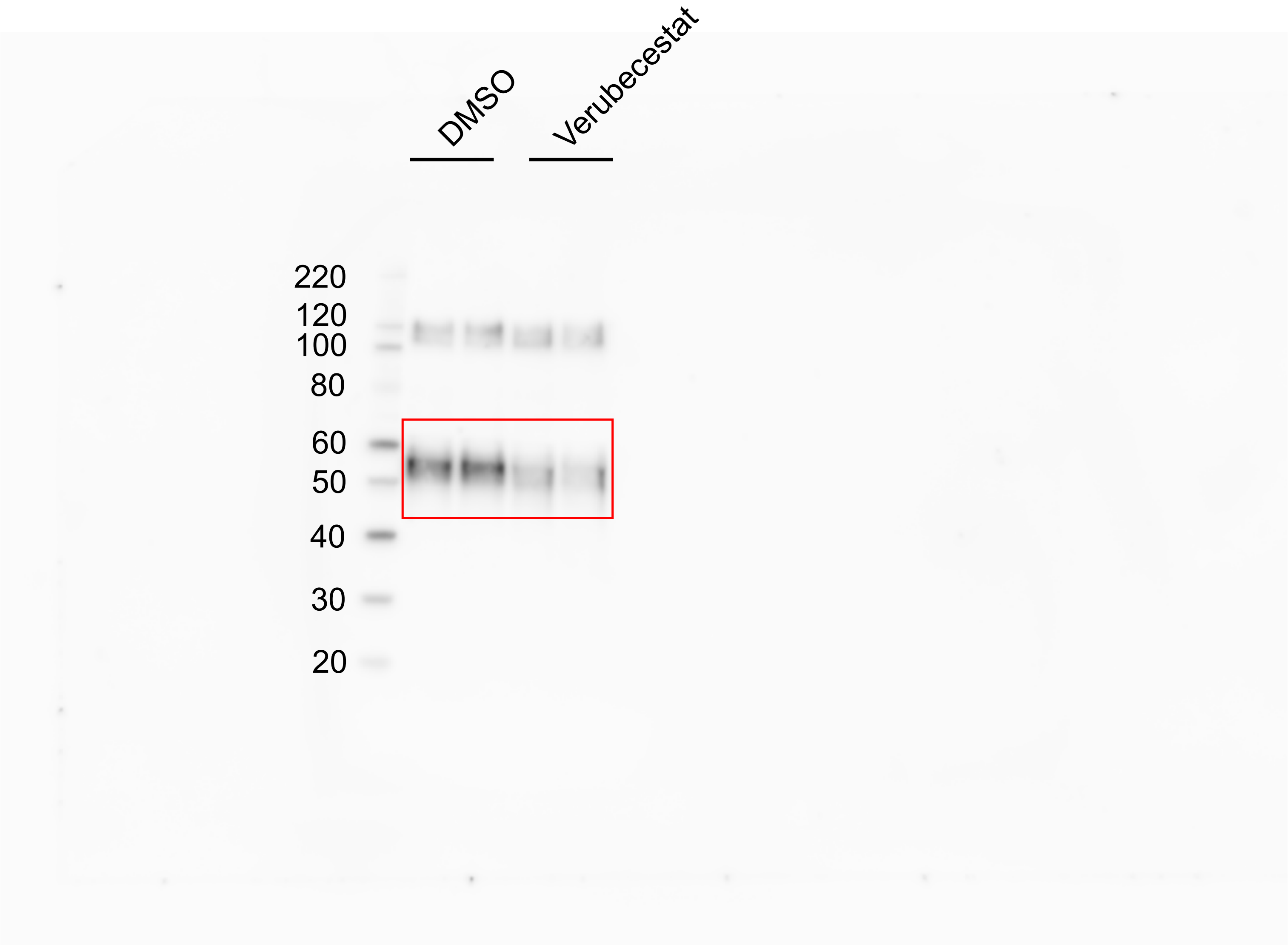

Media – VEGFR3

Full unedited blots for Supplementary Figure 4B

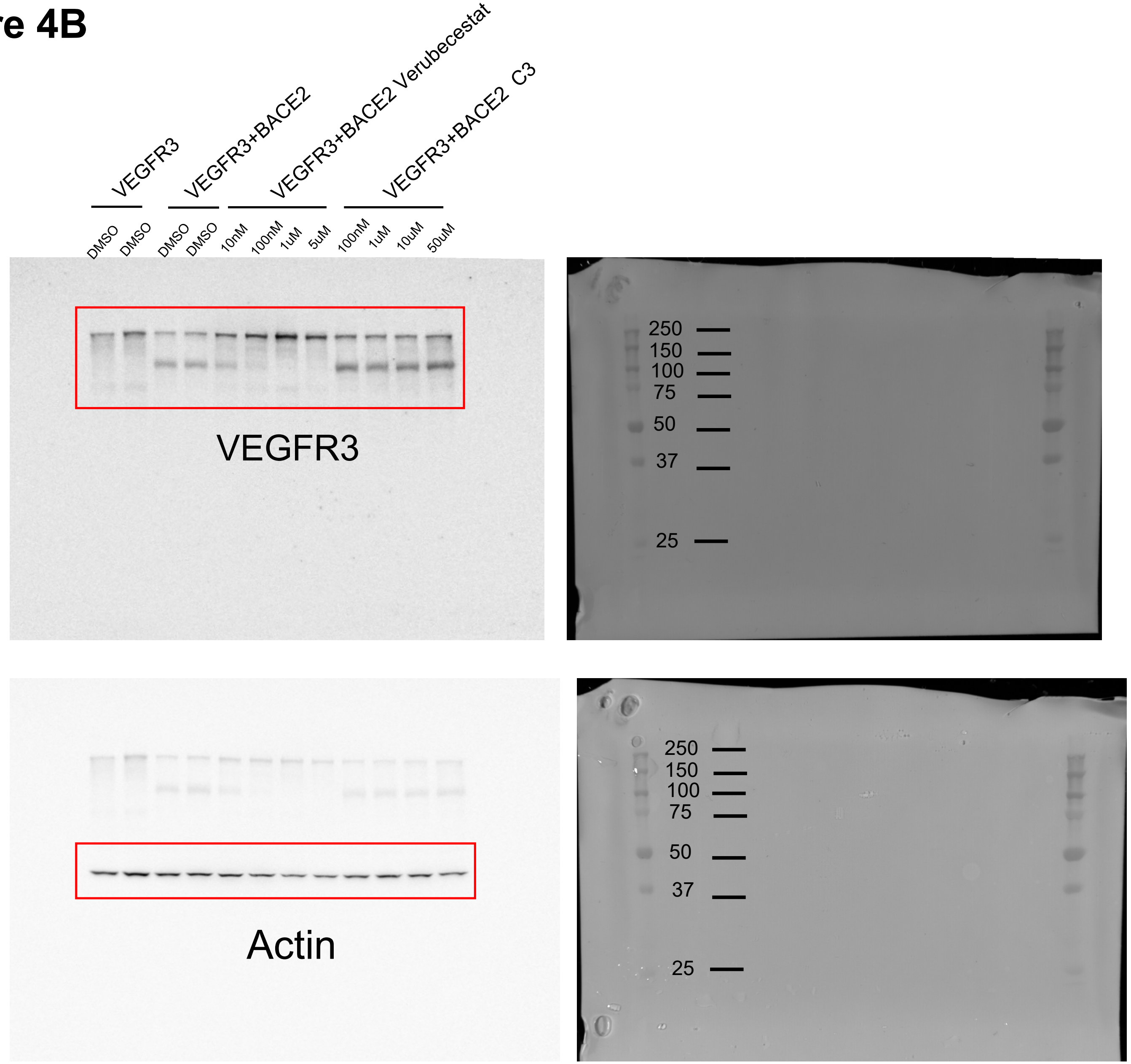

Full unedited blots for Supplementary Figure 4B

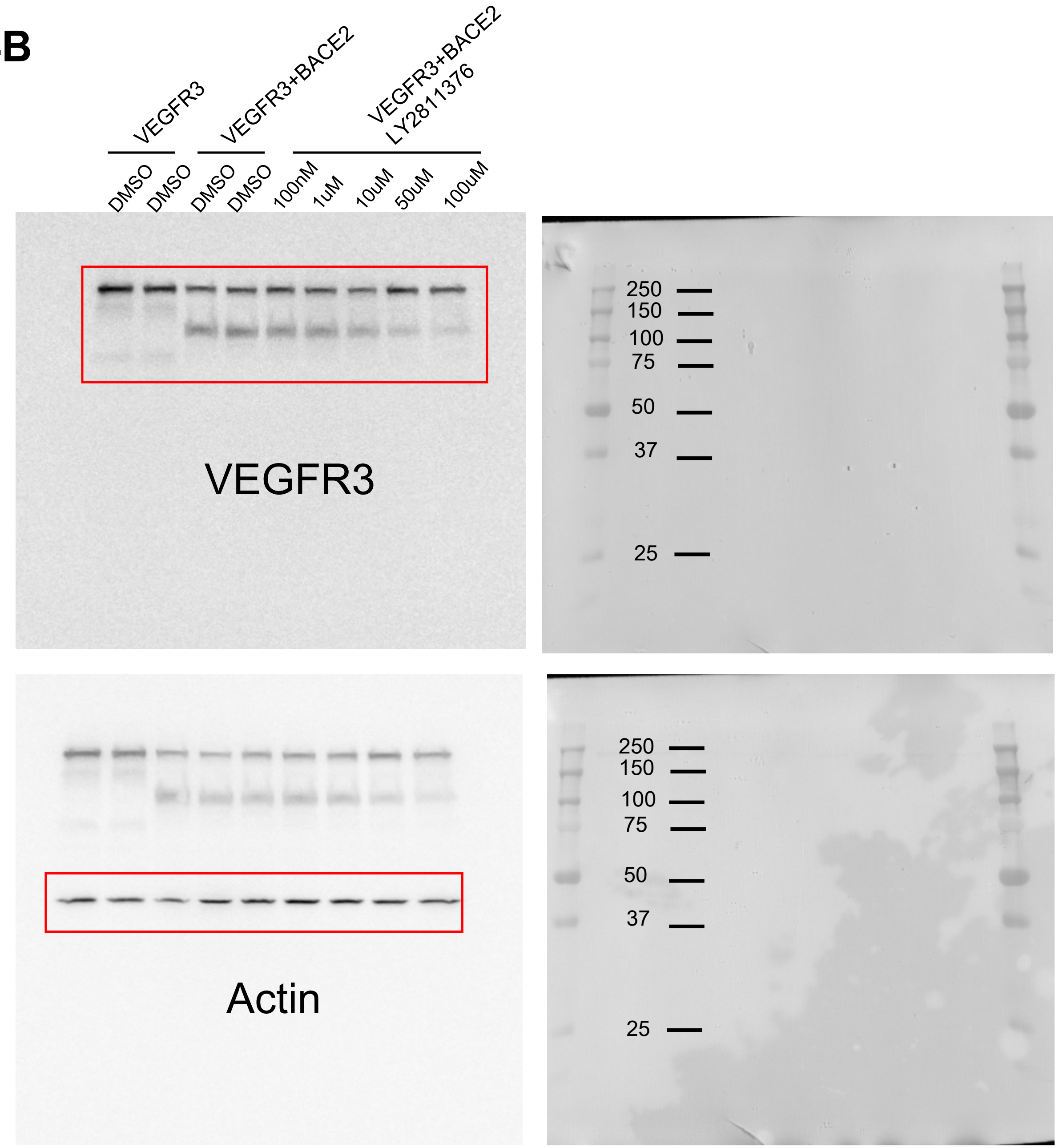

Full unedited blots for Supplementary Figure 6A

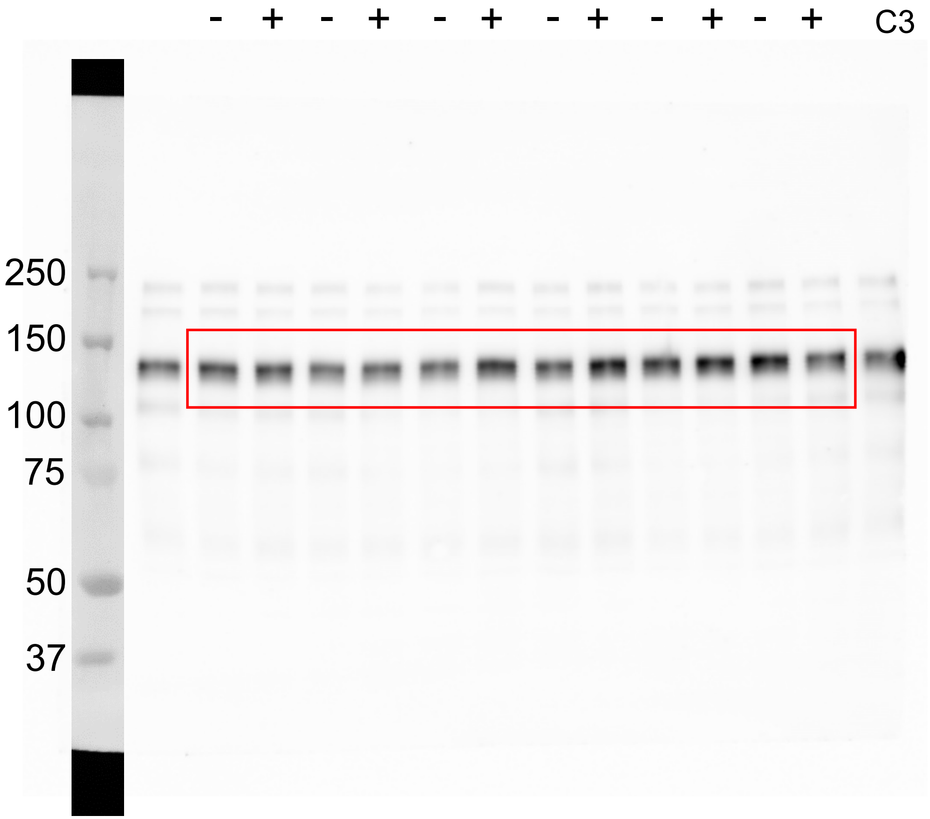

Lysates – VEGFR3

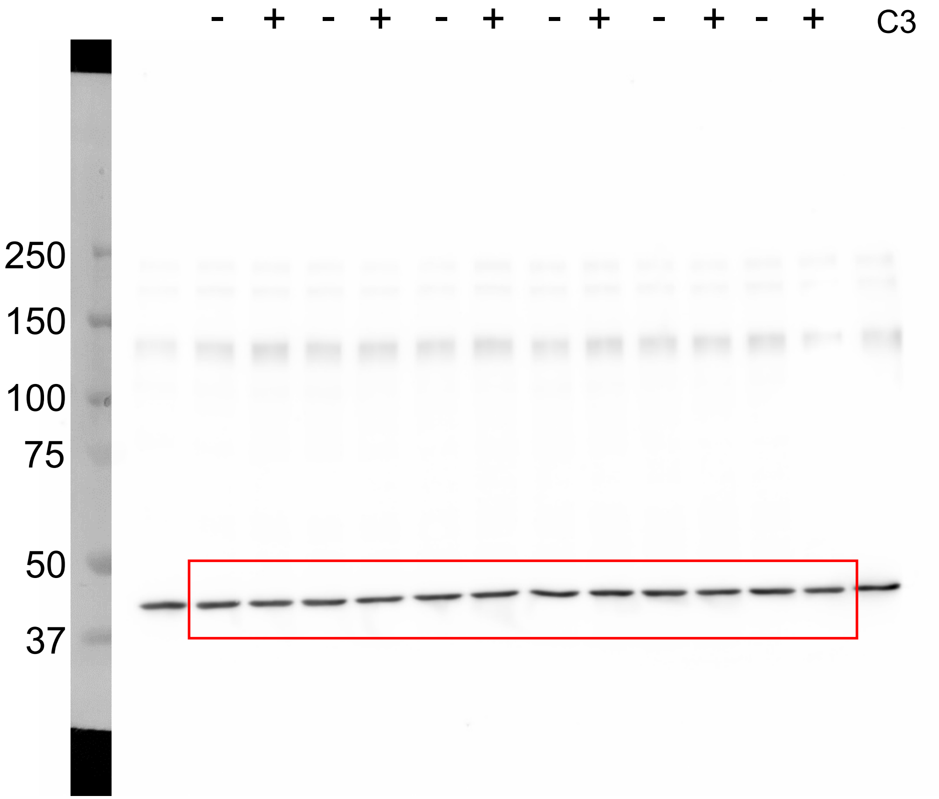

Lysates – Actin

Full unedited blots for Supplementary Figure 6A

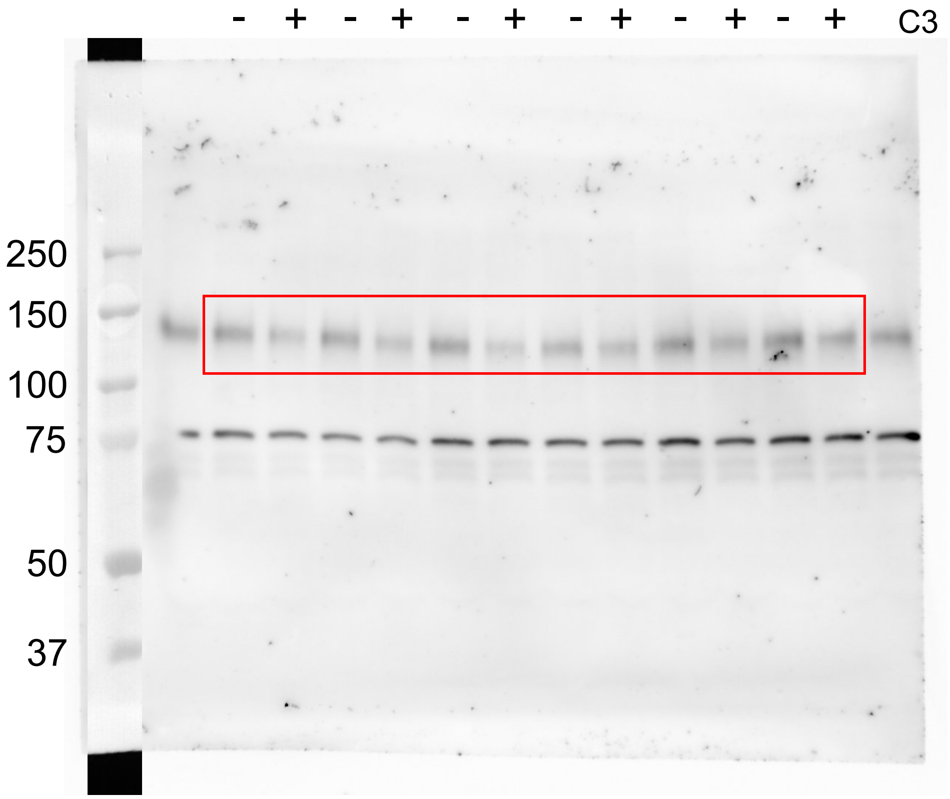

Media – VEGFR3
